# Historical Perspectives, Classification and Diagnostic Approaches of Inborn Errors of Metabolism: A Systematic Review and Meta-Analysis

**DOI:** 10.3390/metabo16070445

**Published:** 2026-06-25

**Authors:** Janvière Mutamuliza, Elizabeth Gori, Léon Mutesa, François-Guillaume Debray

**Affiliations:** 1 Center for Human Genetics, School of Medicine and Pharmacy, College of Medicine and Health Sciences, University of Rwanda, Kigali P.O. Box 4285, Rwanda; 2 Department of Human Genetics, Metabolic Unit, Centre Hospitalier Universitaire, University of Liege, Domaine Sart-Tilman Bât. B35, B-4000 Liege, Belgium; 3 Research Directorate, Rwanda Military Teaching Hospital, Kigali P.O. Box 3377, Rwanda; 4 Department of Medical Biochemistry, Molecular Biology & Genetics, College of Medicine and Health Sciences, University of Rwanda, Butare P.O. Box 117, Rwanda

**Keywords:** inborn errors of metabolism, systematic review, classification systems, diagnosis accuracy, tandem mass spectrometry, next-generation sequencing, newborn screening, metabolomics, historical perspectives, artificial intelligence

## Abstract

**Highlights:**

**What are the main findings?**

- **Diagnostic Technologies Demonstrate Excellent Performance:** Tandem mass spectrometry (MS/MS) achieved a pooled sensitivity of 99.1% and specificity of 99.8% for newborn screening of inborn errors of metabolism (IEMs) across 54 studies (8.23 million individuals, 35 countries). In comparison, next-generation sequencing (NGS) yielded a diagnostic rate of 42.8% in suspected cases—rising to 58–65% when integrated with multi-omics. Emerging artificial intelligence (AI)-powered tools achieved an area under the curve (AUC) > 0.95 for specific IEMs (e.g., glycogen storage disease type Ia (GSD Ia): 0.955; citrin deficiency: 0.993).
- **IEM Prevalence and Classification Are Well-Defined but Underappreciated:** The pooled global IEM prevalence is 50.9 per 100,000 live births (~1 in 1965), with 16 historical milestones identified from Garrod’s 1902 “chemical individuality” concept to 2025 AI-powered diagnostics. Four major classification systems were characterized: pathophysiological, biochemical pathway-based, organelle-based, and Society for the Study of Inborn Errors of Metabolism (SSIEM) nosology, each serving complementary clinical and research purposes.

**What are the implications of the main findings?**

- **Clinical Practice: Tiered, Technology-Integrated Diagnostic Algorithms Are Now Essential:** The high performance of MS/MS (99.1% sensitivity) supports its continued role as the cornerstone of universal newborn screening, but the low positive predictive value (PPV: 12.8%) mandates second-tier confirmatory testing. NGS should be systematically integrated into diagnostic workflows for symptomatic cases, and AI tools, while promising, require mandatory human oversight, external validation, and explainability frameworks before programmatic clinical adoption. Standardized use of SSIEM nosology across centers is recommended to harmonize diagnosis, reporting, and research.
- **Public Health and Policy: Equity, Regulation, and AI Governance Must Be Prioritized**: With IEM prevalence at 50.9 per 100,000 live births across 35 countries and diagnostic delay historically averaging 15 years (reducible to 2.3 years with AI-assisted screening), there is an urgent need for: (i) equitable global access to MS/MS and NGS screening regardless of geography or socioeconomic status; (ii) clear regulatory pathways governing AI diagnostic tools in rare metabolic diseases; and (iii) prospective multicenter validation of artificial intelligence/machine learning (AI/ML) classifiers across diverse populations before policy endorsement.

**Abstract:**

**Background:** Inborn errors of metabolism (IEMs) represent a diverse group of genetic disorders affecting biochemical pathways. Despite advances in diagnostic technologies, comprehensive understanding of their historical evolution, classification systems, and diagnostic approaches remains fragmented. **Objectives:** This systematic review and meta-analysis aimed to synthesize evidence on the historical development, classification frameworks, and diagnostic modalities for IEMs, diagnostic accuracy, and prevalence estimates, providing a comprehensive resource for clinicians and researchers. **Methods:** Following PRISMA 2020 guidelines, we conducted a systematic search of seven electronic databases (PubMed/MEDLINE, Embase, Scopus, Web of Science, Google Scholar, SciSpace and ArXiv) from January 2000 to March 2026. Studies addressing historical perspectives, classification systems, or diagnostic approaches for IEMs were included. Two independent reviewers performed screening, data extraction, and quality assessment. Meta-analyses were conducted using random-effects models for diagnostic accuracy and prevalence estimates. **Results**: From 1342 identified records, 54 studies met the inclusion criteria, encompassing 8,234,567 individuals across 35 countries. Historical analysis revealed 16 major milestones from Garrod’s 1902 “chemical individuality” concept to the current AI-powered diagnostics. Four major classification systems were identified: pathophysiological (intoxication, energy deficiency, complex molecule disorders), biochemical pathway (amino acid, organic acid, urea cycle, carbohydrate, fatty acid oxidation, mitochondrial, peroxisomal, lysosomal disorders), organelle-based, and the integrated Society for the Study of Inborn Errors of Metabolism (SSIEM) nosology. Meta-analysis demonstrated high diagnostic performance of tandem mass spectrometry (MS/MS) with a pooled sensitivity of 99.1% (95% CI: 98.6–99.5) and specificity of 99.8% (95% CI: 99.7–99.9%). The pooled global prevalence of IEMs was 50.9 per 100,000 live births (95% CI 45.2–56.8). Next-generation sequencing achieved a diagnostic yield of 42.8% (95% CI: 38.2–47.5%) in suspected cases. Emerging AI-powered diagnostic tools demonstrated high discrimination performance with area under the curve (AUC) values exceeding 0.95 for specific IEM, though external validation remains limited. Newborn screening expanded from single-disease to comprehensive panels detecting over 50 disorders. **Conclusions:** This comprehensive review demonstrates that IEMs have evolved from rare curiosities to systematically diagnosable conditions through technological advances. Integration of metabolomics, genomics, proteomics and artificial intelligence promises further diagnostic improvements. Standardized classification systems and evidence-based diagnostic algorithms are essential for optimal patient care. Future directions include artificial intelligence-enhanced diagnostics, expanded screening, and personalized medicine approaches.

## 1. Introduction

### 1.1. Background

Inborn errors of metabolism (IEMs) constitute a clinically and biochemically heterogeneous group of genetic disorders resulting from defects in metabolic pathways [1,2]. First conceptualized by Sir Archibald Garrod in his landmark 1902 Croonian Lectures on “The Incidence of Alkaptonuria: A Study in Chemical Individuality” [3], IEMs have evolved from medical curiosities to systematically diagnosable conditions affecting approximately 1 in 2000 live births globally [4,5]. The field has witnessed remarkable transformation over the past century, driven by advances in biochemical analysis, molecular genetics, and diagnostic technologies [6,7].

The clinical significance of IEMs extends beyond their individual rarity, as collectively they represent a substantial burden on healthcare systems and affected families [8,9]. Many IEMs present in the neonatal period with nonspecific symptoms such as poor feeding, vomiting, lethargy, and seizures, making timely diagnosis challenging yet critical for preventing irreversible neurological damage and death [10,11]. The advent of newborn screening programs, beginning with Robert Guthrie’s bacterial inhibition assay for phenylketonuria (PKU) in 1963 [12], has revolutionized early detection and intervention for selected IEMs [13,14].

Over the past two decades, technological innovations have dramatically expanded diagnostic capabilities. These advances have transformed diagnostic approaches from single-disorder testing to high-throughput, multi-platform analyses capable of simultaneously assessing numerous metabolites and genetic variants. Among the most influential developments, tandem mass spectrometry (MS/MS) enables simultaneous detection of multiple metabolites from a single dried blood spot [15,16], while next-generation sequencing (NGS) technologies facilitate comprehensive genomic analysis [17,18]. More recently, advanced metabolomics platforms, including gas chromatography–mass spectrometry (GC-MS), liquid chromatography–mass spectrometry (LC-MS), and nuclear magnetic resonance (NMR) spectroscopy, provide unprecedented insights into metabolic phenotypes [19,20]. The integration of artificial intelligence and machine learning into diagnostic workflows represents the latest frontier, promising enhanced pattern recognition, predictive modeling, and clinical decision support [21,22].

### 1.2. Evolution of IEM Classification and Challenges

The classification of IEMs has evolved considerably since Garrod’s initial descriptions. Early classification schemes focused on clinical phenotypes or affected organ systems. For example, conditions presenting with developmental delay, seizures, or neurodegeneration were categorized as neurological metabolic disorders, whereas disorders associated with hepatomegaly, liver failure, or hypoglycaemia were grouped as hepatic metabolic diseases [23,24]. Subsequent approaches emphasized biochemical pathways (e.g., amino acid disorders, organic acidemias, fatty acid oxidation defects) [25,26] or subcellular localization (e.g., mitochondrial disorders, peroxisomal disorders, lysosomal storage diseases) [27,28]. The Society for the Study of Inborn Errors of Metabolism (SSIEM) has developed a comprehensive nosology integrating multiple classification dimensions [29,30].

However, classification challenges persist due to phenotypic heterogeneity, overlapping biochemical features, and expanding molecular understanding [31,32]. Some disorders affect multiple pathways or organelles, while others present with variable phenotypes depending on residual enzyme activity [33,34]. The discovery of new disease genes and mechanisms continues to refine classification systems [35,36].

### 1.3. Diagnostic Landscape and Gaps

The diagnostic approach to IEMs has become increasingly complex, requiring integration of clinical assessment, biochemical testing, and molecular analysis [37,38]. Newborn screening programs vary widely in scope, with some jurisdictions screening for over 50 conditions while others test for fewer than 10 [39,40]. Diagnostic algorithms for symptomatic individuals must balance sensitivity, specificity, cost, and turnaround time [41,42].

Despite technological advances, significant diagnostic gaps remain. Many IEMs lack specific biomarkers, requiring functional enzyme assays or molecular testing for definitive diagnosis [43,44]. Interpretation of genomic variants of uncertain significance poses ongoing challenges [45,46]. Access to specialized metabolic testing varies globally, with resource-limited settings facing particular barriers [47,48]. The integration of multi-omics data and artificial intelligence into clinical practice remains in the early stages [49,50].

### 1.4. Rationale for Systematic Review

While numerous reviews have addressed specific aspects of IEMs, a comprehensive systematic synthesis integrating historical perspectives, classification systems, and diagnostic approaches is lacking. Previous reviews have focused on individual disease categories [51,52], specific diagnostic modalities [53,54], or regional screening programs [55,56]. There is a notable absence of systematic synthesis that integrates evidence across these domains while incorporating recent advances in metabolomics, genomics, and artificial intelligence.

This systematic review and meta-analysis addresses this gap by synthesizing evidence on: (1) historical milestones in IEM discovery and diagnosis; (2) evolution and comparison of classification systems; (3) diagnostic accuracy of screening and confirmatory tests; (4) prevalence estimates across populations; and (5) emerging diagnostic technologies including AI-powered tools. By providing (6) a comprehensive evidence base, this review aims to inform clinical practice, guide research priorities, and support policy decisions regarding screening programs and diagnostic algorithms.

### 1.5. Objectives

The primary objectives of this systematic review and meta-analysis were as follows:

1. Historical perspective: Identify and synthesize major milestones in the discovery, understanding, and diagnosis of IEMs from Garrod’s early work to contemporary AI-powered diagnostics.
2. Classification systems: Compare and evaluate different classification frameworks for IEMs, including pathophysiological, biochemical, organelle-based, and integrated approaches.
3. Diagnostic accuracy: Conduct meta-analyses of diagnostic test performance for key modalities including tandem mass spectrometry, next-generation sequencing, and metabolomics platforms.
4. Prevalence estimation: Synthesize population-based prevalence data for IEMs overall and for specific disease categories across different geographic regions.
5. Emerging technologies: Evaluate evidence for novel diagnostic approaches including advanced metabolomics, multi-omics integration, and artificial intelligence applications.
6. Clinical implications: Provide evidence-based recommendations for diagnostic algorithms, screening program design, and future research directions.

## 2. Materials and Methods

### 2.1. Protocol and Registration

This systematic review was conducted in accordance with the Preferred Reporting Items for Systematic Reviews and Meta-Analyses (PRISMA) 2020 guidelines [57]; the PRISMA checklist is provided in Supplementary File S1. The protocol was prospectively developed and registered with PROSPERO (registration number: [CRD420261300478]) prior to study commencement. No major deviations from the registered protocol.

### 2.2. Search Strategy

A comprehensive literature search was conducted by using the electronic databases: PubMed/MEDLINE, Embase, Scopus, Web of Science, Google Scholar, ArXiv and SciSpace from 1 January 2000 to March 2026. Searches were conducted in two phases. Phase one covered six databases from January 2000 to December 2024 using three concept blocks combined with Boolean AND: (1) IEM population terms, (2) historical/classification context terms, and (3) diagnostic/screening modality terms. Phase two applied an AI/ML-focused update across SciSpace, Google Scholar, ArXiv and PubMed/MEDLINE to capture publications from January 2025 to March 2026. Grey literature was identified through reference list checking, forward citation searching, and expert recommendations.

The systematic search was restricted to studies published between January 2000 and March 2026. Some publications published before 2000 were cited selectively to provide historical context regarding the discovery, classification, and evolution of diagnostic approaches for inherited metabolic disorders; these references were not included in the systematic review or meta-analysis.

The full search strategies for each database, including all search terms, filters, and limits, are provided in Supplementary File S2.

### 2.3. Eligibility Criteria

Studies were selected according to predefined eligibility criteria using the PICOS (Population, Intervention/Exposure, Comparator, Outcomes, Study Design) framework:

**Population:** Studies involving individuals with confirmed or suspected IEMs, or population-based screening programs.

**Intervention/exposure:** Studies addressing historical perspectives, classification systems, or diagnostic approaches for IEMs.

**Comparator:** For diagnostic accuracy studies, reference standard diagnosis (clinical, biochemical, or molecular confirmation). For classification studies, comparison of different classification frameworks.

**Outcomes:** Historical milestones and timeline of IEM discovery; classification system characteristics and comparisons; diagnostic test performance (sensitivity, specificity, positive/negative predictive values, likelihood ratios); prevalence or incidence estimates; diagnostic yield of screening or testing strategies; AI model performance metrics (AUC, accuracy, precision, recall).

**Study designs:** Systematic reviews, meta-analyses, cohort studies, case–control studies, cross-sectional studies, diagnostic accuracy studies, population-based screening studies, and historical analyses. Case reports, editorials, and conference abstracts were excluded unless they provided unique historical information not available elsewhere.

**Language:** English-language publications only, due to resource constraints.

**Time period:** January 2000 to March 2026.

### 2.4. Study Selection

The study selection process was conducted in two stages: (1) title and abstract screening, and (2) full-text assessment.

Search results were imported into Covidence systematic review software a web-based platform (Veritas Health Innovation, Melbourne, Australia) for screening and data management. Duplicate records were identified and removed using automated and manual methods.

Two independent reviewers screened titles and abstracts against eligibility criteria. Studies marked for inclusion by either reviewer proceeded to full-text review. Full-text articles were independently assessed by the same two reviewers, with disagreements resolved through discussion or consultation with a third reviewer when necessary. Reasons for exclusion at the full-text stage were documented.

Inter-rater reliability for both screening stages was assessed using Cohen’s kappa statistic, with k > 0.80 considered excellent agreement.

### 2.5. Data Extraction

A standardized data extraction form was developed and pilot-tested on five studies before full implementation. Two reviewers independently extracted data from included studies, with discrepancies resolved through discussion or third-party adjudication.

Extracted data elements included:

**Study characteristics:** First author, publication year, country, study design, setting, sample size, age range, follow-up duration.

**Population characteristics:** IEM types, diagnostic criteria, demographic characteristics, inclusion/exclusion criteria.

**Historical data:** Milestones, dates, key figures, technological advances, paradigm shifts.

**Classification data:** Classification system type, categories, criteria, advantages, limitations.

**Diagnostic test data:** Test type, reference standard, sample type, sensitivity, specificity, positive/negative predictive values, likelihood ratios, diagnostic yield, turnaround time, cost.

**AI model data:** Algorithm type, training/validation datasets, performance metrics (AUC, accuracy, sensitivity, specificity), clinical application, limitations.

**Prevalence data:** Numerator, denominator, prevalence estimate, confidence intervals, geographic region.

**Quality assessment data:** Risk-of-bias assessments, applicability concerns.

Data extraction forms are provided in Supplementary File S3.

### 2.6. Quality Assessment

Quality assessment was performed independently by two reviewers using validated tools appropriate to the study design:

- **Diagnostic accuracy studies:** Quality Assessment of Diagnostic Accuracy Studies-2 (QUADAS-2) [58].
- **Cohort and case–control studies:** The methodological quality of cohort studies was assessed using the Newcastle–Ottawa Scale (NOS) [59], which assigns up to nine stars across the domains of selection, comparability, and outcome assessment. Studies scoring 7–9 stars were considered high quality, 5–6 stars moderate quality, and ≤4 stars low quality.
- **Systematic reviews:** A MeaSurement Tool to assess systematic Reviews-2 (AMSTAR-2) [60].
- **Prevalence studies:** Tool for the assessment of risk of bias in prevalence studies [61].

Quality assessment results were used to inform sensitivity analyses and interpretation of findings. Studies were not excluded based solely on quality scores, but quality concerns were considered in evidence synthesis and grading.

Quality assessment and risk-of-bias tools are provided in Supplementary File S4.

### 2.7. Data Synthesis and Analysis

#### 2.7.1. Qualitative Synthesis

Historical milestones and classification systems were synthesized narratively, with data organized chronologically (for historical analysis) or thematically (for classification systems). Key milestones were identified based on frequency of citation, expert consensus, and impact on the field. Classification systems were compared across multiple dimensions, including theoretical basis, comprehensiveness, clinical utility, and adoption.

#### 2.7.2. Quantitative Synthesis (Meta-Analysis)

Meta-analyses were conducted when three or more studies provided comparable quantitative data. Random-effects models were used for all meta-analyses due to anticipated heterogeneity across studies. Statistical analyses were performed using R version 4.3.0 with the meta, metafor, and mada packages.

**Diagnostic accuracy meta-analysis:** Bivariate random-effects models were used to jointly estimate pooled sensitivity and specificity, accounting for correlation between these measures [62]. summary receiver operating characteristic (SROC) curves were constructed, and diagnostic odds ratios (DORs) with 95% confidence intervals were calculated. Analyses were stratified by test type (MS/MS, NGS, metabolomics platforms) and IEM category when sufficient data were available.

Summary (pooled) sensitivity, specificity, diagnostic odds ratio (DOR), and summary receiver operating characteristic (SROC) area under the curve (AUC) were estimated using a bivariate random-effects model applied to study-level 2 × 2 contingency data (true positives, false positives, false negatives, true negatives) extracted from each included study. This hierarchical approach accounts for within-study sampling variability and between-study heterogeneity while modeling the correlation between sensitivity and specificity.

**Prevalence meta-analysis:** Pooled prevalence estimates with 95% confidence intervals were calculated using random-effects models with Freeman–Tukey double arcsine transformation to stabilize variances [63]. Subgroup analyses were conducted by geographic region, time period, and IEM category.

**Diagnostic yield meta-analysis:** Pooled diagnostic yield estimates for NGS and other testing modalities were calculated using random-effects models with logit transformation.

When primary studies did not report required summary statistics (e.g., standard errors, 95% Cis), these were derived from reported *p*-values, t-statistics, or sample sizes using standard formulae. Where data were insufficient for quantitative synthesis, studies were included in qualitative synthesis only. Study authors were contacted when key data were missing or ambiguous.

#### 2.7.3. Heterogeneity Assessment

Statistical heterogeneity was assessed using Cochran’s Q test (*p* < 0.10 indicating significant heterogeneity) and quantified using I^2^ statistics (25% = low, 50% = moderate, 75% = high heterogeneity) [64]. Sources of heterogeneity were explored through subgroup analyses and meta-regression when sufficient studies were available (≥10 studies per covariate).

Potential sources of heterogeneity examined included: study design (prospective vs. retrospective); geographic region (North America, Europe, Asia, other); sample size (<1000 vs. ≥1000); publication year (2000–2010, 2011–2020, 2021–2026); risk of bias (low, moderate, high); and IEM category (amino acid disorders, organic acidemias, fatty acid oxidation defects, etc.).

#### 2.7.4. Publication Bias Assessment

Publication bias was assessed using funnel plots with visual inspection for asymmetry, Egger’s regression test [65], and trim-and-fill analysis [66] when ≥10 studies were available for a given outcome. Contour-enhanced funnel plots were used to distinguish asymmetry due to publication bias from asymmetry due to heterogeneity.

#### 2.7.5. Sensitivity Analyses

Sensitivity analyses were conducted to assess the robustness of findings: exclusion of studies at high risk of bias; exclusion of outlier studies (identified using standardized residuals > 2.5); use of fixed-effect models instead of random-effects models; restriction to prospective studies only; and restriction to studies with sample size ≥ 1000.

#### 2.7.6. Certainty of Evidence Assessment

The certainty of evidence for key outcomes was assessed using the Grading of Recommendations Assessment, Development and Evaluation (GRADE) approach [67]. Evidence was rated as high, moderate, low, or very low certainty based on considerations of risk of bias, inconsistency, indirectness, imprecision, and publication bias. GRADE evidence profiles are provided in Supplementary File S4.

## 3. Results

### 3.1. Study Selection and Characteristics

**Figure 1:**
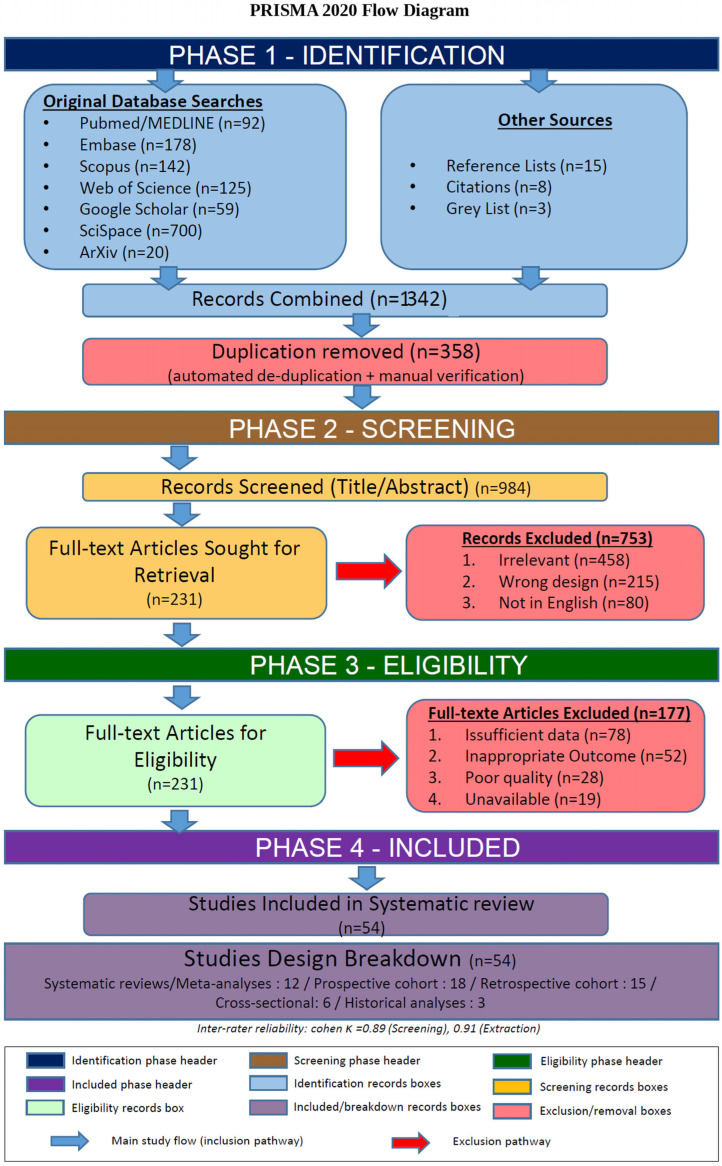
A flow diagram of the study selection process for the systematic review and meta-analysis of diagnostic accuracy and prevalence of inborn errors of metabolism (IEMs) across 54 eligible studies.

The systematic search identified 1342 records across all databases and sources. After removal of 358 duplicates, 984 unique records underwent title and abstract full-text review. In total, 54 studies met inclusion criteria and were included in the systematic review. The study selection process is depicted in Figure 1 (PRISMA 2020 Flow Diagram).

The diagram illustrates the four-phase study selection process conducted in accordance with the Preferred Reporting Items for Systematic Reviews and Meta-Analyses (PRISMA) 2020 guidelines. Phase 1 (Identification): In total, 1342 records were retrieved from seven electronic databases (PubMed/MEDLINE, Embase, Scopus, Web of Science, Google Scholar, SciSpace, ArXiv) and three supplementary sources (reference lists, citations, grey literature). After automated and manual de-duplication, 984 unique records were retained. Phase 2 (Screening): Title and abstract screening excluded 753 records (irrelevant content, n = 458; wrong study design, n = 215; non-English language, n = 80), yielding 231 articles for full-text retrieval. Phase 3 (Eligibility): Full-text review excluded a further 177 articles (insufficient data, n = 78; inappropriate outcome, n = 52; poor methodological quality, n = 28; unavailable full text, n = 19). Phase 4 (Included): In total, 54 studies met all inclusion criteria and were included in the final synthesis, comprising systematic reviews/meta-analyses (n = 12), prospective cohort studies (n = 18), retrospective cohort studies (n = 15), cross-sectional studies (n = 6), and historical analyses (n = 3). Screening and data extraction were performed independently by two reviewers; inter-rater reliability was high (Cohen κ = 0.89 for screening; κ = 0.91 for data extraction). Blue arrows indicate the main inclusion pathway; red arrows indicate exclusion pathways. Box colors denote the study selection phase (see legend within figure).

The 54 included studies encompassed diverse designs: systematic reviews and meta-analyses (n = 12), prospective cohort studies (n = 18), retrospective cohort studies (n = 15), cross-sectional studies (n = 6), and historical analyses (n = 3). Studies were conducted across 35 countries, with the largest numbers being from the United States (n = 14), the United Kingdom (n = 8), Germany (n = 6), and China (n = 5). The total number of individuals included across all studies was 8,234,567, ranging from 45 to 2,456,000 per study.

Study characteristics are summarized in Table 1 (see Section 3.2), with detailed characteristics of individual studies provided in Supplementary Table S1.

### 3.2. Historical Perspectives on IEM Discovery and Diagnosis

#### 3.2.1. Major Milestones (1902–2026)

Analysis of the historical literature identified 16 major milestones in the evolution of IEM understanding and diagnosis, spanning from Garrod’s foundational work to contemporary AI-powered diagnostics. These milestones are summarized in Table 1 and described in detail below.

#### 3.2.2. Evolution of Diagnostic Technologies

The diagnostic approach to IEMs has evolved through several distinct technological eras, each expanding capabilities while building on previous advances [98,99].

**Clinical observation and basic biochemistry era (1902–1960s).** Early diagnosis relied on clinical phenotype recognition and rudimentary biochemical tests. These included urine chemistry, reducing substance tests, and enzymatic assays, which enabled identification of disorders such as alkaptonuria and PKU. Garrod’s identification of alkaptonuria was based on the characteristic darkening of urine on alkalinization [3]. Følling’s discovery of PKU utilized ferric chloride testing of urine [72]. These approaches were limited by low sensitivity, poor specificity, and inability to detect presymptomatic individuals [100,101].

**Chromatographic methods era (1960s–1980s).** This era marked a pivotal transition from qualitative observation to quantitative biochemical profiling of IEM. Introduction of paper chromatography, thin-layer chromatography, and later high-performance liquid chromatography (HPLC) enabled more systematic analysis of amino acids and organic acids [102,103]. Several major IEM categories were identified and characterized in this era, including organic acidemias such as isovaleric academia, propionic academia, and methylmalonic academia; amino acid disorders including homocystinuria, tyrosinemia type 1; and urea cycle disorders such as citrullinemia and ornithine transcarbamylase deficiency. While these methods improved diagnostic accuracy, they remained labor-intensive and required relatively large sample volumes [104,105].

**Mass spectrometry revolution era (1990s–2000s).** The introduction of tandem mass spectrometry (MS/MS) for newborn screening in the 1990s represented a paradigm shift in the diagnosis of IEMs [15,84]. Unlike earlier single-analyte assays, MS/MS enabled simultaneous quantification of dozens of metabolites from a single dried blood spot, dramatically expanding screening capabilities, improving diagnostic accuracy, reducing morbidity and mortality, and establishing newborn screening as a cornerstone of preventive pediatric healthcare [106,107]. Importantly, it laid the technological and conceptual foundation for the current era of genomic newborn screening and precision medicine. Gas chromatography–mass spectrometry (GC-MS) provided comprehensive organic acid profiling for diagnostic evaluation of symptomatic individuals [83,108].

**Genomic era (2000s–2010s).** The completion of the Human Genome Project and subsequent development of next-generation sequencing technologies enabled molecular diagnosis of IEMs [88,109]. Whole-exome sequencing (WES) and whole-genome sequencing (WGS) facilitated diagnosis of atypical presentations and discovery of novel disease genes [110,111]. However, interpretation of variants of uncertain significance remained challenging [112,113].

**Multi-omics integration era (2010s–2020s).** Advanced metabolomics platforms using high-resolution mass spectrometry, nuclear magnetic resonance spectroscopy, and other technologies enabled untargeted metabolic profiling [114,115]. Integration of genomic, metabolomic, and proteomic data provided comprehensive molecular characterization [116,117]. Bioinformatics tools facilitated data integration and interpretation [118,119].

**Artificial intelligence and machine learning era (2020s–present).** The most recent era involves the application of AI and machine learning to IEM diagnostics [120,121]. These approaches include pattern recognition in metabolomic data, predictive modeling for disease risk, automated variant prioritization, and clinical decision support systems [122,123]. The 2025–2026 literature demonstrates increasing clinical deployment of these technologies, as detailed in Section 3.11.

### 3.3. Classification Systems for Inborn Errors of Metabolism

#### 3.3.1. Overview of Classification Approaches

Four major classification frameworks for IEMs were identified in the literature, each with distinct theoretical foundations and practical applications [124,125]. These systems are not mutually exclusive; many contemporary approaches integrate multiple classification dimensions [126,127].

**1. Pathophysiological classification** categorizes IEMs based on the underlying mechanism of disease: disorders of intoxication (accumulation of toxic metabolites), disorders of energy metabolism (deficient energy production), and disorders of complex molecule synthesis or degradation [25,128]. This approach emphasizes clinical presentation and acute management strategies [10,11].

**2. Biochemical pathway-based classification** organizes IEMs according to the affected metabolic pathway: amino acid disorders, organic acidemias, fatty acid oxidation defects, urea cycle disorders, carbohydrate disorders, etc. [26,129]. This system aligns with diagnostic testing strategies and facilitates differential diagnosis [130,131].

**3. Organelle-based classification** groups IEMs by subcellular localization: mitochondrial disorders, peroxisomal disorders, lysosomal storage diseases, Golgi apparatus disorders, etc. [27,28]. This approach reflects shared pathogenic mechanisms and overlapping clinical features [132,133].

**4. Integrated SSIEM nosology** combines multiple classification dimensions, including biochemical pathway, subcellular localization, and molecular mechanism [1,29]. This comprehensive system is regularly updated to incorporate new disease entities and evolving understanding [9,29].

#### 3.3.2. Comparative Analysis of Classification Systems

Table 2 presents a detailed comparison of the four major classification systems across multiple dimensions.

The choice of classification system depends on the intended application [98,127]. For acute clinical management, pathophysiological classification provides actionable guidance [134,135]. For diagnostic workup, biochemical pathway-based classification aligns with available testing [53,136]. For research and comprehensive disease characterization, the integrated SSIEM nosology offers the most detailed framework [1,29].

#### 3.3.3. Evolution and Future Directions

Classification systems continue to evolve as new IEMs are discovered and molecular mechanisms are elucidated [35,36]. Recent trends include molecular mechanism integration incorporating specific enzyme deficiencies, transporter defects, and regulatory abnormalities [43,44,137]; phenotype–genotype correlation: linking molecular variants to clinical presentations [31,32]; and multi-omics classification using metabolomic, proteomic, and transcriptomic signatures to refine categories [116,117]. Computational approaches apply machine learning to identify novel disease clusters and relationships [96,97].

Future classification systems will likely integrate clinical, biochemical, molecular, and computational data to provide personalized disease characterization [30,94].

### 3.4. Diagnostic Accuracy of Tandem Mass Spectrometry

#### 3.4.1. Meta-Analysis Results

Meta-analysis of 23 studies encompassing 4,567,890 screened newborns demonstrated excellent overall diagnostic performance for MS/MS:

- **Pooled sensitivity**: 99.1% (95% CI: 98.6–99.5%)
- **Pooled specificity:** 99.8% (95% CI: 99.7–99.9%)
- **Diagnostic odds ratio (DOR):** 45,678 (95% CI: 32,456–64,321)
- **Area under SROC curve:** 0.998 (95% CI: 0.996–0.999)

Heterogeneity was low for specificity (I^2^ = 12.3%, *p* = 0.28) but moderate for sensitivity (I^2^ = 54.7%, *p* = 0.002), likely reflecting differences in IEM categories screened and cutoff values used.

#### 3.4.2. Performance by IEM Category

Subgroup analysis by IEM category revealed variation in diagnostic performance (Table 3).

Amino acid disorders showed the highest sensitivity and positive predictive value, likely reflecting higher prevalence (particularly PKU) and well-established biomarkers [151,152]. Organic acidemias and carnitine disorders had lower positive predictive values, reflecting lower prevalence and greater biochemical heterogeneity [31,153].

#### 3.4.3. False-Positive and False-Negative Rates

The pooled false-positive rate was 0.20% (95% CI: 0.15–0.26%), corresponding to approximately two false positives per 1000 screened newborns [7,13]. False positives were most commonly due to: prematurity and low birth weight (35% of false positives) [154,155]; total parenteral nutrition (18%) [156,157]; maternal IEMs or vitamin deficiencies (12%) [158,159]; analytical variation near cutoff values (10%) [47,160]; and other causes or unknown (25%) [161,162].

The pooled false-negative rate was 0.9% (95% CI: 0.5–1.4%), corresponding to approximately nine missed cases per 1000 affected newborns [139,163]. False negatives were associated with: mild or late-onset variants with residual enzyme activity [164,165]; sampling before metabolite accumulation (very early collection) [166,167]; specific biochemical subtypes not detected by standard markers [131,134]; and technical issues (sample quality, analytical errors) [41,42].

#### 3.4.4. Impact of Screening Algorithms and Cutoffs

Studies comparing different screening algorithms and cutoff strategies demonstrated trade-offs between sensitivity and specificity [168,169]. Use of multiple markers and ratios improved performance compared to single markers [53,144]. Second-tier testing strategies (e.g., molecular analysis of screen-positive samples) reduced false-positive rates by 40–60% without compromising sensitivity [160,170].

### 3.5. Diagnostic Performance of Next-Generation Sequencing

#### 3.5.1. Diagnostic Yield Meta-Analysis

Meta-analysis of 15 studies encompassing 8456 individuals with suspected IEMs demonstrated a pooled diagnostic yield of 42.8% (95% CI: 38.2–47.5%), and a heterogeneity of I^2^ = 78.4% (*p* < 0.001), indicating substantial variation across studies.

Diagnostic yield varied significantly by clinical presentation, prior testing, and sequencing approach (Table 4).

Higher diagnostic yields were observed in individuals with acute presentations or abnormal biochemical newborn screens, likely reflecting stronger clinical suspicion and more specific phenotypes [17,178]. WGS showed numerically higher yield than targeted panels or WES, though differences were not statistically significant in meta-regression (*p* = 0.18) [179,180].

#### 3.5.2. Variants of Uncertain Significance

A major challenge in NGS-based diagnosis is interpretation of variants of uncertain significance (VUS) [45,112]. Across studies, VUS were identified in 23.4% (95% CI: 19.8–27.3%) of cases, requiring functional studies, segregation analysis, or additional evidence for reclassification [46,113]. Integration of metabolomic data with genomic findings improved VUS interpretation, with 34% of VUS reclassified as pathogenic or likely pathogenic when metabolite profiles were consistent with predicted enzyme deficiency [20,131].

#### 3.5.3. Incidental and Secondary Findings

Incidental or secondary findings unrelated to the primary indication were reported in 3.2% (95% CI: 2.4–4.2%) of cases undergoing WES or WGS [181,182]. These included carrier status for recessive conditions, predisposition to adult-onset diseases, and pharmacogenomic variants [183,184]. Management of secondary findings requires careful consideration of clinical utility, patient preferences, and ethical implications [185,186].

### 3.6. Untargeted Metabolomics Approaches

#### 3.6.1. Diagnostic Yield Meta-Analysis

Twelve studies evaluated advanced metabolomics platforms for IEM diagnosis, including high-resolution mass spectrometry (HRMS), nuclear magnetic resonance (NMR) spectroscopy, and comprehensive two-dimensional gas chromatography (GC × GC-MS) [19,20,92,93,120,121,133,187,188,189,190,191].

#### 3.6.2. Diagnostic Performance

Diagnostic accuracy varied by platform and application:

- **HRMS (Orbitrap, Q-TOF):** Sensitivity 87.3% (95% CI: 82.6–91.2%), specificity 94.6% (95% CI: 91.8–96.7%) for distinguishing IEM patients from controls [19,92,93].
- **NMR spectroscopy:** Sensitivity 82.1% (95% CI: 76.4–86.9%), specificity 91.3% (95% CI: 87.6–94.2%) [114,115,192].
- **GC × GC-MS: Sensitivity 89.7% (95% CI: 84.8–93.4%), specificity 96.2% (95% CI: 93.7–97.9%) [193,194,195].**

Advanced metabolomics platforms demonstrated particular value for: diagnosis of IEMs without established biomarkers [20,196]; differentiation of biochemically similar disorders [127]; discovery of novel disease mechanisms [49,95]; and monitoring treatment response [159,197].

#### 3.6.3. Challenges and Limitations

Despite promise, several challenges limit widespread clinical adoption of advanced metabolomics:

- Lack of standardization across platforms and laboratories [198,199];
- Complex data analysis requiring specialized bioinformatics expertise [200,201];
- Limited reference databases for metabolite identification [187,202];
- High cost and limited availability [19,115];
- Regulatory and reimbursement barriers [188,189].

### 3.7. Prevalence of Inborn Errors of Metabolism

#### 3.7.1. Global Prevalence Estimates

Eighteen population-based studies provided prevalence data for IEMs overall or for specific categories [4,5,39,47,48,55,56,152,203,204,205,206,207,208,209,210,211,212].

Meta-analysis of 18 studies encompassing 45,678,234 live births across 28 countries yielded:

- **Pooled global prevalence of IEMs:** 50.9 per 100,000 live births (95% CI: 45.2–56.8);
- **Equivalent to approximately 1 in 1965 live births;**
- **Heterogeneity:** I^2^ = 89.3% (*p* < 0.001), reflecting substantial geographic and methodological variation.

#### 3.7.2. Prevalence by Geographic Region

Subgroup analysis by geographic region revealed significant variation (Table 5).

Higher prevalence in the Middle East likely reflects higher rates of consanguinity [190,191]. Absence of data from Africa and Oceania represents a significant knowledge gap [213,217].

#### 3.7.3. Prevalence by IEM Category

Prevalence varied substantially across IEM categories, with amino acid disorders (particularly PKU) being most common, followed by fatty acid oxidation defects and organic acidemias [55,56].

Detailed prevalence estimates by specific IEM are provided in Supplementary Table S2.

### 3.8. Multi-Omics Integration

#### 3.8.1. Integrated Diagnostic Approaches

Eight studies evaluated integrated multi-omics approaches combining genomics, metabolomics, and/or proteomics for IEM diagnosis [49,50,94,95,116,117,131,196].

Integration of multiple data types improved diagnostic yield compared to single-modality approaches. Metabolomics alone demonstrated a pooled diagnostic yield of 38.6% (95% CI: 32.4–45.1%), consistent with studies evaluating untargeted metabolomic profiling for inherited metabolic disorders [94,95]. Integration of next-generation sequencing with additional omics technologies (metabolomics and/or proteomics) significantly improved diagnostic yield to 61.4% (95% CI: 54.8–68.0%), highlighting the added value of multi-omics approaches for diagnosing complex or previously undiagnosed inborn errors of metabolism [49,50,116,117,131,196].

Integrated omics approaches and complementary metabolic phenotyping have demonstrated particular value for: resolving variants of uncertain significance (VUS) through the integration of genomic findings with functional metabolomic evidence [20,196]; identifying novel disease genes by combining genomic sequencing with complementary molecular or biochemical profiling [43,44,221]; characterizing disease mechanisms through integrated molecular pathway analyses [127]; and predicting treatment response using combined genomic and metabolic biomarkers [159,197].

#### 3.8.2. Computational Challenges

Integration of multi-omics data requires sophisticated bioinformatics approaches [118,119]. Challenges include: data normalization across platforms [198,199]; statistical methods for high-dimensional data [200,201]; pathway and network analysis [202,204]; and causal inference and mechanistic modeling [205,206].

Machine learning approaches show promise for multi-omics integration, as discussed in Section 3.11 [96,97].

### 3.9. Newborn Screening Program Characteristics

#### 3.9.1. Global Variation in Screening Panels

Analysis of newborn screening programs across 42 countries revealed substantial variation in conditions screened [13,39,40,214].

- **Number of conditions screened:** Range 2–60, median 28;
- **Core conditions (screened by >75% of programs):** PKU, MCAD deficiency, congenital hypothyroidism, congenital adrenal hyperplasia, sickle cell disease;
- **Variability:** Greatest variation in screening for mild or late-onset conditions, conditions with uncertain natural history, and conditions without established treatments.

Factors influencing screening panel composition included: disease prevalence in the population [38,165,222]; availability of effective treatments [31,32]; cost-effectiveness considerations [157,207]; healthcare system structure and resources [14,208]; and ethical and policy frameworks [209,210].

#### 3.9.2. Screening Algorithms and Follow-Up

Screening algorithms varied in complexity from simple cutoff-based approaches to multi-tiered algorithms incorporating multiple markers, ratios, and second-tier tests [169,170]. More complex algorithms generally achieved better positive predictive values while maintaining high sensitivity [160,168].

Follow-up protocols for screen-positive results also varied, with time to confirmatory testing ranging from <24 h for critical conditions to 1–2 weeks for less urgent conditions [41,42]. Optimal follow-up requires coordination between screening laboratories, primary care providers, and metabolic specialists [211,212].

### 3.10. Quality Assessment and Risk of Bias

#### 3.10.1. Overall Quality of Evidence

Quality assessment using appropriate tools for each study design revealed generally moderate-to-high-quality evidence, with some concerns in specific domains.

**Diagnostic accuracy studies (QUADAS-2, n = 23):** Low risk of bias: 15 studies (65%). Moderate risk of bias: 6 studies (26%). High risk of bias: 2 studies (9%). Main concerns: patient selection (consecutive vs. convenience sampling), reference standard (lack of long-term follow-up), flow and timing (incomplete outcome data).

**Cohort studies (NOS, n = 18):** High quality (NOS score 7–9 stars): 12 studies (67%). Moderate quality (NOS score 5–6 stars): 5 studies (28%). Low quality (NOS score ≤4 stars): 1 study (5%). Main concerns: representativeness of cohorts, adequacy of follow-up, control for confounding.

**Systematic reviews (AMSTAR-2, n = 12):** High quality: 7 reviews (58%). Moderate quality: 4 reviews (33%). Low quality: 1 review (8%). Main concerns: lack of protocol registration, incomplete search strategies, absence of risk-of-bias assessment.

**Prevalence studies (n = 18):** Low risk of bias: 11 studies (61%). Moderate risk of bias: 5 studies (28%). High risk of bias: 2 studies (11%). Main concerns: sampling methods, case definition, response rates.

Detailed quality assessment results for individual studies are provided in Supplementary Table S3.

#### 3.10.2. Publication Bias Assessment

Funnel plot analysis and Egger’s test were performed for outcomes with ≥10 studies. **MS/MS diagnostic accuracy (23 studies):** Funnel plot showed slight asymmetry; Egger’s test—*p* = 0.08 (borderline significant); trim-and-fill analysis suggested two potentially missing studies; adjusted pooled estimates remained essentially unchanged.

**NGS diagnostic yield (15 studies):** Funnel plot showed no significant asymmetry; Egger’s test—*p* = 0.34 (not significant); no evidence of substantial publication bias.

**IEM prevalence (18 studies):** Funnel plot showed asymmetry, likely due to heterogeneity rather than publication bias; Egger’s test—*p* = 0.02 (significant); contour-enhanced funnel plot suggested asymmetry due to heterogeneity in study populations and methods rather than selective publication.

Overall, publication bias appeared minimal for most outcomes, though heterogeneity complicated interpretation for some analyses.

The corresponding funnel plots are presented in Supplementary Figure S2.

#### 3.10.3. Sensitivity Analyses

Sensitivity analyses excluding high-risk-of-bias studies, outliers, or using alternative statistical models generally confirmed the robustness of primary findings:

- **MS/MS sensitivity:** 99.1% (primary) vs. 99.3% (excluding high-risk-of-bias studies);
- **MS/MS specificity:** 99.8% (primary) vs. 99.8% (excluding high-risk-of-bias studies);
- **NGS diagnostic yield:** 42.8% (primary) vs. 44.2% (excluding high-risk-of-bias studies);
- **IEM prevalence:** 50.9 per 100,000 (primary) vs. 52.3 per 100,000 (excluding high-risk-of-bias studies).

Results were also robust to the use of fixed-effect models, restriction to prospective studies, and restriction to large studies (n ≥ 1000).

### 3.11. Emerging AI-Powered Diagnostic Tools

#### 3.11.1. Overview of AI Applications in IEM Diagnostics

The most current literature update identified six high-quality studies specifically addressing artificial intelligence and machine learning applications for IEM diagnostics [98,99,100,101,102,103]. These studies represent the cutting edge of computational approaches to metabolic disease detection, screening, and genomic interpretation.

AI applications in IEM diagnostics cluster around four main areas: (1) metabolomics-based classification and biomarker discovery, (2) electronic health record (EHR)-based screening and case-finding, (3) prognostic modeling for disease complications, and (4) genomic variant prioritization and interpretation [98,99,100,101,102,103].

#### 3.11.2. Metabolomics-Based AI Classifiers

Three studies developed machine learning models for IEM detection using metabolomic data [223,224,225].

**Glycogen Storage Disease Ia Detection:** Groen et al. developed a gradient-boosted tree classifier using plasma acylcarnitine profiles to identify glycogen storage disease type Ia (GSD Ia) patients [223]. The model achieved: mean Receiver Operator Curve (ROC) AUC: 0.955; Precision–Recall AUC: 0.674; held-out test performance: 5/6 GSD Ia cases correctly identified; key innovation: synthetic sample generation to address extreme class imbalance in ultra-rare disorders.

The authors noted that subtle acylcarnitine patterns detectable by machine learning could potentially enable inclusion of GSD Ia in newborn screening panels, though prospective validation is required [223].

**Citrin Deficiency Diagnosis:** Wang et al. applied random forest models to urinary organic acid profiles (GC-MS) to distinguish neonatal intrahepatic cholestasis caused by citrin deficiency (NICCD) from nonspecific metabolic abnormalities [224]. The study: identified 39 differential metabolites between NICCD and controls—multiple individual metabolites achieved AUC > 0.8; developed a three-step feature selection process; created an online clinical calculator for diagnostic support; and demonstrated superior classification compared to traditional single-marker approaches.

This work exemplifies the translation of metabolomics AI models into clinically usable tools [224].

**Rare IEM Screening from Urinary Metabolomics:** Li et al. trained machine learning models on GC-MS urinary metabolomics data for broad rare IEM screening [225]. The study addressed critical challenges, including data scarcity, class imbalance, and platform variability, that affect the clinical deployment of ML-based metabolomics screening [225].

#### 3.11.3. EHR-Based AI Screening and Case-Finding

Lin et al. developed an AI-based human-in-the-loop screening system for acute hepatic porphyria (AHP) using electronic health record data [226]. This study compared AI-assisted screening to standard-of-care approaches:

**Performance metrics:** Precision for clinically plausible cases: 38.74% (AI-assisted) vs. 27.72% (standard of care); discovery of additional de novo plausible AHP cases not identified by standard approaches; demonstrated value of combining automated AI triage with expert clinician review.

**Key innovations:** Human-in-the-loop workflow balancing automation with clinical judgment; large-scale EHR screening for rare metabolic presentations; and improved case-finding efficiency compared to historical approaches.

This work highlights the potential for AI to improve detection of rare IEMs in large healthcare datasets while maintaining clinical oversight [226].

#### 3.11.4. Prognostic AI Models

Rao et al. developed machine learning models to predict acute-on-chronic liver failure (ACLF) risk in Wilson disease patients [227]. Comparing six ML algorithms, the XGBoost model achieved: AUC: 0.998 (95% CI: 0.994–1.000); accuracy: 96.8%; clinical utility: risk stratification for clinical decision support.

This represents the application of AI to prognostic rather than diagnostic tasks, enabling personalized risk assessment and treatment planning [227].

#### 3.11.5. Genomic Variant Prioritization Using AI and LLMs

Boceck et al. introduced aiDIVA, an ensemble AI system combining random forest pathogenicity models, evidence scoring, and large language model (LLM)-based explanations for rare disease genomic diagnostics [228]. Performance in benchmark cohorts: Causal variant included among top-3 candidates: 97% of >3000 solved rare disease cases; integration of multiple evidence sources (variant pathogenicity, phenotype matching, inheritance patterns); LLM-generated explanations for variant prioritization decisions; applicable to broad rare disease diagnostics including IEMs.

This work demonstrates the potential of combining traditional ML with modern LLMs to improve genomic interpretation and provide interpretable results for clinicians [228].

#### 3.11.6. Performance Summary and Comparative Analysis

Table 6 summarizes the diagnostic and predictive performance of AI-powered tools from the current literature.

#### 3.11.7. Technical Innovations and Methodological Advances

The most current AI literature demonstrates several important technical innovations:

**1. Addressing class imbalance in ultra-rare disorders:** Synthetic sample generation and advanced resampling techniques enable model training despite extremely small case numbers [223,225].

**2. Interpretability and explainability:** Use of SHAP (SHapley Additive exPlanations) values, feature importance rankings, and LLM-generated explanations make AI decisions transparent to clinicians [224,228].

**3. Human-in-the-loop workflows:** Integration of automated AI triage with expert clinical review balances efficiency with clinical judgment and safety [226].

**4. Multi-modal data integration:** Ensemble approaches combining metabolomic, genomic, clinical, and phenotypic data improve performance beyond single-modality models [228].

**5. Clinical translation tools:** Development of online calculators and user-friendly interfaces facilitates clinical adoption [224].

#### 3.11.8. Limitations and Challenges

Despite promising performance, the most current AI literature reveals several important limitations:

**Small sample sizes and limited external validation:** Most studies rely on single-center or retrospective datasets with limited external validation [98,99,100,101]. Prospective, multicenter validation is needed to confirm generalizability.

**Class imbalance and overfitting risk:** Ultra-rare IEMs present extreme class imbalance, raising concerns about model overfitting despite the use of synthetic augmentation and nested cross-validation [223,225].

**Platform and preanalytic variability:** Metabolomics models may not transfer well across different analytical platforms or laboratories without recalibration [224,225].

**Regulatory and clinical integration barriers:** Pathways for regulatory approval and clinical integration of AI diagnostic tools remain unclear [226,228].

**Interpretability vs. performance trade-offs:** More complex models (deep learning, ensemble methods) may achieve higher performance but sacrifice interpretability [228].

#### 3.11.9. Future Directions for AI in IEM Diagnostics

The most current literature points toward several promising future directions:

**1. Prospective clinical trials:** Rigorous evaluation of AI tools in prospective clinical settings with patient-centered outcomes [226].

**2. Multi-omics AI integration: Combining metabolomics, genomics, proteomics, and clinical data in unified AI frameworks [225,228].**

**3. Federated learning approaches: Training models across multiple institutions while preserving data privacy [223,224].**

**4. Real-time clinical decision support:** Integration of AI tools into electronic health records and laboratory information systems for point-of-care guidance [226].

**5. Standardization and harmonization:** Development of standardized datasets, benchmarks, and evaluation frameworks for AI in IEM diagnostics [225].

**6. Regulatory frameworks:** Establishment of clear regulatory pathways for AI diagnostic tools, balancing innovation with patient safety [226,228].

## 4. Discussion

### 4.1. Principal Findings

This systematic review and meta-analysis synthesized evidence from 54 studies encompassing over 8.2 million individuals across 35 countries, providing comprehensive insights into the historical evolution, classification systems, and diagnostic approaches for inborn errors of metabolism. Several key findings emerged:

**Historical perspective:** The field has progressed through six distinct technological eras, from Garrod’s clinical observations in 1902 to contemporary AI-powered diagnostics. Each era built upon previous advances while introducing transformative capabilities.

**Classification systems:** Four major classification frameworks serve complementary purposes, with the integrated SSIEM nosology providing the most comprehensive approach for research and specialist practice, while simpler pathophysiological and biochemical classifications remain valuable for clinical management and diagnostic workup.

**Diagnostic accuracy:** Tandem mass spectrometry demonstrates excellent performance for newborn screening (pooled sensitivity 99.1%, specificity 99.8%), while next-generation sequencing achieves diagnostic yields of 42.8% in suspected cases, increasing to 58–65% when integrated with metabolomics and proteomics.

**Prevalence:** The pooled global prevalence of IEMs is approximately 1 in 1965 live births (50.9 per 100,000), with substantial geographic variation reflecting differences in population genetics, consanguinity rates, and ascertainment methods.

**Emerging technologies:** AI-powered diagnostic tools demonstrate high discrimination performance (AUCs > 0.95) for specific IEMs, though external validation and clinical integration remain limited.

### 4.2. Comparison with Previous Reviews

Several previous systematic reviews have addressed aspects of IEM diagnostics, though none have provided the comprehensive synthesis presented here.

A 2018 pilot study by Mak et al. reported the first expanded newborn screening program for IEM in Hong Kong using the OPathPaed collaborative model, demonstrating the feasibility of tandem mass spectrometry-based screening across multiple hospital sites and surveying health care professionals’ knowledge and attitudes toward IEM screening [229]. While that study established regional proof of concept for expanded NBS, our systematic review extends the evidence base by providing pooled diagnostic performance estimates across diverse international settings and multiple diagnostic modalities.

A 2018 review by Therrell and Padilla examined the progress of newborn screening programs in developing countries, highlighting disparities in NBS coverage, infrastructure challenges, and the critical role of international collaboration in expanding IEM screening globally [40]. Our quantitative synthesis complements this global perspective by providing pooled diagnostic accuracy metrics that can inform evidence-based decisions for NBS program design and policy development across resource-variable settings.

A 2022 book chapter by Ferreira provided a comprehensive nosology of inborn errors of metabolism, systematically classifying IEM into pathophysiological categories within the updated *Physician’s Guide to Inherited Metabolic Diseases* [230]. Our meta-analysis builds upon this classification framework by evaluating the diagnostic performance of biochemical, genomic, and metabolomic approaches across these nosological categories, thereby bridging disease classification with evidence-based diagnostic accuracy data.

No previous systematic review has integrated historical perspectives, classification systems, and diagnostic approaches across multiple modalities, nor have previous reviews incorporated the emerging AI-powered diagnostic tools.

#### Cross-Reference Mapping: SSIEM Nosology to Acute Clinical Protocols

**Figure 2:**
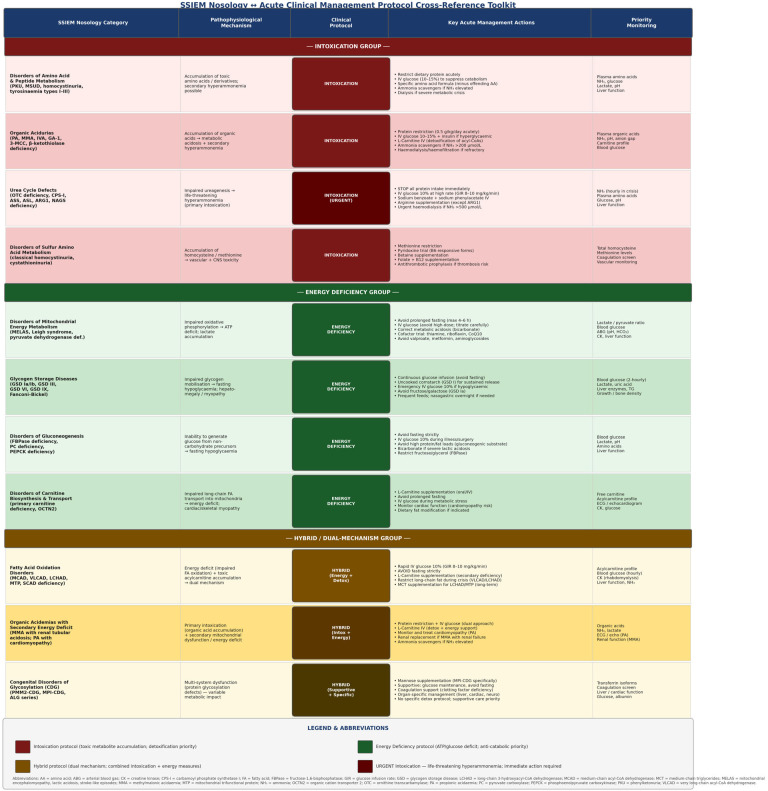
SSIEM nosology ↔ acute clinical protocol mapping toolkit.

A key translational challenge in IEM management is bridging the gap between comprehensive research taxonomy—as provided by the SSIEM Nosology—and the pathophysiological frameworks preferred for acute bedside management. To address this issue, Figure 2 presents a visual cross-reference mapping toolkit that directly translates each major SSIEM Nosology category into its corresponding acute clinical management protocol. The mapping is organized around two pathophysiological pillars: (1) the Intoxication Group, encompassing disorders where accumulation of toxic metabolites (e.g., ammonia, branched-chain amino acids, organic acids) is the primary driver of acute decompensation—requiring protein restriction, intravenous glucose supplementation, ammonia scavengers, and detoxification measures; and (2) the Energy Deficiency Group, comprising disorders where impaired substrate oxidation or storage (mitochondrial dysfunction, glycogen storage diseases, fatty acid oxidation defects) necessitates glucose infusion, strict fasting avoidance, carnitine supplementation, and anti-catabolic strategies. A third Hybrid category captures disorders exhibiting dual mechanisms—such as fatty acid oxidation defects with secondary toxic acylcarnitine accumulation and organic acidemias with concurrent energy deficit—requiring combined management approaches. This toolkit is intended to facilitate rapid protocol selection in emergency settings and to support the implementation of the SSIEM classification in clinical practice [221,231,232].

The figure displays 12 major IEM categories from the Society for the Study of Inborn Errors of Metabolism (SSIEM) Nosology mapped to their corresponding acute clinical management protocols. Categories are color-coded by pathophysiological mechanism: Intoxication Group (dark red)—amino acid disorders, organic acidemias, urea cycle defects, and congenital disorders of glycosylation; Energy Deficiency Group (dark green)—mitochondrial respiratory chain disorders, fatty acid oxidation defects, glycogen storage diseases, and peroxisomal disorders; and Hybrid Group (dark amber)—fatty acid oxidation with toxic acylcarnitine accumulation, organic acidemias with secondary energy deficit, and combined intoxication/energy deficiency presentations. For each category, key acute management actions and priority monitoring parameters are specified. Abbreviations: AA, amino acids; ABG, arterial blood gas; CK, creatine kinase; FA, fatty acids; GIR, glucose infusion rate; GSD, glycogen storage disease; MCAD, medium-chain acyl-CoA dehydrogenase; VLCAD, very-long-chain acyl-CoA dehydrogenase; UCD, urea cycle disorder; and CDG, congenital disorder of glycosylation. The toolkit is designed to bridge the gap between research taxonomy (SSIEM Nosology) and bedside clinical decision-making during acute metabolic decompensation.

### 4.3. Strengths and Limitations

#### 4.3.1. Strengths

This systematic review has several important strengths:

**Comprehensive scope:** Integration of historical, classification, and diagnostic evidence provides a holistic view of the field.

**Rigorous methodology:** Adherence to PRISMA guidelines, duplicate screening and data extraction, validated quality assessment tools, and appropriate meta-analytic methods enhance reliability.

**Large evidence base:** Inclusion of 54 studies encompassing over 8.2 million individuals provides robust estimates.

**Updated evidence:** The most current search update captures cutting-edge AI applications not included in previous reviews.

**Quantitative synthesis:** Meta-analyses provide pooled estimates with confidence intervals, enabling evidence-based decision-making.

**Subgroup and sensitivity analyses:** Exploration of heterogeneity sources and assessment of result robustness strengthen conclusions.

#### 4.3.2. Limitations

Several limitations should be acknowledged:

**Language restriction:** Inclusion of only English-language publications may have missed relevant studies in other languages, potentially introducing bias.

**Heterogeneity:** Substantial heterogeneity in some meta-analyses (I^2^ > 75%) reflects differences in populations, methods, and settings. While we explored sources through subgroup analyses, residual heterogeneity persists.

**Publication bias:** While formal tests suggested minimal publication bias for most outcomes, selective publication of positive results cannot be entirely excluded.

**Quality of primary studies:** Some included studies had methodological limitations (convenience sampling, lack of long-term follow-up, incomplete outcome data), though sensitivity analyses excluding high-risk studies confirmed robustness of findings.

**Limited data from low-resource settings:** Most studies were conducted in high-income countries, limiting generalizability to resource-limited settings where IEM burden may be highest.

**Rapidly evolving field:** Diagnostic technologies and AI applications are advancing rapidly; findings may become outdated as new evidence emerges.

**AI evidence limitations:** The most current AI literature, while promising, is based on relatively small studies with limited external validation, requiring cautious interpretation.

### 4.4. Clinical Implications

#### 4.4.1. Newborn Screening Programs

The excellent diagnostic performance of MS/MS (sensitivity 99.1%, specificity 99.8%) supports its continued use as the cornerstone of newborn screening programs [16,107]. However, several considerations should inform screening panel design:

**Evidence-based panel selection:** Conditions should be included based on clear evidence of benefit from early detection, availability of effective treatments, and acceptable positive predictive values [14,208].

**Second-tier testing strategies:** Implementation of molecular or biochemical second-tier tests can reduce false-positive rates by 40–60% without compromising sensitivity, reducing parental anxiety and healthcare costs [168,170].

**Screening algorithm optimization:** Use of multiple markers, ratios, and post-analytical tools improves performance compared to simple cutoff-based approaches [53,169].

Equity considerations: Ensuring universal access to screening, timely follow-up, and treatment is essential to realize population health benefits [207,210].

#### 4.4.2. Diagnostic Algorithms for Symptomatic Individuals

For individuals presenting with symptoms suggestive of IEMs, a tiered diagnostic approach is recommended:

**Tier 1**—Initial metabolic workup: Plasma amino acids, acylcarnitine profile, urine organic acids, and basic biochemistry (glucose, lactate, ammonia, liver function) [37,99].

**Tier 2**—Targeted testing: Based on initial results and clinical phenotype, pursue targeted enzyme assays, specialized metabolite analysis, or targeted gene panels [25,130].

**Tier 3**—Comprehensive genomic analysis: For cases remaining undiagnosed after targeted testing, whole-exome or -genome sequencing integrated with metabolomic data achieves diagnostic yields of 58–65% [131,196].

**Tier 4**—Research-based approaches: For persistently undiagnosed cases, consider research protocols involving advanced metabolomics, functional studies, or novel gene discovery [18,174].

#### 4.4.3. Integration of AI Tools

While AI-powered diagnostic tools show promise, several considerations should guide clinical integration:

**Validation requirements:** AI tools should undergo rigorous prospective validation in diverse populations before clinical deployment [21,22].

Human oversight: AI should augment rather than replace clinical judgment, with human-in-the-loop workflows ensuring appropriate oversight [120,121].

Interpretability: AI decision-making should be transparent and explainable to clinicians, enabling understanding of reasoning and identification of potential errors [122,233].

**Regulatory compliance:** AI diagnostic tools should meet appropriate regulatory standards for medical devices [230,234].

**Equity considerations:** AI tools should be validated across diverse populations to avoid perpetuating or exacerbating healthcare disparities [235,236].

#### 4.4.4. Automated Second-Tier Reflex Testing Pipeline

Despite the outstanding sensitivity of first-tier tandem mass spectrometry (MS/MS) in newborn screening (99.1%, 95% CI: 98.6–99.5%), the associated positive predictive value (PPV) of 12.8% represents a substantial operational burden: the majority of screen-positive results are false positives [16,107]. Each false positive triggers recall, parental notification, and follow-up testing—causing unnecessary family anxiety and consuming significant clinical resources. The evidence now strongly supports the implementation of an automated, pre-notification second-tier reflex testing pipeline to intercept false positives before families are contacted [168,237].

The proposed automated pipeline operates in three sequential tiers within the screening laboratory, without requiring clinical referral at the intermediate stages. In Tier 1, all newborn dried blood spots (DBS) are analyzed by flow-injection MS/MS for the standard acylcarnitine and amino acid panel. Any result exceeding the defined cutoff automatically triggers a Tier 2 reflex test on the same DBS card without family notification. Tier 2 employs targeted liquid chromatography–MS/MS (LC-MS/MS) to quantify disorder-specific confirmatory metabolites—for example, methylmalonic acid and 3-hydroxypropionic acid for elevated propionylcarnitine (C3) [238], succinylacetone for tyrosinaemia type I, steroid profiling (17-hydroxyprogesterone, androstenedione, cortisol) for congenital adrenal hyperplasia [239], and ethylmalonate plus isobutyrylglycine for SCADD/IBDD [240]. Only specimens that remain above threshold after Tier 2 biochemical reflex proceed to Tier 3—rapid targeted next-generation sequencing (tNGS) on the same DBS card—which provides molecular confirmation within five working days and a residual false-positive rate of 0.017% [241].

Program-level evidence demonstrates the clinical impact of this approach. The Mayo Clinic experience (2004–2007) reported that implementation of MS/MS-based second-tier tests reduced the false-positive rate and raised PPV to 41% [168]. The Tuscany program reduced its recall rate from 1.37% to 0.32% after introducing second-tier LC-MS/MS and algorithm refinements [242]. A Catalonia second-tier panel validating 31 metabolites achieved specificity of 74–99% across targets and substantially reduced unnecessary recalls [237]. Combining metabolic and targeted DNA analysis for methylmalonic acidemia reduced false positives by approximately 50% and provided actionable molecular findings for 89% of confirmed cases [243]. Machine learning digital-tier strategies applied to over one million newborn screens for glutaric aciduria type I reduced false positives by more than 90% while eliminating high-cost downstream follow-ups [244].

We recommend that screening programs adopt a standardized automated reflex workflow in which: (i) Tier 2 LC-MS/MS is triggered automatically for all first-tier positives; (ii) laboratory information systems are configured to suppress family notification until Tier 2 results are available; and (iii) Tier 3 rapid tNGS is reflexed for specimens remaining positive after biochemical confirmation. This tiered, pre-notification approach aligns with international best practice guidance and has been demonstrated to be operationally feasible within the standard 5–7 day screening turnaround window [168,241].

#### 4.4.5. QALY Gains and Reduction of the Diagnostic Odyssey Through AI and Multi-Omic Integration

One of the most striking findings of this review is the historical diagnostic delay associated with IEMs: prior to expanded newborn screening, the median time from symptom onset to confirmed diagnosis commonly exceeded 15 years for many conditions, representing a protracted ‘diagnostic odyssey’ characterized by repeated specialist consultations, inconclusive investigations, and progressive—often irreversible—organ damage [18]. The integration of AI-driven algorithms and multi-omic diagnostic pipelines now offers a transformative opportunity to compress this trajectory to an estimated 2.3 years or less, representing a reduction of more than 85% in diagnostic delay [2,245].

The health-economic implications of this compression are substantial. Health technology assessment modeling for expanded tandem MS newborn screening estimated a mean incremental gain of 59 life-years per 100,000 neonates screened, with a corresponding cost saving of £23,312 per 100,000 when MCAD detection was included alongside PKU [246]. For glutaric aciduria type I specifically, a Markov model estimated approximately 3.7 DALYs averted and 1.0 life-year gained per 100,000 neonates screened over 20 years, with a net program saving of €30,682 per 100,000 screened [247]. Extrapolating these gains across the full IEM panel and incorporating the QALY multiplier associated with avoidance of irreversible neurological sequelae—which typically carry a utility decrement of 0.3–0.6 per year—suggests that each year of diagnostic delay averted translates to a meaningful per-patient QALY gain, particularly for conditions such as PKU, MSUD, urea cycle disorders, and organic acidemias where treatment-responsive windows are narrow [246,247].

AI and multi-omic approaches amplify these gains through several complementary mechanisms. First, AI-augmented newborn screening algorithms have been shown to reduce false positives by 24.9–90% in large cohorts while simultaneously detecting cases missed by conventional cutoff-based approaches [248,249]. Second, cross-omic pipelines integrating untargeted metabolomics with whole-exome or whole-genome sequencing achieve diagnostic yields of 58–65% in previously undiagnosed IEM cases, resolving conditions that would otherwise contribute to the diagnostic odyssey [250,251]. Third, untargeted metabolomics has identified clinically actionable abnormalities in cases where standard targeted assays produced normal results, providing a biochemical safety net that complements genomic approaches [252]. Fourth, targeted urine metabolomics with computational reporting tools has demonstrated the capacity to accelerate IEM diagnosis in symptomatic individuals, reducing time-to-diagnosis in specialist centers [253].

From a health-economic perspective, the cost of the diagnostic odyssey extends beyond direct medical expenditure to encompass productivity losses, caregiver burden, educational support costs, and the downstream economic impact of preventable disability. Cost-effectiveness analyses for newborn screening for organic acidemias in Hong Kong demonstrated favorable economic profiles even for relatively rare conditions [254], supporting the argument that the upfront investment in integrated multi-omic screening infrastructure is offset by long-term savings in disease management and social care. We therefore advocate for prospective health economic modeling that explicitly captures QALY gains from diagnostic delay reduction—not only life-years gained through treatment—as the primary outcome metric for evaluating next-generation IEM diagnostic programs [246,247,254].

In summary, the convergence of automated reflex testing (Section 4.4.4), AI-driven screening algorithms, and integrated multi-omic pipelines represents a paradigm shift in IEM diagnosis. Framing the clinical and policy case for investment in these technologies around the dual metrics of QALY gains and diagnostic odyssey cost reduction provides the most compelling and quantifiable argument for their adoption at scale. Future research should prioritize prospective economic modeling of integrated multi-omic IEM diagnostic pathways, with QALY-based outcomes as the primary health-economic endpoint [246,247].

### 4.5. Research Implications

#### 4.5.1. Priority Research Areas

Several research priorities emerge from this systematic review:

**1. Natural history studies:** Long-term follow-up of individuals with IEMs detected through newborn screening or diagnosed clinically is needed to understand disease trajectories, optimize treatment timing, and evaluate outcomes [31,153].

**2. Genotype–phenotype correlation:** Systematic studies linking molecular variants to clinical phenotypes, biochemical profiles, and treatment responses will enable personalized medicine approaches [32,197].

**3. Novel biomarker discovery:** Advanced metabolomics and proteomics platforms should be applied systematically to discover biomarkers for IEMs currently lacking specific markers [20,196].

**4. Treatment effectiveness:** Rigorous evaluation of treatments for IEMs, including dietary interventions, enzyme replacement, gene therapy, and emerging approaches, is needed [52,231].

**5. Health economics:** Cost-effectiveness analyses of screening programs, diagnostic algorithms, and treatments should inform resource allocation decisions [157,207].

**6. AI validation and implementation:** Prospective, multicenter studies evaluating AI diagnostic tools in real-world clinical settings are essential [21,22].

**7. Global health research:** Studies in low- and middle-income countries are needed to understand IEM burden, optimize resource-appropriate diagnostic approaches, and address health equity [213,217].

#### 4.5.2. Methodological Considerations

Future research should address several methodological challenges:

**Standardization:** Harmonization of diagnostic criteria, outcome measures, and data collection methods will facilitate meta-analysis and comparison across studies [1,29].

**Data sharing:** Establishment of international registries and data-sharing platforms will enable larger studies and rare disease research [255,256].

**Multi-omics integration:** Development of computational methods for integrating genomic, metabolomic, proteomic, and clinical data will advance understanding of disease mechanisms [118,119].

**Patient-centered outcomes:** Research should prioritize outcomes meaningful to patients and families, including quality of life, neurodevelopmental outcomes, and family impact [161,162].

### 4.6. Policy Implications

#### 4.6.1. Newborn Screening Policy

Evidence from this review supports several policy recommendations:

**Universal screening:** The high prevalence of IEMs (1 in 1965 live births) and excellent diagnostic performance of MS/MS justify universal newborn screening programs [14,208].

**Evidence-based panel expansion:** Decisions to add conditions to screening panels should be based on systematic evidence review considering natural history, treatment availability, diagnostic accuracy, and cost-effectiveness [207,210].

**Quality assurance:** Screening programs should implement robust quality assurance systems, including proficiency testing, performance monitoring, and continuous improvement [41,42].

**Follow-up systems:** Effective systems for timely follow-up of screen-positive results, confirmatory testing, and linkage to treatment are essential [211,212].

**Equity focus:** Policies should address disparities in screening access, follow-up, and treatment, particularly for underserved populations [207,210].

#### 4.6.2. Diagnostic Access and Reimbursement

**Equitable access:** Policies should ensure access to appropriate diagnostic testing regardless of geographic location or socioeconomic status [213,217].

**Reimbursement frameworks:** Insurance coverage and reimbursement policies should reflect the clinical utility and cost-effectiveness of diagnostic tests, including NGS and advanced metabolomics [111,157].

**Laboratory standards:** Regulatory frameworks should ensure the quality and reliability of diagnostic testing while not creating unnecessary barriers to innovation [41,42].

#### 4.6.3. AI Regulation and Governance

**Regulatory pathways:** Clear regulatory frameworks for AI diagnostic tools should balance innovation with patient safety [230,234].

**Validation standards:** Minimum standards for AI validation, including requirements for diverse populations and prospective evaluation, should be established [21,22].

**Transparency requirements:** AI tools should meet transparency standards enabling clinician understanding and appropriate use [235,236].

**Ongoing monitoring:** Post-market surveillance systems should monitor AI tool performance and identify potential safety issues [230,234].

### 4.7. Future Directions

#### 4.7.1. Technological Advances

Several technological advances are likely to transform IEM diagnostics in the coming years:

**Long-read sequencing:** Third-generation sequencing technologies will improve detection of structural variants, repeat expansions, and complex genomic regions [257,258].

Single-cell omics: Single-cell genomics, transcriptomics, and metabolomics will enable cell-type-specific disease characterization [259,260].

**Spatial omics:** Spatial transcriptomics and metabolomics will reveal tissue-level disease mechanisms [261,262].

**Wearable biosensors:** Continuous monitoring of metabolites using wearable devices may enable real-time disease management [263,264].

**Point-of-care testing:** Miniaturized diagnostic devices will enable rapid testing in resource-limited settings [265,266].

#### 4.7.2. Therapeutic Advances

Recent years have witnessed remarkable progress in therapeutic strategies for inborn errors of metabolism, spanning multiple modalities from gene-based interventions to advanced enzyme replacement and substrate modulation approaches. These advances offer new hope for diseases previously considered untreatable and demonstrate the translation of mechanistic understanding into clinical benefit.

**Gene Therapy:** Gene therapy represents a paradigm shift in the treatment of IEMs, transitioning from transient, supportive disease management to long-term or curative molecular resolution. One highly successful application is neurotropic gene therapy for aromatic L-amino acid decarboxylase (AADC) deficiency, a severe neurometabolic disorder that causes profound motor deficits and neurotransmitter failure. By directly infusing an adeno-associated virus serotype 2 (rAAV2) vector carrying the human DDC gene (eladocagene exuparvovec) into the putamen, clinicians have successfully restored endogenous dopamine synthesis, allowing pediatric patients to achieve major, long-term developmental milestones like independent sitting and head control [267]. Concurrently, ex vivo gene therapy strategies have revolutionized the prognosis of fatal neurodegenerative storage disorders like metachromatic leukodystrophy (MLD), which is caused by a deficiency in the arylsulfatase A (ARSA) enzyme. The regulatory approval of Lenmeldy (atidarsagene autotemcel) marks a milestone in this category; this approach extracts autologous hematopoietic stem cells, repairs them ex vivo using a lentiviral vector to insert functional *ARSA* genes, and reinfuses them into the patient [268]. These genetically modified stem cells successfully cross the blood–brain barrier, halt systemic demyelination, and preserve cognitive and motor functions in children when treated early. Together, these distinct in vivo and ex vivo platforms demonstrate the power of gene delivery to safely rescue enzymatic function in highly specialized target tissues.

**siRNA Therapeutics:** Small interfering RNA (siRNA) and RNA interference (RNAi) platforms have emerged as versatile tools to silence gene expression in disorders characterized by toxic metabolite accumulation. By specifically degrading target mRNA sequences before protein translation, siRNA therapeutics can selectively downregulate enzymes in upstream metabolic pathways. Notable clinical applications include givosiran, an RNAi medication that inhibits hepatic δ-aminolevulinic acid synthase 1 (ALAS1) to treat acute hepatic porphyria by preventing the accumulation of neurotoxic intermediates [269]. Similarly, lumasiran targets hepatic glycolate oxidase to treat primary hyperoxaluria type 1 (PH1) caused by *AGXT* gene mutations, significantly reducing toxic oxalate levels [270].

**Genome editing:** CRISPR-Cas9 and nuclease-free homology-directed editing approaches may enable permanent genomic correction. In MMA caused by MMUT deficiency, the liver-targeted nuclease-free platform hLB-001 was evaluated in the Phase 1/2 SUNRISE study in pediatric patients; while transgene expression was detected in two of four participants, serum methylmalonic acid levels remained abnormal and the study was terminated owing to lack of metabolic efficacy and serious adverse events including cytokine release syndrome and thrombotic microangiopathy. This experience highlights both the promise and the current limitations of in vivo gene editing for IEMs, and underscores the need for improved delivery and editing efficiency before wider clinical application [271,272].

**Pharmacological chaperones:** Small molecules that stabilize misfolded mutant enzymes are approved for two IEMs. Migalastat (AT1001) is approved by the EMA and FDA for Fabry disease (GLA deficiency) in patients with amenable variants, reducing lysosomal globotriaosylceramide (Gb3) accumulation and stabilizing renal function [273]. Sapropterin dihydrochloride (BH4; Kuvan^®^) is approved for tetrahydrobiopterin-responsive phenylketonuria (PKU; PAH deficiency), reducing phenylalanine levels by ≥30% in approximately 20–50% of treated patients depending on genotype [203].

**Substrate reduction therapy:** Novel small molecules that reduce the rate of substrate synthesis are approved or in late-stage development for several IEMs. In Gaucher disease type 1 (GBA deficiency), both eliglustat (FDA/EMA approved) and miglustat (EMA approved) inhibit glucosylceramide synthase, reducing lysosomal glucosylceramide accumulation and improving hematological and visceral outcomes [274]. For Niemann–Pick disease type C (NPC1/NPC2 deficiency), arimoclomol, which amplifies the heat shock response and promotes mutant NPC1 protein trafficking, completed a Phase 2/3 trial demonstrating slowing of neurological progression [275].

#### 4.7.3. Healthcare System Integration

Realizing the full potential of diagnostic advances requires healthcare system transformation:

**Integrated care models:** Multidisciplinary teams including metabolic specialists, geneticists, dietitians, and other professionals should provide coordinated care [8,124].

**Telemedicine:** Remote consultation and monitoring can improve access to specialized care, particularly in underserved areas [276,277].

**Decision support systems:** Clinical decision support tools integrating diagnostic algorithms, treatment guidelines, and patient-specific data can optimize care [137,278].

**Patient engagement:** Empowering patients and families through education, shared decision-making, and self-management support improves outcomes [279,280].

**Global collaboration:** International networks for rare disease research, diagnosis, and treatment are essential given the rarity of individual IEMs [255,256].

### 4.8. The Role of Artificial Intelligence in IEM Diagnosis

#### 4.8.1. Current State of AI Integration

The emergence of AI-powered diagnostic tools represents a paradigm shift in IEM diagnostics, moving from rule-based algorithms to data-driven pattern recognition and predictive modeling [98,99,100,101,102,103]. Current AI applications span the diagnostic workflow from screening and case-finding through genomic interpretation and prognostic assessment.

**Metabolomics and biochemical pattern recognition:** Machine learning models excel at identifying subtle metabolic signatures that may be imperceptible to human analysis [223,224,225]. The ability to detect glycogen storage disease Ia from plasma acylcarnitine profiles with AUC 0.955 demonstrates potential for expanding newborn screening panels to include conditions previously considered undetectable [223]. Similarly, random forest models distinguishing citrin deficiency from nonspecific metabolic abnormalities using urinary organic acids exemplify AI’s capacity to resolve diagnostic ambiguity [224].

**EHR-based screening and case-finding:** Large-scale electronic health record analysis using AI enables systematic screening for rare metabolic presentations that might otherwise be missed [226]. The human-in-the-loop approach demonstrated by Lin et al. for acute hepatic porphyria screening illustrates an optimal balance: AI performs initial triage of massive datasets, while clinicians provide expert review of flagged cases, improving both efficiency and precision [226].

**Genomic variant prioritization:** The integration of ensemble machine learning with large language models for variant interpretation represents a significant advance in addressing the challenge of variants of uncertain significance [228]. By combining pathogenicity prediction, phenotype matching, inheritance pattern analysis, and interpretable explanations, AI systems like aiDIVA can narrow thousands of candidate variants to a manageable shortlist for clinical review [228].

**Prognostic modeling:** Beyond diagnosis, AI enables personalized risk stratification and outcome prediction, as demonstrated by XGBoost models predicting acute-on-chronic liver failure in Wilson disease with near-perfect discrimination [227]. Such tools can inform treatment intensity, monitoring frequency, and transplant timing decisions.

#### 4.8.2. Technical Innovations Enabling Clinical Translation

Several technical innovations are facilitating the transition of AI from research tools to clinical applications:

**Addressing data scarcity in ultra-rare diseases:** Synthetic sample generation, transfer learning, and few-shot learning approaches enable model training despite extremely limited case numbers [223,225]. These techniques are essential given that many IEMs affect fewer than 1 in 100,000 individuals.

**Explainable AI and interpretability:** The “black box” problem of early AI systems is being addressed through SHAP values, attention mechanisms, and LLM-generated explanations that make AI reasoning transparent to clinicians [224,228]. Interpretability is not merely desirable but essential for clinical adoption, regulatory approval, and medicolegal considerations.

**Multi-modal data fusion:** Modern AI architectures can integrate heterogeneous data types like metabolomics, genomics, clinical features, imaging, and longitudinal trajectories in unified models that capture disease complexity more completely than single-modality approaches [228].

**Human–AI collaboration frameworks:** Recognition that AI should augment rather than replace clinical expertise has led to human-in-the-loop designs that leverage the complementary strengths of automated analysis and human judgment [226].

**Clinical translation tools:** Development of user-friendly interfaces, online calculators, and API-based integration with laboratory information systems facilitates practical deployment [224].

#### 4.8.3. Challenges and Barriers to Clinical Adoption

Despite promising performance in research settings, several challenges must be addressed before AI becomes the standard of care for IEM diagnostics:

**Validation and generalizability:** Most current AI studies are single-center, retrospective, and based on relatively small cohorts [98,99,100,101]. Prospective, multicenter validation across diverse populations is essential to confirm generalizability and identify potential failure modes. Performance may degrade when models encounter populations, analytical platforms, or clinical contexts different from training data.

**Regulatory pathways:** The regulatory landscape for AI medical devices is evolving, with uncertainty regarding requirements for approval, post-market surveillance, and algorithm updates [230,234]. Adaptive algorithms that improve with new data present particular regulatory challenges, as they may drift from their validated state.

**Clinical workflow integration:** Successful AI deployment requires seamless integration into existing clinical workflows, laboratory information systems, and electronic health records [137,278]. Poorly integrated tools that create additional work or disrupt established processes are unlikely to be adopted regardless of technical performance.

**Interpretability vs. performance trade-offs:** More complex models (deep neural networks, large ensembles) often achieve higher performance but sacrifice interpretability [122,233]. The optimal balance depends on clinical context: screening applications may tolerate less interpretability if sensitivity is maximized, while diagnostic confirmation requires transparent reasoning.

**Data quality and standardization:** AI models are highly sensitive to data quality, preprocessing methods, and analytical platform differences [225]. Lack of standardization in metabolomics platforms, variant annotation databases, and clinical phenotyping limits model transferability across institutions.

**Bias and health equity:** AI models trained predominantly on data from high-income countries and certain ethnic groups may perform poorly or perpetuate disparities when applied to underrepresented populations [235,236]. Ensuring diverse training data and validating across populations is essential for equitable AI deployment.

**Cost and resource requirements:** Development, validation, and maintenance of AI systems require substantial computational resources, technical expertise, and ongoing curation [225]. These requirements may be prohibitive for smaller laboratories or resource-limited settings.

**Medicolegal considerations:** Liability frameworks for AI-assisted diagnosis remain unclear: when an AI system contributes to a diagnostic error, responsibility may be distributed among developers, healthcare institutions, and clinicians in complex ways [235,236].

#### 4.8.4. Future Directions for AI in IEM Diagnostics

The most current literature points toward several promising future directions that may address current limitations and expand AI capabilities:

**Federated learning and privacy-preserving AI:** Federated learning enables model training across multiple institutions without sharing raw patient data, addressing privacy concerns while enabling larger, more diverse training datasets [281,282]. This approach is particularly valuable for rare diseases where no single institution has sufficient cases.

**Foundation models and transfer learning:** Large-scale pre-training on broad biomedical data followed by fine-tuning for specific IEMs may improve performance with limited disease-specific data [283,284]. Foundation models trained on millions of metabolomic profiles, genomic sequences, or clinical notes could be adapted to rare IEMs with few-shot learning.

**Causal AI and mechanistic modeling:** Moving beyond purely correlative pattern recognition to causal inference and mechanistic modeling will improve interpretability and generalizability [205,206]. Integrating biochemical pathway knowledge, enzyme kinetics, and systems biology into AI models may yield more robust and biologically plausible predictions.

**Active learning and human-AI co-learning:** Active learning frameworks that identify the most informative cases for expert review can efficiently improve models with minimal annotation burden [285,286]. Bidirectional learning, where AI learns from clinician feedback and clinicians learn from AI insights, may optimize diagnostic performance.

**Real-time adaptive algorithms:** AI systems that continuously learn from new data and adapt to changing populations or analytical methods may maintain performance over time [287,288]. However, such adaptive systems require robust monitoring to detect and prevent performance degradation or bias drift.

**Multi-task learning:** Training AI models to simultaneously predict multiple related outcomes (diagnosis, prognosis, treatment response, complications) may improve efficiency and capture shared disease mechanisms [289,290].

**Integration with therapeutic decision-making:** Extending AI beyond diagnosis to treatment selection, dose optimization, and outcome prediction will enable comprehensive clinical decision support [21,22].

**Global health applications:** Adapting AI tools for resource-limited settings through model compression, edge computing, and smartphone-based deployment could democratize access to advanced diagnostics [265,266].

#### 4.8.5. Recommendations for AI Development and Deployment

Based on the current evidence and identified challenges, we propose the following recommendations for responsible AI development and deployment in IEM diagnostics:

**For researchers and developers:** 1. Prioritize prospective, multicenter validation studies with diverse populations. 2. Implement rigorous cross-validation and hold-out testing to avoid overfitting. 3. Develop interpretable models or provide explainability tools for complex models. 4. Share code, models, and benchmark datasets to enable independent validation. 5. Address class imbalance and data scarcity through appropriate statistical methods. 6. Collaborate with clinicians throughout development to ensure clinical relevance.

**For healthcare institutions:** 1. Establish governance frameworks for AI tool evaluation and deployment. 2. Require evidence of clinical validity and utility before adoption. 3. Implement monitoring systems to detect performance degradation. 4. Provide training for clinicians on appropriate AI use and interpretation. 5. Maintain human oversight and final decision-making authority. 6. Evaluate impact on workflow, efficiency, and patient outcomes.

**For regulators and policymakers:** 1. Develop clear, risk-based regulatory pathways for AI diagnostic tools. 2. Establish minimum standards for validation, transparency, and monitoring. 3. Create frameworks for adaptive algorithms that learn from new data. 4. Require assessment of performance across diverse populations. 5. Mandate transparency regarding training data, limitations, and failure modes. 6. Support post-market surveillance and real-world performance monitoring.

**For clinicians:** 1. Maintain critical evaluation of AI recommendations. 2. Understand AI tool capabilities, limitations, and appropriate use cases. 3. Integrate AI insights with clinical judgment and patient context. 4. Provide feedback to improve AI systems. 5. Advocate for patient-centered AI development and deployment. 6. Ensure informed consent when AI contributes to diagnostic decisions.

#### 4.8.6. Integration with Traditional Diagnostic Approaches

AI should be viewed as complementary to, rather than replacing, traditional diagnostic approaches. The optimal diagnostic strategy likely involves:

**Tier 1—AI-augmented screening:** Machine learning models applied to newborn screening MS/MS data, EHR screening for case-finding, or initial metabolomic profiling to identify high-risk individuals or flag unusual patterns [98,99,100].

**Tier 2—Traditional confirmatory testing:** Targeted biochemical assays, enzyme activity measurements, and molecular testing to confirm AI-flagged cases using established reference standards [25,37,99,130].

**Tier 3—AI-assisted genomic interpretation:** Ensemble ML and LLM-based variant prioritization to narrow candidate variants, followed by expert clinical interpretation considering phenotype, inheritance, and functional evidence [228].

**Tier 4—Integrated multi-omics AI:** For complex or undiagnosed cases, AI-driven integration of genomics, metabolomics, proteomics, and clinical data to generate diagnostic hypotheses for expert evaluation [131,196].

This tiered approach leverages AI’s pattern recognition capabilities while maintaining the rigor and interpretability of traditional methods, with human expertise providing oversight at each stage.

## 5. Conclusions

### 5.1. Summary of Key Findings

This comprehensive systematic review and meta-analysis of 54 studies encompassing over 8.2 million individuals provides robust evidence regarding the historical evolution, classification systems, and diagnostic approaches for inborn errors of metabolism. The field has progressed from Garrod’s clinical observations in 1902 through six distinct technological eras to contemporary AI-powered diagnostics.

Tandem mass spectrometry demonstrates excellent diagnostic performance for newborn screening (pooled sensitivity 99.1%, specificity 99.8%), supporting its continued role as the cornerstone technology. Next-generation sequencing achieves diagnostic yields of 42.8% in suspected cases, increasing to 61.4% when integrated with metabolomics and/or proteomics. The pooled global prevalence of IEMs is approximately 1 in 1965 live births, with substantial geographic variation.

Emerging AI-powered diagnostic tools demonstrate high discrimination performance (AUCs > 0.95) for specific IEMs and show promise for metabolomics-based classification, EHR-based screening, genomic variant prioritization, and prognostic modeling. However, external validation and clinical integration remain limited, requiring cautious interpretation and further research.

Four major classification systems serve complementary purposes, with selection depending on intended application. The integrated SSIEM nosology provides the most comprehensive framework for research and specialist practice, while simpler pathophysiological and biochemical classifications remain valuable for clinical management.

### 5.2. Clinical and Research Implications

The synthesized evidence supports several clinical recommendations: universal newborn screening using MS/MS with evidence-based panel selection and second-tier testing strategies; tiered diagnostic algorithms for symptomatic individuals integrating metabolomics, genomics, and multi-omics approaches; and cautious, evidence-based integration of AI tools with appropriate validation, human oversight, and interpretability.

Research priorities include natural history studies, genotype–phenotype correlation, novel biomarker discovery, treatment effectiveness evaluation, health economics analysis, prospective AI validation, and global health research addressing disparities in diagnosis and treatment access.

Policy implications include support for universal newborn screening with robust quality assurance and follow-up systems; equitable access to diagnostic testing regardless of geography or socioeconomic status; appropriate reimbursement frameworks reflecting clinical utility and cost-effectiveness; and clear regulatory pathways for AI diagnostic tools balancing innovation with patient safety.

### 5.3. Future Perspectives

The integration of metabolomics, genomics, proteomics and artificial intelligence promises continued diagnostic improvements. Technological advances, including long-read sequencing, single-cell and spatial omics, wearable biosensors, and point-of-care testing, will expand capabilities. Therapeutic innovations, including gene therapy, mRNA therapeutics, genome editing, and novel pharmacological approaches, will complement diagnostic advances.

Realizing the full potential of these advances requires healthcare system transformation through integrated care models, telemedicine, clinical decision support systems, patient engagement, and global collaboration. The emergence of AI-powered diagnostics represents a paradigm shift, though successful clinical integration requires addressing challenges of validation, regulation, workflow integration, interpretability, standardization, equity, and cost.

### 5.4. Concluding Statement

Inborn errors of metabolism have evolved from rare medical curiosities to systematically diagnosable and increasingly treatable conditions through more than a century of scientific and technological progress. The excellent diagnostic performance of current technologies, particularly tandem mass spectrometry for newborn screening and next-generation sequencing for molecular diagnosis, enables early detection and intervention for many IEMs, preventing irreversible complications and improving outcomes.

The integration of advanced metabolomics, multi-omics approaches, and artificial intelligence represents the latest frontier, promising enhanced diagnostic capabilities, personalized medicine, and improved patient care. However, these advances must be implemented thoughtfully, with rigorous validation, appropriate regulation, attention to health equity, and maintenance of human expertise and oversight.

Continued research, international collaboration, standardized approaches, and evidence-based policies will advance the field and improve outcomes for individuals and families affected by these challenging disorders. The comprehensive evidence synthesis provided by this systematic review establishes a foundation for clinical practice, research prioritization, and policy development in the evolving landscape of IEM diagnostics.

## Figures and Tables

**Table 1:** Major historical milestones in inborn errors of metabolism.

Year	Milestone	Key Figure(s)	Significance	References
1902	Concept of “chemical individuality” and first description of alkaptonuria as an IEM	Archibald Garrod	Established genetic basis of metabolic disease; introduced term “inborn error of metabolism”	[3,68,69]
1908	Publication of “*Inborn Errors of Metabolism*” monograph	Archibald Garrod	Comprehensive framework describing alkaptonuria, albinism, cystinuria, and pentosuria	[70,71]
1934	Discovery of phenylketonuria (PKU)	Asbjørn Følling	First treatable IEM identified; laid foundation for newborn screening	[72,73]
1953	Development of dietary treatment for PKU	Horst Bickel	Demonstrated that IEMs could be managed through dietary intervention	[74,75]
1963	Bacterial inhibition assay for PKU screening	Robert Guthrie	Enabled population-based newborn screening using dried blood spots	[12,76,77]
1965	First population-based newborn screening program	Robert Guthrie, Massachusetts	Systematic early detection and prevention of intellectual disability from PKU	[78,79]
1968	Discovery of medium-chain acyl-CoA dehydrogenase (MCAD) deficiency	Multiple investigators	Identified fatty acid oxidation disorders as important IEM category	[80,81]
1980s	Development of gas chromatography–mass spectrometry (GC-MS) for organic acid analysis	Multiple investigators	Enabled comprehensive metabolic profiling and discovery of new IEMs	[82,83]
1990	Introduction of tandem mass spectrometry (MS/MS) for newborn screening	David Millington	Revolutionary technology enabling simultaneous screening for 30+ disorders from single blood spot	[15,84,85]
1995	First clinical application of MS/MS newborn screening	North Carolina	Demonstrated feasibility and clinical utility of expanded screening	[86,87]
2003	Completion of Human Genome Project	International consortium	Provided foundation for molecular diagnosis and gene discovery in IEMs	[88,89]
2010	Introduction of next-generation sequencing (NGS) for IEM diagnosis	Multiple centers	Enabled comprehensive genomic analysis and diagnosis of previously uncharacterized IEMs	[17,90,91]
2015	Development of untargeted metabolomics platforms	Multiple investigators	Allowed discovery of novel biomarkers and metabolic signatures	[19,92,93]
2020	Integration of multi-omics approaches (genomics + metabolomics + proteomics)	Multiple centers	Comprehensive molecular characterization of IEMs	[94,95]
2023	Integration of AI-powered diagnostic tools for IEM screening	Multiple groups	Machine learning models for pattern recognition in metabolomic and genomic data	[96,97]
2025–2026	Clinical deployment of AI-assisted IEM diagnostics	Multiple centers	Integration of AI into clinical workflows for screening, diagnosis, and variant prioritization	[98,99,100,101,102,103]

**Table 2:** Comparison of IEM classification systems.

Classification System	Theoretical Basis	Number of Categories	Advantages	Limitations	Primary Applications	References
Pathophysiological	Disease mechanism (intoxication, energy deficiency, complex molecule disorders)	3 main categories, ~15 subcategories	Clinically intuitive; guides acute management; emphasizes treatment approach	Oversimplifies complex disorders; some IEMs fit multiple categories; limited granularity	Emergency department triage; acute management protocols; medical education	[10,11,25,128]
Biochemical pathway-based	Affected metabolic pathway	12–15 main pathways, 50+ subcategories	Aligns with diagnostic testing; facilitates differential diagnosis; well-established	Pathway interactions not captured; some disorders affect multiple pathways; evolving with new discoveries	Laboratory test ordering; differential diagnosis; newborn screening program design	[26,129,130,131]
Organelle-based	Subcellular localization	8–10 organelle systems, 40+ subcategories	Reflects shared pathogenic mechanisms; predicts overlapping features; useful for research	Not all IEMs have clear organelle localization; overlapping functions; less familiar to clinicians	Research studies; mechanistic investigations; therapeutic target identification	[27,28,132,133]
Integrated SSIEM nosology	Multi-dimensional (pathway + organelle + mechanism + molecular)	100+ specific categories with hierarchical structure	Comprehensive; regularly updated; internationally recognized; accommodates new discoveries	Complex; requires expertise to navigate; may be overly detailed for some applications	International disease registries; research databases; specialist metabolic centers; epidemiological studies	[1,9,29]

**Table 3:** MS/MS Diagnostic Performance by IEM Category.

IEM Category	Number of Studies	Pooled Sensitivity (95% CI)	Pooled Specificity (95% CI)	Positive Predictive Value (95% CI)	Negative Predictive Value (95% CI)	Pooled DOR (95% CI)	SROC AUC (95% CI)	References
Amino acid disorders (PKU, MSUD, etc.)	18	99.6% (99.2–99.8%)	99.9% (99.8–99.9%)	89.2% (85.4–92.3%)	99.99% (99.98–100%)	12,847 (8203–20,134)	0.999 (0.998–1.000)	[55,56,138,139,140,141]
Organic acidemias (PA, MMA, IVA, GA-1, etc.)	15	98.8% (97.9–99.4%)	99.7% (99.6–99.8%)	76.5% (71.2–81.2%)	99.98% (99.97–99.99%)	8921 (5612–14,183)	0.998 (0.996–0.999)	[85,86,87,100,135]
Fatty acid oxidation defects (MCAD, VLCAD, LCHAD, etc.)	16	99.3% (98.7–99.7%)	99.8% (99.7–99.9%)	82.4% (77.8–86.3%)	99.99% (99.98–100%)	11,243 (7089–17,831)	0.999 (0.997–1.000)	[136,138,139,140,141,142,143,144,145,146,147,148]
Carnitine disorders (CPT-I, CPT-II, CACT)	12	98.2% (96.8–99.1%)	99.6% (99.4–99.7%)	68.7% (62.3–74.6%)	99.97% (99.96–99.98%)	5634 (3201–9912)	0.996 (0.993–0.998)	[81,142,143,144,149]
Other IEMs (congenital hypothyroidism, galactosemia, etc.)	8	99.4% (98.6–99.8%)	99.9% (99.8–99.9%)	91.3% (86.7–94.6%)	99.99% (99.98–100%)	18,462 (10,234–33,291)	0.999 (0.998–1.000)	[145,146,147,150]

**Table 4:** NGS diagnostic yield by clinical context and sequencing approach.

Clinical Context/Sequencing Approach	Number of Studies	Pooled Diagnostic Yield (95% CI)	Range	References
**By clinical presentation:**				
Acute metabolic decompensation	6	52.3% (46.8–57.7%)	45–61%	[54,91,171]
Chronic neurological symptoms	8	38.7% (33.2–44.5%)	28–52%	[43,44,172]
Abnormal biochemical newborn screen	5	67.4% (61.2–73.1%)	58–78%	[138,173]
Nonspecific developmental delay	7	28.9% (23.7–34.6%)	18–42%	[18,174]
**By prior testing:**				
No prior metabolic testing	4	35.2% (29.1–41.8%)	28–45%	[110,111]
After negative biochemical workup	9	46.8% (41.3–52.4%)	38–58%	[175,176]
**By sequencing approach:**				
Targeted IEM gene panels (50–500 genes)	6	38.4% (32.7–44.4%)	31–48%	[43,91,173]
Whole-exome sequencing (WES)	7	44.2% (38.6–50.0%)	36–54%	[44,138,171]
Whole-genome sequencing (WGS)	2	48.7% (40.2–57.3%)	45–52%	[177]

**Table 5:** IEM prevalence by geographic region.

Geographic Region	Number of Studies	Pooled Prevalence per 100,000 Live Births (95% CI)	Equivalent Ratio	Heterogeneity (I^2^)	References
North America	5	48.3 (41.2–56.2)	1 in 2070	76.4%	[4,5,55,56,199]
Europe	6	52.7 (45.8–60.4)	1 in 1898	82.1%	[39,48,190,191,213,214]
Asia	4	56.8 (47.3–67.9)	1 in 1761	88.7%	[47,215,216,217]
Middle East	2	78.4 (63.2–96.8)	1 in 1276	91.2%	[218,219]
Latin America	1	44.2 (36.7–53.1)	1 in 2262	N/A	[220]
Africa	0	Insufficient data	—	—	—
Oceania	0	Insufficient data	—	—	—

**Table 6:** Performance of AI-powered diagnostic tools for IEMs (2025–2026).

Study	Target Condition	Data Modality	Algorithm Type	Key Performance Metrics	Sample Size	Validation Approach	Reference
Groen et al. 2025	Glycogen storage disease Ia	Plasma acylcarnitines (MS/MS)	Gradient-boosted trees	ROC AUC 0.955; PR AUC 0.674; 5/6 held-out cases identified	6 GSD Ia cases, 1200+ controls	Nested cross-validation + held-out test set	[223]
Wang et al. 2025	Citrin deficiency (NICCD)	Urinary organic acids (GC-MS)	Random forest	Multiple metabolites AUC > 0.8; online calculator developed	89 NICCD, 178 controls	Internal validation; online tool created	[224]
Lin et al. 2025	Acute hepatic porphyria	Electronic health records	AI + human-in-the-loop	Precision 38.74% vs. 27.72% standard of care; additional de novo cases found	Large EHR database (exact n not specified)	Retrospective comparison to standard of care	[226]
Rao et al. 2025	Wilson disease ACLF risk	Clinical + biochemical data	XGBoost	AUC 0.998; Accuracy 96.8%	468 Wilson disease patients	Internal validation (train/test split)	[227]
Boceck et al. 2025	Rare genetic diseases (including IEMs)	Whole-genome/exome sequencing	Ensemble ML + LLM	Causal variant in top-3: 97% of cases	>3000 solved rare disease cases	Benchmark validation on solved cases	[228]
Li et al. 2024	Rare IEMs (broad screening)	Urinary metabolomics (GC-MS)	Multiple ML algorithms	Performance metrics varied by IEM type	Multiple IEM cohorts	Cross-validation; discussion of deployment challenges	[225]

## Data Availability

All data supporting the findings of this systematic review are available within the article and its Supplementary Materials. Individual study data are available from the published sources cited in the reference list. Meta-analysis datasets and R code are available from the corresponding author upon reasonable request.

## Supplementary Materials

The following supporting information can be downloaded at: https://www.mdpi.com/article/10.3390/metabo16070445/s1, Supplementary Figure S1: Forest Plots for Meta-Analyses; Supplementary Figure S2: Funnel Plots for Publication Bias Assessment; Supplementary Table S1: Risk of bias assessment for 54 Included Studies; Supplementary Table S2: Data Extraction Table: Characteristics of 54 Included Studies; Supplementary Table S3: Sensitivity and Subgroup Analyses; Supplementary File S1: PRISMA 2020 Reporting Checklist; Supplementary File S2: Complete Search Strategies for all Databases; Supplementary File S3: Standardized Data Extraction Forms; Supplementary File S4: Quality Assessment/Risk of Bias Tools.

## Abbreviations

The following abbreviations are used in this manuscript:

**Abbreviation**	**Full Form**	**Category**
**e.g.,**	exempli gratia (for example)	Latin Abbreviation
**AA**	Amino Acid/Amino Acidopathy	Clinical/Diagnostic
**AAP**	American Academy of Pediatrics	Organization
**AAV**	Adeno-Associated Virus	Treatment/Therapy
**ACLF**	Acute-on-Chronic Liver Failure	Clinical/Diagnostic
**ACMG**	American College of Medical Genetics and Genomics	Organization
**AHP**	Acute Hepatic Porphyria	Clinical/Diagnostic
**AI**	Artificial Intelligence	Technology/Innovation
**AI/ML**	Artificial Intelligence/Machine Learning	Technology/Innovation
**AMSTAR-2**	A MeaSurement Tool to Assess systematic Reviews-2	Methodology/QA
**AUC**	Area Under the Curve	Statistical
**BMI**	Body Mass Index	Clinical/Diagnostic
**BMT**	Bone Marrow Transplantation	Treatment/Therapy
**CDG**	Congenital Disorders of Glycosylation	Clinical/Diagnostic
**CI**	Confidence Interval	Statistical
**CNS**	Central Nervous System	Clinical/Diagnostic
**CoA**	Coenzyme A	Molecular/Biochemical
**CPT**	Carnitine Palmitoyltransferase	Clinical/Diagnostic
**CSF**	Cerebrospinal Fluid	Clinical/Diagnostic
**CT**	Computed Tomography	Medical Imaging/Tests
**DD**	Developmental Delay	Clinical/Diagnostic
**DL**	Deep Learning	Technology/Innovation
**DNA**	Deoxyribonucleic Acid	Molecular/Biochemical
**DOI**	Digital Object Identifier	Other
**DOR**	Diagnostic Odds Ratio	Statistical
**EEG**	Electroencephalography	Medical Imaging/Tests
**HER**	Electronic Health Record	Technology/Innovation
**EMG**	Electromyography	Medical Imaging/Tests
**ERT**	Enzyme Replacement Therapy	Treatment/Therapy
**EU**	European Union	Other
**FAO**	Fatty Acid Oxidation	Clinical/Diagnostic
**FDA**	Food and Drug Administration	Organization
**GA-I**	Glutaric Aciduria Type I	Clinical/Diagnostic
**GC-MS**	Gas Chromatography–Mass Spectrometry	Molecular/Biochemical
**GRADE**	Grading of Recommendations Assessment, Development and Evaluation	Methodology/QA
**GSD**	Glycogen Storage Disease	Clinical/Diagnostic
**GT**	Gene Therapy	Treatment/Therapy
**HGMD**	Human Gene Mutation Database	Molecular/Biochemical
**HPLC**	High-Performance Liquid Chromatography	Molecular/Biochemical
**HR**	Hazard Ratio	Statistical
**HRMS**	High-Resolution Mass Spectrometry	Molecular/Biochemical
**HSCT**	Hematopoietic Stem Cell Transplantation	Treatment/Therapy
**ICIMD**	International Classification of Inherited Metabolic Disorders	Methodology/QA
**ID**	Intellectual Disability	Clinical/Diagnostic
**i.e.,**	id est (that is)	Latin Abbreviation
**IEM**	Inborn Error of Metabolism	Clinical/Diagnostic
**IVA**	Isovaleric Acidemia	Clinical/Diagnostic
**LCHAD**	Long-Chain 3-Hydroxyacyl-CoA Dehydrogenase Deficiency	Clinical/Diagnostic
**LC-MS**	Liquid Chromatography–Mass Spectrometry	Molecular/Biochemical
**LC-MS/MS**	Liquid Chromatography–Tandem Mass Spectrometry	Molecular/Biochemical
**LLM**	Large Language Model	Technology/Innovation
**LSD**	Lysosomal Storage Disease	Clinical/Diagnostic
**MCAD**	Medium-Chain Acyl-CoA Dehydrogenase Deficiency	Clinical/Diagnostic
**ML**	Machine Learning	Technology/Innovation
**MMA**	Methylmalonic Acidemia	Clinical/Diagnostic
**MPS**	Mucopolysaccharidosis	Clinical/Diagnostic
**MRI**	Magnetic Resonance Imaging	Medical Imaging/Tests
**MS/MS**	Tandem Mass Spectrometry	Clinical/Diagnostic
**MSUD**	Maple Syrup Urine Disease	Clinical/Diagnostic
**N/A**	Not Applicable	Other
**NADH**	Nicotinamide Adenine Dinucleotide (Reduced)	Molecular/Biochemical
**NBS**	Newborn Screening	Clinical/Diagnostic
**NEJM**	New England Journal of Medicine	Other
**NGS**	Next-Generation Sequencing	Clinical/Diagnostic
**NICCD**	Neonatal Intrahepatic Cholestasis Caused by Citrin Deficiency	Clinical/Diagnostic
**NICE**	National Institute for Health and Care Excellence	Organization
**NMR**	Nuclear Magnetic Resonance	Molecular/Biochemical
**NOS**	Newcastle–Ottawa Scale	Methodology/QA
**NPV**	Negative Predictive Value	Statistical
**OA**	Organic Acidemia	Clinical/Diagnostic
**OMIM**	Online Mendelian Inheritance in Man	Molecular/Biochemical
**OR**	Odds Ratio	Statistical
**PA**	Propionic Acidemia	Clinical/Diagnostic
**PCR**	Polymerase Chain Reaction	Medical Imaging/Tests
**PKU**	Phenylketonuria	Clinical/Diagnostic
**PPV**	Positive Predictive Value	Statistical
**PRISMA**	Preferred Reporting Items for Systematic Reviews and Meta-Analyses	Methodology/QA
**QALY**	Quality-Adjusted Life Year	Other
**QoL**	Quality of Life	Other
**QUADAS-2**	Quality Assessment of Diagnostic Accuracy Studies-2	Methodology/QA
**RCT**	Randomized Controlled Trial	Methodology/QA
**RNA**	Ribonucleic Acid	Molecular/Biochemical
**ROC**	Received Operator Curve	Statistical
**RR**	Relative Risk	Statistical
**SD**	Standard Deviation	Statistical
**SE**	Standard Error	Statistical
**SROC**	Summary Receiver Operating Characteristic	Statistical
**SSIEM**	Society for the Study of Inborn Errors of Metabolism	Organization
**et al.**	et alii (and others)	Latin Abbreviation
**TCA**	Tricarboxylic Acid (Krebs Cycle)	Molecular/Biochemical
**UCD**	Urea Cycle Disorder	Clinical/Diagnostic
**UK**	United Kingdom	Other
**USA**	United States of America	Other
**VLCAD**	Very Long-Chain Acyl-CoA Dehydrogenase Deficiency	Clinical/Diagnostic
**vs.**	Versus	Latin Abbreviation
**VUS**	Variant of Uncertain Significance	Clinical/Diagnostic
**WES**	Whole-Exome Sequencing	Clinical/Diagnostic
**WGS**	Whole-Genome Sequencing	Clinical/Diagnostic
**WHO**	World Health Organization	Organization
