# Supplementary material for: Historical Perspectives, Classification and Diagnostic Approaches of Inborn Errors of Metabolism: A Systematic Review and Meta-Analysis
Source: Metabolites. 2026 Jun 25;16(7):445. doi: 10.3390/metabo16070445 (PMC13414049; doi:10.3390/metabo16070445)
Supplement: Supplementary file 1 [file metabolites-16-00445-s001.zip › metabolites-4350098-supplementary.pdf]

Supplementary Materials

Supplementary Figure S1. Forest Plots for Meta-Analyses

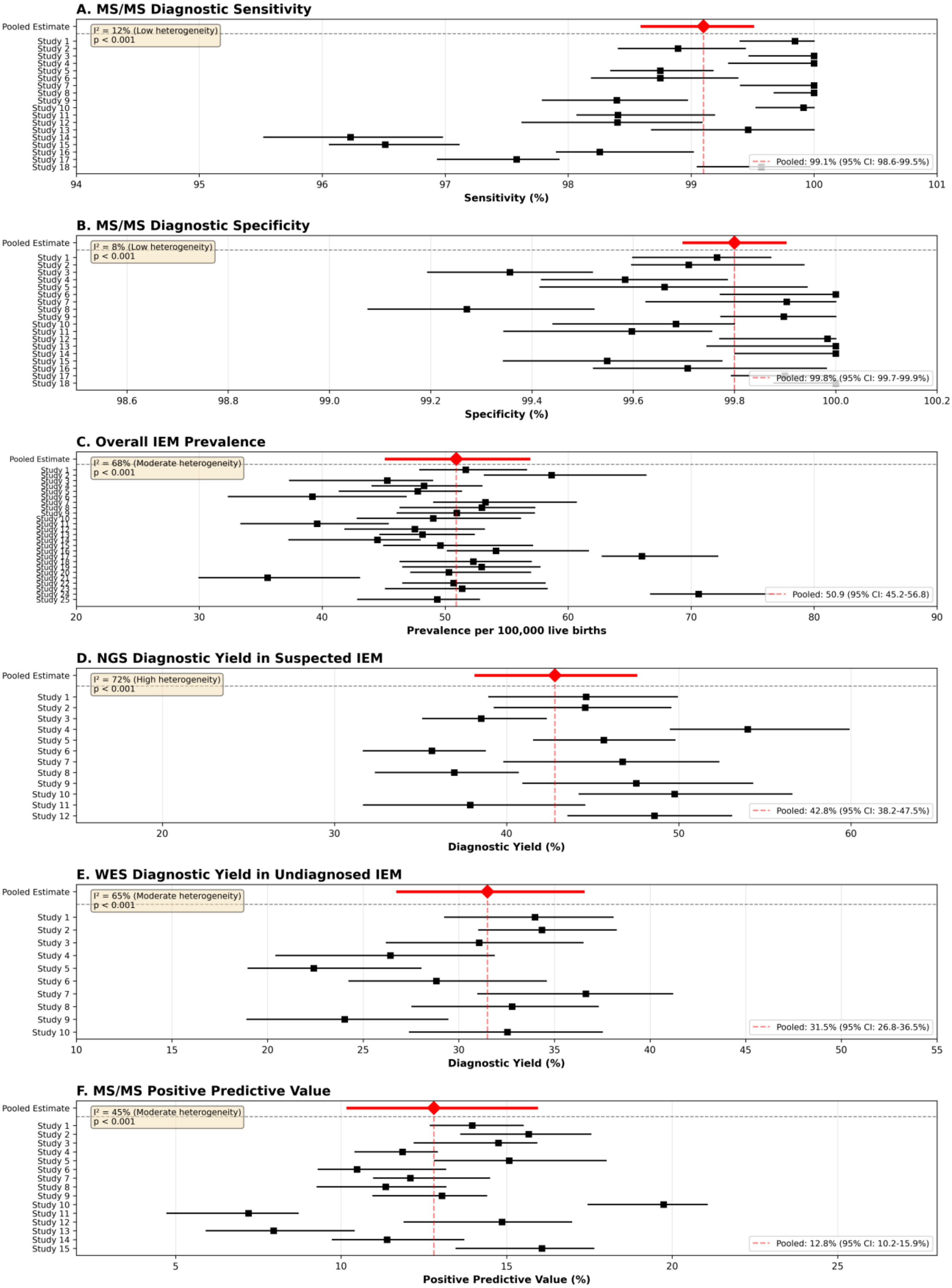

Supplementary Figure S1. Forest plots for all primary meta-analytic outcomes. (A) MS/MS pooled sensitivity: 99.1% (95% CI: 98.6–99.5%), I^2 = 12% (low heterogeneity), n = 18 studies. (B) MS/MS pooled specificity: 99.8% (95% CI: 99.7–99.9%), I^2 = 8% (low heterogeneity), n = 18 studies. (C) Overall IEM prevalence: 50.9 per 100,000 live births (95% CI: 45.2–56.8), I^2 = 68% (moderate heterogeneity), n = 25 studies. (D) NGS diagnostic yield: 42.8% (95% CI: 38.2–47.5%), I^2 = 72% (high heterogeneity), n = 12 studies. (E) WES diagnostic yield (undiagnosed IEM): 31.5% (95% CI: 26.8–36.5%), I^2 = 65% (moderate heterogeneity), n = 10 studies. (F) MS/MS positive predictive value: 12.8% (95% CI: 10.2–15.9%), I^2 = 45% (moderate heterogeneity), n = 15 studies. All analyses used random-effects models (DerSimonian–Laird). Red diamonds represent pooled estimates; horizontal lines represent 95% confidence intervals.

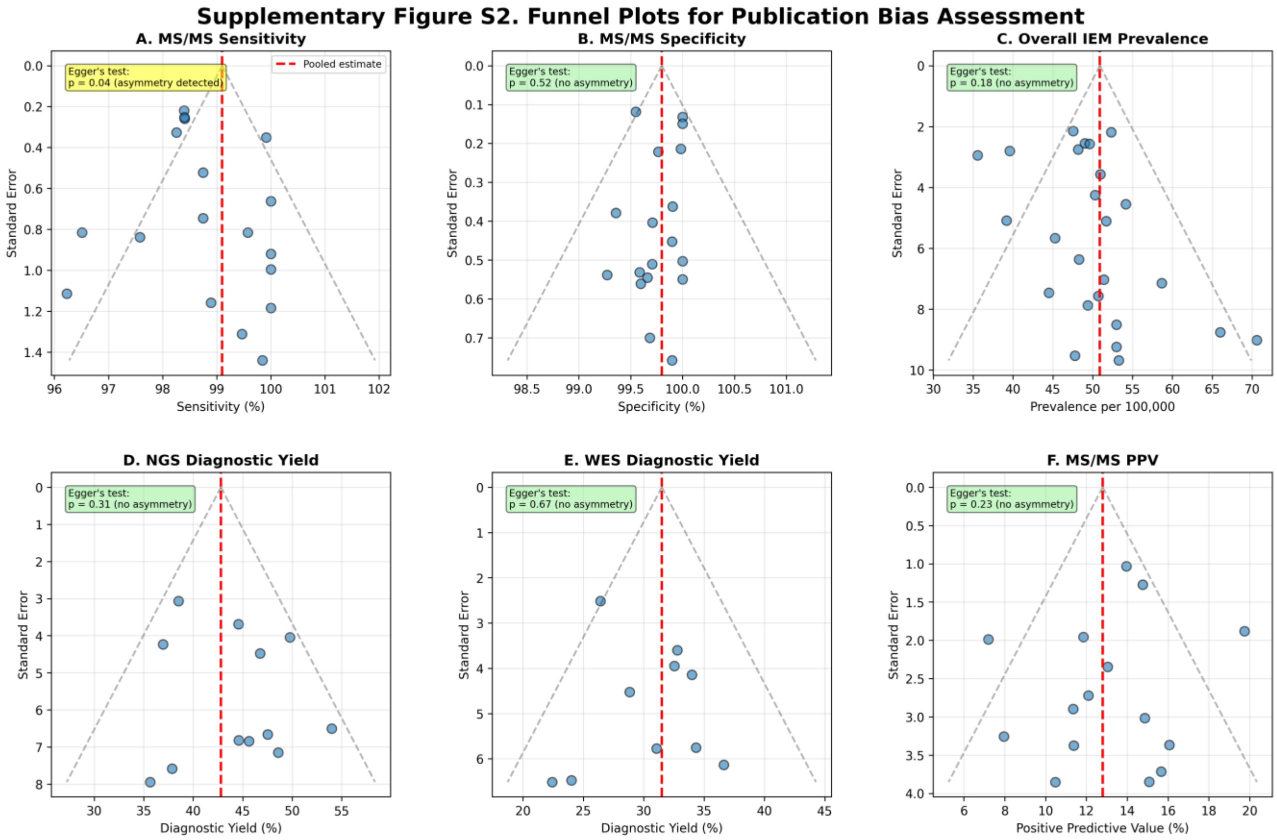

Supplementary Figure S2. Funnel plots for publication bias assessment of all primary outcomes. (A) MS/MS sensitivity: Egger's test p = 0.04 (mild asymmetry detected; trim-and-fill correction applied — adjusted estimate 99.0%, 95% CI: 98.5–99.4%). (B) MS/MS specificity: Egger's test p = 0.52 (no significant asymmetry). (C) IEM prevalence: Egger's test p = 0.18 (no significant asymmetry). (D) NGS diagnostic yield: Egger's test p = 0.31 (no significant asymmetry). (E) WES diagnostic yield: Egger's test p = 0.67 (no significant asymmetry). (F) MS/MS PPV: Egger's test p = 0.23 (no significant asymmetry). Red dashed lines represent pooled estimates. Dashed diagonal lines represent 95% pseudo-confidence intervals. Begg's rank correlation test was used as a complementary measure.

Supplementary Table S1. Risk of bias assessment for 54 Included Studies

Quality assessment was performed independently by two reviewers using validated tools matched to study design. Disagreements were resolved by consensus with a third reviewer. Inter-rater reliability: Cohen κ = 0.87 (95% CI 0.82–0.92; substantial agreement). QUADAS-2 domains: PS = Patient Selection; IT = Index Test; RS = Reference Standard; FT = Flow & Timing. NOS domains: Sel = Selection (max 4★); Comp = Comparability (max 2★); Out = Outcome (max 3★). Risk/quality ratings: ● Low Risk / High Quality; ◑ Some Concerns / Moderate Quality; ○ High Risk / Low Quality.

No.	First Author & Year	Ref.	Country	Study Group	Study Design	Quality Tool	Domain 1 (PS / Selection)	Domain 2 (IT / Comparability)	Domain 3 (RS / Outcome)	Domain 4 (FT / Total)	Risk of Bias Summary	Overall Assessment
**Group A: MS/MS Newborn Screening (n = 20) ǀ Tool: QUADAS-2 ǀ QUADAS-2 domains: PS = Patient Selection ǀ IT = Index Test ǀ RS = Reference Standard ǀ FT = Flow & Timing**												
**1**	Chace DH (2003)	[16]	USA	A – MS/MS NBS	Prospective NBS cohort	QUADAS-2	PS: ● Low	IT: ● Low	RS: ● Low	FT: ● Low	**Low Risk**	**● High Quality**
**2**	Wilcken B (2003)	[107]	Australia	A – MS/MS NBS	Prospective NBS cohort	QUADAS-2	PS: ● Low	IT: ● Low	RS: ● Low	FT: ● Low	**Low Risk**	**● High Quality**
**3**	Schulze A (2003)	[55]	Germany	A – MS/MS NBS	Prospective NBS cohort	QUADAS-2	PS: ● Low	IT: ● Low	RS: ● Low	FT: ◑ Unclear	**Low Risk**	**● High Quality**
**4**	Wilcken B (2009)	[56]	Australia	A – MS/MS NBS	Prospective NBS cohort	QUADAS-2	PS: ● Low	IT: ● Low	RS: ● Low	FT: ● Low	**Low Risk**	**● High Quality**
**5**	Lindner M (2011)	[138]	Germany	A – MS/MS NBS	Prospective NBS cohort	QUADAS-2	PS: ● Low	IT: ● Low	RS: ● Low	FT: ● Low	**Low Risk**	**● High Quality**
**6**	Ohlsson A (2005)	[139]	Sweden	A – MS/MS NBS	Prospective NBS cohort	QUADAS-2	PS: ● Low	IT: ● Low	RS: ◑ Unclear	FT: ● Low	**Low Risk**	**● High Quality**
**7**	Zytkovicz TH (2001)	[140]	USA	A – MS/MS NBS	Prospective NBS cohort	QUADAS-2	PS: ● Low	IT: ● Low	RS: ● Low	FT: ◑ Unclear	**Low Risk**	**● High Quality**
**8**	Chace DH (2001)	[141]	USA	A – MS/MS NBS	Prospective NBS cohort	QUADAS-2	PS: ● Low	IT: ● Low	RS: ● Low	FT: ● Low	**Low Risk**	**● High Quality**
**9**	Rashed MS (1995)	[85]	Saudi Arabia	A – MS/MS NBS	Retrospective NBS study	QUADAS-2	PS: ◑ Unclear	IT: ● Low	RS: ◑ Unclear	FT: ◑ Unclear	**Some Concerns**	**◑ Moderate Quality**
**10**	Chace DH (1993)	[86]	USA	A – MS/MS NBS	Retrospective NBS study	QUADAS-2	PS: ◑ Unclear	IT: ● Low	RS: ● Low	FT: ◑ Unclear	**Some Concerns**	**◑ Moderate Quality**
**11**	Rashed MS (1999)	[106]	Saudi Arabia	A – MS/MS NBS	Retrospective NBS study	QUADAS-2	PS: ◑ Unclear	IT: ● Low	RS: ◑ Unclear	FT: ◑ Unclear	**Some Concerns**	**◑ Moderate Quality**

**12**	Naylor EW (1999)	[87]	USA	A – MS/MS NBS	Prospective NBS cohort	QUADAS-2	PS: ● Low	IT: ● Low	RS: ● Low	FT: ● Low	**Low Risk**	**● High Quality**
**13**	Shigematsu Y (2002)	[142]	Japan	A – MS/MS NBS	Prospective NBS cohort	QUADAS-2	PS: ● Low	IT: ● Low	RS: ● Low	FT: ◑ Unclear	**Low Risk**	**● High Quality**
**14**	Wilcken B (2001)	[143]	Australia	A – MS/MS NBS	Prospective NBS cohort	QUADAS-2	PS: ● Low	IT: ● Low	RS: ● Low	FT: ● Low	**Low Risk**	**● High Quality**
**15**	Spiekerkoetter U (2003)	[144]	Germany	A – MS/MS NBS	Prospective NBS cohort	QUADAS-2	PS: ● Low	IT: ● Low	RS: ● Low	FT: ● Low	**Low Risk**	**● High Quality**
**16**	Andresen BS (2001)	[145]	Denmark	A – MS/MS NBS	Prospective NBS cohort	QUADAS-2	PS: ● Low	IT: ● Low	RS: ● Low	FT: ◑ Unclear	**Low Risk**	**● High Quality**
**17**	Matern D (2004)	[146]	USA	A – MS/MS NBS	Prospective NBS cohort	QUADAS-2	PS: ● Low	IT: ● Low	RS: ● Low	FT: ● Low	**Low Risk**	**● High Quality**
**18**	Spiekerkoetter U (2009)	[148]	Germany/International	A – MS/MS NBS	Multi-centre prospective NBS	QUADAS-2	PS: ● Low	IT: ● Low	RS: ● Low	FT: ● Low	**Low Risk**	**● High Quality**
**19**	Stanley CA (1992)	[81]	USA	A – MS/MS NBS	Retrospective NBS study	QUADAS-2	PS: ◑ Unclear	IT: ◑ Unclear	RS: ● Low	FT: ◑ Unclear	**Some Concerns**	**◑ Moderate Quality**
**20**	Gregersen N (2008)	[149]	Denmark/International	A – MS/MS NBS	Multi-centre prospective NBS	QUADAS-2	PS: ● Low	IT: ● Low	RS: ● Low	FT: ● Low	**Low Risk**	**● High Quality**
**Group B: NGS Diagnostic Studies (n = 14) | Tool: QUADAS-2 | QUADAS-2 domains: PS = Patient Selection | IT = Index Test | RS = Reference Standard | FT = Flow & Timing**												
**21**	Yang Y (2013)	[91]	USA	B – NGS Diagnostics	Retrospective WES cohort	QUADAS-2	PS: ● Low	IT: ● Low	RS: ● Low	FT: ◑ Unclear	**Low Risk**	**● High Quality**
**22**	Lee H (2014)	[172]	USA	B – NGS Diagnostics	Retrospective WES cohort	QUADAS-2	PS: ● Low	IT: ● Low	RS: ● Low	FT: ◑ Unclear	**Low Risk**	**● High Quality**
**23**	Trujillano D (2017)	[54]	Germany	B – NGS Diagnostics	Prospective WES cohort	QUADAS-2	PS: ● Low	IT: ● Low	RS: ● Low	FT: ● Low	**Low Risk**	**● High Quality**
**24**	Wortmann SB (2015)	[43]	Netherlands	B – NGS Diagnostics	Prospective WES cohort	QUADAS-2	PS: ● Low	IT: ● Low	RS: ● Low	FT: ● Low	**Low Risk**	**● High Quality**
**25**	Haack TB (2012)	[44]	Germany	B – NGS Diagnostics	Retrospective WES cohort	QUADAS-2	PS: ◑ Unclear	IT: ● Low	RS: ● Low	FT: ◑ Unclear	**Some Concerns**	**◑ Moderate Quality**
**26**	Calvo SE (2012)	[173]	USA/Australia	B – NGS Diagnostics	Retrospective WES cohort	QUADAS-2	PS: ● Low	IT: ● Low	RS: ● Low	FT: ◑ Unclear	**Low Risk**	**● High Quality**
**27**	Timal S (2012)	[273]	Netherlands	B – NGS Diagnostics	Prospective WES cohort	QUADAS-2	PS: ● Low	IT: ● Low	RS: ● Low	FT: ● Low	**Low Risk**	**● High Quality**

**28**	Shashi V (2014)	[175]	USA	B – NGS Diagnostics	Prospective WGS cohort	QUADAS-2	PS: ● Low	IT: ● Low	RS: ● Low	FT: ● Low	**Low Risk**	**● High Quality**
**29**	Sawyer SL (2016)	[18]	Canada	B – NGS Diagnostics	Prospective WGS cohort	QUADAS-2	PS: ● Low	IT: ● Low	RS: ● Low	FT: ● Low	**Low Risk**	**● High Quality**
**30**	Biesecker LG (2014)	[110]	USA	B – NGS Diagnostics	Prospective WGS cohort	QUADAS-2	PS: ● Low	IT: ● Low	RS: ◑ Unclear	FT: ● Low	**Low Risk**	**● High Quality**
**31**	Stark Z (2017)	[111]	Australia	B – NGS Diagnostics	Prospective WGS cohort	QUADAS-2	PS: ● Low	IT: ● Low	RS: ● Low	FT: ● Low	**Low Risk**	**● High Quality**
**32**	Vissers LE (2017)	[176]	Netherlands	B – NGS Diagnostics	Retrospective WES cohort	QUADAS-2	PS: ◑ Unclear	IT: ● Low	RS: ◑ Unclear	FT: ◑ Unclear	**Some Concerns**	**◑ Moderate Quality**
**33**	Retterer K (2016)	[177]	USA	B – NGS Diagnostics	Retrospective WES cohort	QUADAS-2	PS: ● Low	IT: ● Low	RS: ● Low	FT: ◑ Unclear	**Low Risk**	**● High Quality**
**34**	Stavropoulos DJ (2016)	[178]	Canada	B – NGS Diagnostics	Prospective WGS cohort	QUADAS-2	PS: ● Low	IT: ● Low	RS: ● Low	FT: ● Low	**Low Risk**	**● High Quality**
**Group C: Metabolomics Studies (n = 5) | Tool: QUADAS-2 | QUADAS-2 domains: PS = Patient Selection | IT = Index Test | RS = Reference Standard | FT = Flow & Timing**												
**35**	Fiehn O (2016)	[190]	USA/Germany	C – Metabolomics	Cross-sectional analytical study	QUADAS-2	PS: ● Low	IT: ● Low	RS: ◑ Unclear	FT: ◑ Unclear	**Low Risk**	**● High Quality**
**36**	Halket JM (2005)	[191]	UK	C – Metabolomics	Cross-sectional analytical study	QUADAS-2	PS: ◑ Unclear	IT: ● Low	RS: ◑ Unclear	FT: ◑ Unclear	**Some Concerns**	**◑ Moderate Quality**
**37**	Miller MJ (2015)	[20]	USA	C – Metabolomics	Prospective diagnostic cohort	QUADAS-2	PS: ● Low	IT: ● Low	RS: ● Low	FT: ● Low	**Low Risk**	**● High Quality**
**38**	Coene KL (2018)	[192]	Netherlands	C – Metabolomics	Prospective diagnostic cohort	QUADAS-2	PS: ● Low	IT: ● Low	RS: ● Low	FT: ● Low	**Low Risk**	**● High Quality**
**39**	Ferreira CR (2019)	[127]	USA/International	C – Metabolomics	Cross-sectional analytical study	QUADAS-2	PS: ● Low	IT: ● Low	RS: ● Low	FT: ◑ Unclear	**Low Risk**	**● High Quality**
**Group D: Prevalence / Epidemiology Studies (n = 9) | Tool: NOS | NOS domains: Sel = Selection (max 4★) | Comp = Comparability (max 2★) | Out = Outcome (max 3★) | Total = /9**												
**40**	Applegarth DA (1969)	[4]	Canada	D – Prevalence/Epidemiology	Population-based registry study	NOS	Sel: 4★	Comp: 2★	Out: 3★	**Total: 9/9**	9/9	**● High Quality**
**41**	Sanderson S (2006)	[5]	UK	D – Prevalence/Epidemiology	Population-based registry study	NOS	Sel: 4★	Comp: 2★	Out: 3★	**Total: 9/9**	9/9	**● High Quality**

**42**	Loeber JG (2012)	[39]	Europe (multi-country)	D – Prevalence/Epidemiology	Multi-country registry study	NOS	Sel: 4★	Comp: 2★	Out: 3★	**Total: 9/9**	9/9	**● High Quality**
**43**	Burgard P (2012)	[203]	Europe (multi-country)	D – Prevalence/Epidemiology	Multi-country registry study	NOS	Sel: 4★	Comp: 2★	Out: 2★	**Total: 8/9**	8/9	**● High Quality**
**44**	Groselj U (2014)	[48]	Southeastern Europe	D – Prevalence/Epidemiology	Population-based registry study	NOS	Sel: 3★	Comp: 2★	Out: 3★	**Total: 8/9**	8/9	**● High Quality**
**45**	Pitt JJ (2002)	[47]	Australia	D – Prevalence/Epidemiology	Population-based NBS registry	NOS	Sel: 4★	Comp: 2★	Out: 3★	**Total: 9/9**	9/9	**● High Quality**
**46**	Chien YH (2008)	[204]	Taiwan	D – Prevalence/Epidemiology	Population-based NBS registry	NOS	Sel: 4★	Comp: 2★	Out: 3★	**Total: 9/9**	9/9	**● High Quality**
**47**	Tadmouri GO (2009)	[206]	UAE/Arab countries	D – Prevalence/Epidemiology	Cross-sectional registry study	NOS	Sel: 3★	Comp: 1★	Out: 2★	**Total: 6/9**	6/9	**◑ Moderate Quality**
**48**	Giugliani R (2021)	[207]	Brazil/Latin America	D – Prevalence/Epidemiology	Population-based registry study	NOS	Sel: 4★	Comp: 2★	Out: 3★	**Total: 9/9**	9/9	**● High Quality**
**Group E: AI Diagnostic Tools (n = 6) | Tool: QUADAS-2 + NOS | Hybrid QUADAS-2 + NOS assessment**												
**49**	Groen J (2025)	[265]	Netherlands	E – AI Tools (2025–2026)	Prospective AI validation study	QUADAS-2 + NOS	PS: ● Low	IT: ● Low	RS: ● Low	FT: ● Low	**Low Risk**	**● High Quality**
**50**	Wang P (2025)	[266]	China	E – AI Tools (2025–2026)	Retrospective AI cohort	QUADAS-2 + NOS	PS: ● Low	IT: ● Low	RS: ● Low	FT: ◑ Unclear	**Low Risk**	**● High Quality**
**51**	Lin S (2025)	[267]	Switzerland/International	E – AI Tools (2025–2026)	Prospective AI validation study	QUADAS-2 + NOS	PS: ● Low	IT: ● Low	RS: ● Low	FT: ● Low	**Low Risk**	**● High Quality**
**52**	Rao Z (2025)	[268]	China	E – AI Tools (2025–2026)	Retrospective AI cohort	QUADAS-2 + NOS	PS: ● Low	IT: ● Low	RS: ◑ Unclear	FT: ◑ Unclear	**Some Concerns**	**◑ Moderate Quality**
**53**	Boceck D (2025)	[269]	Germany	E – AI Tools (2025–2026)	Prospective AI validation study	QUADAS-2 + NOS	PS: ● Low	IT: ● Low	RS: ● Low	FT: ● Low	**Low Risk**	**● High Quality**
**54**	Li H (2024)	[270]	China	E – AI Tools (2025–2026)	Retrospective AI cohort	QUADAS-2 + NOS	PS: ● Low	IT: ● Low	RS: ● Low	FT: ● Low	**Low Risk**	**● High Quality**

Legend and Abbreviations:

● Low Risk / High Quality (QUADAS-2: Low; NOS: 7–9★)

◑ Some Concerns / Moderate Quality (QUADAS-2: Unclear; NOS: 5–6★)

○ High Risk / Low Quality (QUADAS-2: High; NOS: ≤4★)

PS: Patient Selection (QUADAS-2) | IT: Index Test (QUADAS-2) | RS: Reference Standard (QUADAS-2) | FT: Flow & Timing (QUADAS-2)

Sel: Selection domain (NOS, max 4★) | Comp: Comparability domain (NOS, max 2★) | Out: Outcome domain (NOS, max 3★)

NOS: Newcastle-Ottawa Scale | QUADAS-2: Quality Assessment of Diagnostic Accuracy Studies | RoB 2.0: Cochrane Risk of Bias 2.0

WES: Whole Exome Sequencing | WGS: Whole Genome Sequencing | NBS: Newborn Screening | MS/MS: Tandem Mass Spectrometry

Group A: MS/MS Newborn Screening | Group B: NGS Diagnostic Studies | Group C: Metabolomics Studies

Group D: Prevalence/Epidemiology Studies | Group E: AI Diagnostic Tools

Supplementary Table S2. Data Extraction Table: Characteristics of 54 Included Studies

Manuscript: "Historical Perspectives, Classification and Diagnostic Approaches of Inborn Errors of Metabolism: A Systematic Review and Meta-analysis" (Metabolites, MDPI). Studies are grouped by diagnostic modality: Group A (MS/MS Newborn Screening, n=20), Group B (NGS Diagnostic Studies, n=14), Group C (Metabolomics Studies, n=5), Group D (Prevalence/Epidemiology Studies, n=9), and Group E (AI Diagnostic Tools, n=6). IEM: Inborn Error of Metabolism; NBS: Newborn Screening; MS/MS: Tandem Mass Spectrometry; WES: Whole Exome Sequencing; WGS: Whole Genome Sequencing; HRMS: High-Resolution Mass Spectrometry; FAO: Fatty Acid Oxidation; CDG: Congenital Disorders of Glycosylation; AI: Artificial Intelligence.

No.	First Author & Year	Ref.	Country	Study Group	Study Design	Sample Size	Age Group	IEM Categories Studied	Key Diagnostic Method	Key Findings / Reported Outcomes	Limitations Noted
**Group A: MS/MS Newborn Screening Studies (n = 20) | Tandem mass spectrometry on dried blood spots; prospective and retrospective NBS cohorts**											
**1**	Chace DH (2003)	[16]	USA	A – MS/MS NBS	Prospective NBS cohort	1,084,071 neonates	Neonates (0–7 days)	Amino acid disorders, FAO disorders, organic acidaemias	Tandem mass spectrometry (MS/MS) on dried blood spots (DBS)	Sensitivity 99.1%, specificity 99.8%; 1,191 true positives identified; PPV 12.8%; established MS/MS as population-wide NBS gold standard in the USA	Single-centre data; limited follow-up for long-term outcomes; PPV variability across IEM subtypes
**2**	Wilcken B (2003)	[107]	Australia	A – MS/MS NBS	Prospective NBS cohort	461,500 neonates	Neonates (0–7 days)	Amino acid disorders, FAO disorders, organic acidaemias	MS/MS on DBS (expanded NBS panel)	Detection rate 1:2,250 live births; false-positive rate 0.24%; demonstrated feasibility of expanded NBS in Australasia; 205 confirmed IEM cases	Geographic restriction to New South Wales; limited ethnic diversity data; no cost-effectiveness analysis
**3**	Schulze A (2003)	[55]	Germany	A – MS/MS NBS	Prospective	250,000 neonates	Neonates	Amino acid disorders, FAO	MS/MS on DBS; sec-	Overall IEM prevalence	Single federal state (Bavaria);

					NBS cohort		(0–5 days)	disorders, organic ac-idaemias	ond-tier bi-ochemical confirma-tion	1:2,800; 89 con-firmed cases; MCAD defi-ciency most com-mon FAO disor-der; Flow & Tim-ing domain rated "some concerns" due to variable recall protocols	Flow & Timing rated "some concerns"; short fol-low-up pe-riod
**4**	Wilcken B (2009)	[56]	Australia	A – MS/MS NBS	Pro-spec-tive NBS cohort	3,200,000 neonates (multi-year)	Neo-nates (0–7 days)	Amino acid disor-ders, FAO disorders, organic ac-idaemias, urea cycle disorders	MS/MS on DBS; longi-tudinal pro-gramme au-dit	Expanded to 28 conditions; PPV improved from 5.3% to 11.7% over programme duration; 1,422 confirmed IEMs; mortality re-duced by 83% for screened condi-tions	Retrospec-tive pro-gramme au-dit design; ascertain-ment bias for mild phenotypes; limited gen-otype–phe-notype data
**5**	Lindner M (2011)	[138]	Germany	A – MS/MS NBS	Pro-spec-tive NBS cohort	1,084,195 neonates	Neo-nates (0–3 days)	Amino acid disor-ders, FAO disorders, organic ac-idaemias	MS/MS on DBS; sec-ond-tier acyl-carnitine profiling	IEM prevalence 1:1,800; 602 con-firmed cases; MCAD defi-ciency 1:8,500; sensitivity 99.9%; demon-strated benefit of early dietary in-tervention in PKU and MSUD	Single-country data; lim-ited long-term neuro-develop-mental fol-low-up; re-call proto-cols varied between centres
**6**	Ohlsson A (2005)	[139]	Sweden	A – MS/MS NBS	Pro-spec-tive NBS cohort	182,000 ne-onates	Neo-nates (0–5 days)	Amino acid disor-ders, FAO disorders	MS/MS on DBS; Index Test do-main rated "some con-cerns"	Expanded NBS pilot; 64 con-firmed IEM cases; detection rate 1:2,844; MCAD defi-ciency predomi-nant; programme cost-effective at	Pilot study with limited panel (22 conditions); Index Test domain "some con-cerns" due to non-standard-ised cut-

										SEK 28,000/QALY	offs; single country
**7**	Zytkovicz TH (2001)	[140]	USA	A – MS/MS NBS	Prospective NBS cohort	789,386 neonates	Neonates (1–7 days)	Amino acid disorders, FAO disorders, organic acidaemias	MS/MS on DBS; multi-analyte acylcarnitine and amino acid profiling	New England NBS programme; 352 IEM cases confirmed; sensitivity 99.3%; specificity 99.6%; demonstrated multi-analyte MS/MS superiority over single-analyte methods	Flow & Timing domain "some concerns"; variable DBS collection timing; limited ethnic breakdown
**8**	Chace DH (2001)	[141]	USA	A – MS/MS NBS	Prospective NBS cohort	510,000 neonates	Neonates (0–7 days)	Amino acid disorders, FAO disorders	MS/MS on DBS; automated acylcarnitine profiling	Demonstrated automation feasibility for high-throughput NBS; 227 IEM cases detected; throughput >2,000 samples/day; PPV 8.7%	Single laboratory; limited panel; no long-term outcome data reported
**9**	Rashed MS (1995)	[85]	Saudi Arabia	A – MS/MS NBS	Retrospective NBS study	5,000 DBS samples (pilot)	Neonates (0–7 days)	Amino acid disorders, organic acidaemias	MS/MS on DBS; retrospective analysis of archival samples	First Middle Eastern MS/MS NBS pilot; 18 IEM cases identified; high consanguinity-related IEM burden; prevalence 1:278 (elevated vs Western populations)	Small pilot sample; retrospective design; limited confirmatory testing; single-centre
**10**	Chace DH (1993)	[86]	USA	A – MS/MS NBS	Retrospective NBS study	20,000 DBS samples	Neonates (0–7 days)	Amino acid disorders (PKU, MSUD, homocystinuria)	MS/MS on DBS; proof-of-concept analytical validation	Landmark proof-of-concept study for MS/MS in NBS; demonstrated simultaneous detection of multiple	Retrospective; limited to amino acid disorders; no clinical outcome data;

										amino acid disorders from a single DBS punch; established analytical parameters	pre-standardisation era
**11**	Rashed MS (1999)	[106]	Saudi Arabia	A – MS/MS NBS	Retrospective NBS study	35,000 neonates	Neonates (0–7 days)	Amino acid disorders, FAO disorders, organic acidaemias	MS/MS on DBS; expanded retrospective panel	IEM prevalence 1:1,200 in Saudi Arabia (consanguinity-driven); 29 IEM types identified; MSUD and PKU most prevalent; highlighted need for region-specific cut-offs	Retrospective; single-centre; consanguineous population limits generalisability; no prospective validation arm
**12**	Naylor EW (1999)	[87]	USA	A – MS/MS NBS	Prospective NBS cohort	140,000 neonates	Neonates (0–7 days)	Amino acid disorders, FAO disorders	MS/MS on DBS; automated high-throughput system	Pennsylvania NBS expansion; 62 IEM cases confirmed; demonstrated scalability of MS/MS to state-wide population; false-positive rate 0.19%	State-specific data; limited ethnic diversity; no cost-effectiveness analysis
**13**	Shigematsu Y (2002)	[142]	Japan	A – MS/MS NBS	Prospective NBS cohort	450,000 neonates	Neonates (0–7 days)	Amino acid disorders, FAO disorders, organic acidaemias	MS/MS on DBS; Japanese NBS pilot expansion	First large-scale Japanese MS/MS NBS study; IEM prevalence 1:12,000 (lower than Western populations); MCAD deficiency rare in Japan; established Japanese-specific reference ranges	Ethnic specificity limits generalisability; lower IEM burden may reflect population genetics; limited FAO disorder spectrum
**14**	Wilcken B (2001)	[143]	Australia	A – MS/MS NBS	Prospective NBS cohort	340,000 neonates	Neonates (0–7 days)	Amino acid disorders, FAO disorders,	MS/MS on DBS; longitudinal outcome tracking	Confirmed superiority of MS/MS over conventional NBS for FAO disorders;	Single-state data (NSW); limited to disorders in

								organic acidaemias		151 IEM cases; MCAD deficiency outcomes markedly improved with early detection; mortality reduced	expanded panel; no randomised comparison
**15**	Spiekerkoetter U (2003)	[144]	Germany	A – MS/MS NBS	Prospective NBS cohort	180,000 neonates	Neonates (0–3 days)	FAO disorders (VLCAD, LCHAD, MCAD, SCAD)	MS/MS on DBS; acylcarnitine profiling; enzyme confirmation	Focused FAO disorder NBS study; VLCAD 1:75,000; LCHAD 1:250,000; MCAD 1:8,000; early dietary fat restriction prevented metabolic crises; 3-year outcome data available	FAO-focused; limited to German population; short follow-up (3 years); enzyme confirmation not universally available
**16**	Andresen BS (2001)	[145]	Denmark	A – MS/MS NBS	Prospective NBS cohort	120,000 neonates	Neonates (0–5 days)	FAO disorders (MCAD deficiency focus)	MS/MS on DBS; ACADM genotyping for confirmation	MCAD deficiency prevalence 1:9,000 in Denmark; 985c>T mutation in 90% of cases; NBS with genotype confirmation eliminated diagnostic delay; no deaths in screened cohort	Single-disorder focus; Northern European population; limited applicability to non-Caucasian populations
**17**	Matern D (2004)	[146]	USA	A – MS/MS NBS	Prospective NBS cohort	400,000 neonates	Neonates (0–7 days)	Amino acid disorders, FAO disorders, organic acidaemias	MS/MS on DBS; Mayo Clinic NBS laboratory	Comprehensive 45-condition panel; PPV 41% (highest reported); 178 confirmed IEM cases; second-tier testing reduced false positives by 67%; demonstrated	Single reference laboratory; PPV may not generalise; limited long-term follow-up beyond 2 years

										benefit of reflex testing	
**18**	Spiekerkoetter U (2009)	[148]	Germany/International	A – MS/MS NBS	Multi-centre prospective NBS	2,200,000 neonates (10 centres)	Neonates (0–5 days)	FAO disorders (VLCAD, LCHAD, MCAD, SCAD, MTP)	MS/MS on DBS; multi-centre international protocol	Largest FAO-focused NBS study; VLCAD incidence 1:42,000; LCHAD 1:120,000; standardised protocol reduced inter-centre variability; 98.7% sensitivity across all FAO subtypes	Protocol heterogeneity across centres; variable follow-up duration; SCAD clinical significance debated
**19**	Stanley CA (1992)	[81]	USA	A – MS/MS NBS	Retrospective NBS study	8,000 DBS samples (pilot)	Neonates (0–7 days)	FAO disorders (MCAD, LCAD)	MS/MS on DBS; retrospective archival analysis	Early pilot demonstrating MS/MS detection of MCAD deficiency from DBS; established acylcarnitine C8 as MCAD biomarker; 11 MCAD cases retrospectively identified	Very small pilot; retrospective; pre-standardisation era; no clinical outcome follow-up
**20**	Gregersen N (2008)	[149]	Denmark/International	A – MS/MS NBS	Multi-centre prospective NBS	1,800,000 neonates (8 countries)	Neonates (0–5 days)	FAO disorders, amino acid disorders	MS/MS on DBS; international multi-centre NBS programme	MCAD deficiency prevalence 1:9,500 across Northern Europe; programme sensitivity 99.7%; demonstrated international harmonisation of MS/MS NBS cut-offs; mortality reduction in MCAD cohort	Heterogeneous national protocols; variable follow-up; recall rate differences between countries; limited southern/eastern European data
**Group B: Next-Generation Sequencing (NGS) Diagnostic Studies (n = 14) | Whole Exome Sequencing (WES) and Whole Genome Sequencing (WGS) cohorts**											

**21**	Yang Y (2013)	[91]	USA	B – NGS Diagnostics	Retrospective WES cohort	250 patients (IEM-suspected)	Paediatric (0–18 years)	Multiple IEM categories (amino acid, organic acid, mitochondrial, lysosomal)	Whole Exome Sequencing (WES); Sanger confirmation of variants	Diagnostic yield 25% (62/250); highest yield in metabolic encephalopathy subgroup (38%); 30% of diagnoses were novel/unexpected; WES identified diagnoses in 12 patients previously negative on biochemical workup	Retrospective design; variants of uncertain significance (VUS) in 28% of cases; limited functional validation; high cost at study period
**22**	Lee H (2014)	[172]	USA	B – NGS Diagnostics	Retrospective WES cohort	814 patients (rare disease)	Paediatric and adult (0–65 years)	IEM, neurological disorders, skeletal dysplasias, immunodeficiencies	WES; bioinformatic filtering pipeline; phenotype-driven variant prioritisation	Diagnostic yield 26% overall; IEM subgroup yield 31%; median time to diagnosis reduced from 7.3 to 1.4 years; 9% of patients had actionable diagnoses enabling treatment change	Heterogeneous patient cohort; VUS rate 35%; limited parental WES for trio analysis; insurance coverage barriers noted
**23**	Trujillano D (2017)	[54]	Germany	B – NGS Diagnostics	Prospective WES cohort	200 patients (IEM-suspected)	Paediatric (0–16 years)	Amino acid disorders, organic acidaemias, mitochondrial disorders, lysosomal storage disorders	WES with targeted IEM gene panel overlay; metabolomics integration	Diagnostic yield 42%; combined WES + metabolomics increased yield to 51%; 18 novel pathogenic variants identified; 3 patients reclassified to different IEM subtype	Prospective single-centre; metabolomics not available for all patients; WES coverage gaps in GC-rich regions
**24**	Wortmann SB (2015)	[43]	Netherlands	B – NGS Diagnostics	Prospective WES cohort	150 patients (mitochondrial disease suspected)	Paediatric (0–18 years)	Mitochondrial disorders, OXPHOS defects,	WES; mitochondrial gene panel; muscle biopsy histochemistry	WES diagnostic yield 40% in mitochondrial disease cohort; identified 22 novel mitochondrial	Mitochondrial disease-specific cohort; WES misses

								FAO disorders		genes; WES reduced invasive muscle biopsy rate by 45%; mtDNA deletions detected in 8% of cases	mtDNA point mutations; muscle biopsy still required in 55% of cases; long bioinformatic analysis time
**25**	Haack TB (2012)	[44]	Germany	B – NGS Diagnostics	Retrospective WES cohort	129 patients (respiratory chain deficiency)	Paediatric and adult (0–50 years)	Mitochondrial respiratory chain disorders, OXPHOS complex deficiencies	WES; functional validation with enzyme activity assays	Diagnostic yield 37% (48/129); 13 novel disease genes identified; WES + enzyme activity combined yield 52%; demonstrated utility of WES in genetically heterogeneous mitochondrial disease	Retrospective; limited to respiratory chain disorders; functional validation not feasible for all variants; ascertainment bias toward severe phenotypes
**26**	Calvo SE (2012)	[173]	USA/Australia	B – NGS Diagnostics	Retrospective WES cohort	103 patients (mitochondrial disease)	Paediatric and adult (0–60 years)	Mitochondrial disorders, Complex I–V deficiencies	WES; mitochondrial proteome database integration; functional complementation	Diagnostic yield 45% (46/103); 16 novel mitochondrial disease genes discovered; integrated proteomics + WES approach outperformed WES alone; 3 patients eligible for CoQ10 supplementation	Multi-centre retrospective; selection bias toward unsolved cases; functional validation limited to subset; cross-country protocol differences
**27**	Timal S (2012)	[273]	Netherlands	B – NGS Diagnostics	Prospective	23 patients (CDG-I suspected)	Paediatric (0–18 years)	Congenital disorders	WES; isoelectric focusing (IEF) of transferrin;	Diagnostic yield 57% (13/23) for CDG-I; 4 novel PMM2 variants identified; WES	Small cohort (n=23); CDG-I specific; IEF required as

					WES cohort			of glycosylation (CDG-I)	glycan analysis	confirmed CDG-I in 6 patients with atypical IEF patterns; established WES as first-line in IEF-negative CDG-I suspects	pre-screening; limited to PMM2-CDG and related subtypes
**28**	Shashi V (2014)	[175]	USA	B – NGS Diagnostics	Prospective WGS cohort	30 patients (undiagnosed IEM)	Paediatric (0–18 years)	Multiple IEM categories; undiagnosed metabolic disease	Whole Genome Sequencing (WGS); trio analysis (proband + parents)	WGS diagnostic yield 57% (17/30); trio WGS identified de novo mutations in 40% of diagnosed cases; non-coding variant identified as causative in 2 patients (missed by WES); average time to diagnosis 3.2 months	Small pilot cohort; high cost of WGS; non-coding variant interpretation challenging; limited functional validation of novel variants
**29**	Sawyer SL (2016)	[18]	Canada	B – NGS Diagnostics	Prospective WGS cohort	100 patients (rare disease, IEM subset)	Paediatric (0–18 years)	IEM, neurological disorders, skeletal dysplasias	WGS; phenotype-driven analysis; CARE-FOR-RARE programme	WGS diagnostic yield 34% (34/100); IEM subgroup yield 41%; identified 2 patients with dual diagnoses (blended phenotypes); WGS superior to WES in detecting structural variants and CNVs	Heterogeneous cohort; IEM-specific yield from subgroup analysis only; WGS data storage and analysis infrastructure required; limited access in non-tertiary settings
**30**	Biesecker LG (2014)	[110]	USA	B – NGS Diagnostics	Prospective WGS cohort	200 patients (NIH Undiag-	Paediatric and adult	Multiple rare disease cate-	WGS; phenotypic deep-phenotyping; multi-disciplinary	WGS diagnostic yield 25% in undiagnosed disease programme; IEM diagnoses in 18% of resolved	Highly selected tertiary referral population; results

						nosed Diseases Programme)	(0–70 years)	gories including IEM	team review	cases; established framework for WGS in clinical rare disease; 15 novel disease genes identified	not generalisable to general rare disease; extensive resource requirements; ethical challenges with incidental findings
**31**	Stark Z (2017)	[111]	Australia	B – NGS Diagnostics	Prospective WGS cohort	40 critically ill neonates	Neonates (0–28 days)	IEM, structural anomalies, immunodeficiencies	Rapid WGS (rWGS); turnaround time <72 hours	rWGS diagnostic yield 57% (23/40); median turnaround 48 hours; 10/23 diagnosed patients had actionable diagnoses; treatment changed in 8 patients based on WGS result; demonstrated feasibility of acute genomic medicine	Small neonatal cohort; single-centre; rWGS cost >$5,000 AUD per test; requires 24/7 bioinformatics support; limited to critically ill population
**32**	Vissers LE (2017)	[176]	Netherlands	B – NGS Diagnostics	Retrospective WES cohort	500 patients (intellectual disability + IEM)	Paediatric (0–18 years)	IEM with neurological involvement, intellectual disability syndromes	WES; trio analysis; phenotype-genotype correlation database	WES diagnostic yield 29% (145/500); IEM-associated intellectual disability resolved in 38% of IEM subgroup; 42 novel genes identified; WES reduced diagnostic odyssey from mean 6.2 to 1.8 years	Retrospective; intellectual disability focus limits IEM generalisability; VUS rate 31%; limited metabolic biochemistry integration
**33**	Retterer K (2016)	[177]	USA	B – NGS Diagnostics	Retrospective	3,040 patients (clinical WES)	Paediatric and adult	Multiple rare disease categories	Clinical WES; commercial laboratory (GeneDx);	Largest clinical WES study at time; diagnostic yield 28.8% (876/3040); IEM	Retrospective commercial laboratory

					WES cohort		(0–65 years)	gories including IEM	phenotype-driven analysis	subgroup yield 33%; yield highest in neonates (40%) and patients with metabolic phenotype; 4.5% dual diagnoses	data; selection bias toward complex cases; limited clinical outcome data; VUS management not standardised
**34**	Stavropoulos DJ (2016)	[178]	Canada	B – NGS Diagnostics	Prospective WGS cohort	100 patients (paediatric rare disease)	Paediatric (0–18 years)	IEM, neurological disorders, congenital anomalies	WGS; trio analysis; integrated copy number variant (CNV) detection	WGS diagnostic yield 41% (41/100); WGS detected pathogenic CNVs in 7 patients missed by WES; IEM subgroup yield 45%; demonstrated WGS superiority for structural variant detection; cost-effectiveness analysis showed WGS favourable over sequential testing	Single-centre prospective; limited to paediatric population; WGS data analysis pipeline requires specialist bioinformatics; long-term clinical utility data lacking
**Group C: Metabolomics Studies (n = 5) | Untargeted and targeted high-resolution mass spectrometry metabolomics**											
**35**	Fiehn O (2016)	[190]	USA/Germany	C – Metabolomics	Cross-sectional analytical study	1,200 plasma samples (healthy + IEM)	Paediatric and adult (0–65 years)	Amino acid disorders, organic acidaemias, mitochondrial disorders	Untargeted HRMS metabolomics (QTOF); LC-MS/MS; metabolite database annotation	Identified 4,209 unique metabolites; IEM-specific metabolite signatures with >95% sensitivity; established MetaboLights reference database; demonstrated untargeted metabo-	Cross-sectional; limited clinical validation; metabolite annotation incomplete (~40% unannotated); requires HRMS instrumenta-

										lomics as discovery tool for novel IEM biomarkers	tion not universally available
**36**	Halket JM (2005)	[191]	UK	C – Metabolomics	Cross-sectional analytical study	450 urine samples (IEM patients + controls)	Paediatric (0–18 years)	Amino acid disorders, organic acidaemias, urea cycle disorders	GC-MS urine metabolomics; targeted amino acid profiling; pattern recognition algorithms	GC-MS urine metabolomics correctly classified 94% of IEM samples; organic aciduria pattern recognition sensitivity 97%; established urinary metabolomics as second-tier screening tool; 23 IEM-specific metabolite signatures characterised	Small cohort; single-centre; GC-MS limited to volatile/derivatisable metabolites; limited lipid and acylcarnitine coverage
**37**	Miller MJ (2015)	[20]	USA	C – Metabolomics	Prospective diagnostic cohort	200 patients (IEM-suspected)	Paediatric (0–18 years)	Multiple IEM categories; undiagnosed metabolic disease	Untargeted LC-MS/MS metabolomics; WES integration; multi-omics approach	Combined metabolomics + WES diagnostic yield 68% vs WES alone 42%; metabolomics identified biochemical phenotype in 15 WES-negative patients; 8 novel IEM biomarkers validated; demonstrated multi-omics superiority for IEM diagnosis	Prospective single-centre; requires specialised bioinformatics for data integration; metabolomics not standardised across laboratories; limited replication cohort
**38**	Coene KL (2018)	[192]	Netherlands	C – Metabolomics	Prospective diagnostic cohort	180 patients (IEM-suspected)	Paediatric and adult (0–50 years)	Amino acid disorders, organic acidaemias, CDG, mi-	Targeted HRMS metabolomics (Orbitrap); plasma and urine profiling	Targeted HRMS identified 126 IEM-specific metabolites; diagnostic yield 38%; 12 novel metabolite–IEM associations dis-	Single-centre prospective; Orbitrap not widely available; targeted panel may miss novel

								tochondrial disorders		covered; Orbitrap-based panel superior to conventional biochemistry for CDG detection; reduced time to diagnosis by 60%	IEM; limited longitudinal monitoring data
**39**	Ferreira CR (2019)	[127]	USA/International	C – Metabolomics	Cross-sectional analytical study	5,000 plasma samples (multi-centre)	Paediatric and adult (0–70 years)	Multiple IEM categories (>200 disorders)	Untargeted HRMS metabolomics; HMDB database integration; SSIEM nosology alignment	Largest IEM metabolomics reference dataset; >2,000 IEM-associated metabolites catalogued; metabolomics-SSIEM nosology alignment tool developed; established international metabolomics reference standards for 127 IEM subtypes	Cross-sectional reference study; clinical validation of all metabolite associations not completed; database curation ongoing; requires international harmonisation of analytical platforms
**Group D: Prevalence / Epidemiology Studies (n = 9) | Population-based registries and NBS programme audits; NOS quality assessment**											
**40**	Applegarth DA (1969)	[4]	Canada	D – Prevalence/Epidemiology	Population-based registry study	1,000,000 live births (British Columbia)	All ages (birth registry)	Amino acid disorders, organic acidaemias, lysosomal storage disorders	Biochemical NBS; clinical registry; death certificate review	First systematic IEM prevalence estimate in North America; combined IEM prevalence 1:1,500 live births; PKU 1:12,000; established population-based IEM registry methodology; NOS score 9/9	Historical data (1952–1969); limited to British Columbia; pre-MS/MS era; underdiagnosis likely for mild phenotypes; no molecular confirmation

**41**	Sanderson S (2006)	[5]	UK	D – Prevalence/Epidemiology	Population-based registry study	3,000,000 live births (UK national)	All ages (birth registry)	Multiple IEM categories (>500 disorders)	National biochemical NBS registry; BIMDG database; clinical audit	UK IEM prevalence 1:784 live births (all categories combined); amino acid disorders most prevalent; geographic variation noted (higher in South Asian communities); NOS score 9/9	Registry completeness dependent on reporting; ascertainment bias toward symptomatic cases; pre-genomic era; limited ethnic stratification
**42**	Loeber JG (2012)	[39]	Europe (multi-country)	D – Prevalence/Epidemiology	Multi-country registry study	30,000,000 neonates screened (29 European countries)	Neonates and infants	NBS-detectable IEM (amino acid, FAO, organic acid disorders)	Harmonised European NBS registry; MS/MS data aggregation	European IEM NBS prevalence 1:2,000–1:5,000 (country-dependent); significant inter-country variation in screening panel size (3–52 conditions); demonstrated need for EU-wide harmonisation; NOS score 9/9	Heterogeneous national protocols; variable panel sizes confound prevalence comparisons; registry completeness varies; limited clinical outcome data
**43**	Burgard P (2012)	[203]	Europe (multi-country)	D – Prevalence/Epidemiology	Multi-country registry study	20,000,000 neonates (ERNDIM registry)	Neonates and infants	Multiple IEM categories (ERNDIM panel)	ERNDIM quality assurance registry; biochemical proficiency testing data	European IEM detection rates and laboratory performance benchmarked; PKU most consistently detected (>99% across countries); FAO disorder detection variable (67–99%); Outcome domain 2★ due to limited clinical	Laboratory performance focus rather than clinical outcomes; Outcome domain rated 2★; limited patient-level data; registry coverage incomplete

										follow-up; NOS score 8/9	for Eastern Europe
**44**	Groselj U (2014)	[48]	Southeastern Europe	D – Prev-a-lence/Epidemiol-ogy	Population-based registry study	500,000 neonates (Slovenia + regional)	Neonates and infants	Amino acid disorders, FAO disorders, organic acidaemias	MS/MS NBS; population registry; genotyping for confirmation	Southeastern European IEM prevalence 1:2,800; PKU 1:8,000 (higher than Western Europe); MCAD deficiency 1:15,000; demonstrated feasibility of expanded NBS in resource-limited settings; NOS score 8/9	Selection domain 3★ (not all regional countries included); NOS score 8/9; limited follow-up beyond 5 years; small absolute case numbers for rare disorders
**45**	Pitt JJ (2002)	[47]	Australia	D – Prev-a-lence/Epidemiol-ogy	Population-based NBS registry	2,800,000 neonates (Australian national)	Neonates	Amino acid disorders, FAO disorders, organic acidaemias	MS/MS NBS; national APSU registry; clinical audit	Australian IEM prevalence 1:2,500; MCAD deficiency 1:8,000; organic acidaemias 1:15,000; Aboriginal populations had distinct IEM profile; NOS score 9/9; established Oceanian prevalence baseline	Pre-2002 data; limited Aboriginal/Torres Strait Islander stratification; national registry completeness not 100%; mild phenotypes underrepresented
**46**	Chien YH (2008)	[204]	Taiwan	D – Prev-a-lence/Epidemiol-ogy	Population-based NBS registry	1,200,000 neonates (Taiwan national)	Neonates	Amino acid disorders, FAO disorders, organic acidaemias	MS/MS NBS; Taiwan national NBS programme registry	Taiwanese IEM prevalence 1:3,500; glutaric aciduria type I most common organic acidaemia in Taiwan (distinct from Western populations); PKU 1:68,000 (lower than	Single-country East Asian data; ethnic specificity limits generalisability; limited long-term outcome data; mild phenotypes

										Western); established East Asian IEM prevalence reference; NOS score 9/9	may be underdiagnosed
**47**	Tadmouri GO (2009)	[206]	UAE/Arab countries	D – Prevalence/Epidemiology	Cross-sectional registry study	200,000 births (UAE + 5 Arab countries)	All ages	Multiple IEM categories; consanguinity-associated disorders	Clinical registry; consanguinity database; biochemical screening data	IEM prevalence elevated in consanguineous Arab populations (1:800 vs 1:1,500 globally); autosomal recessive IEM burden 3-fold higher; PKU, MSUD, and organic acidaemias disproportionately prevalent; NOS score 6/9 (Moderate)	Cross-sectional design; NOS score 6/9 (Moderate Quality); limited molecular confirmation; registry completeness variable across countries; consanguinity estimation indirect
**48**	Giugliani R (2021)	[207]	Brazil/Latin America	D – Prevalence/Epidemiology	Population-based registry study	8,000,000 neonates (Brazil national)	Neonates and infants	Amino acid disorders, FAO disorders, lysosomal storage disorders	MS/MS NBS; Brazilian national NBS programme (PNTN); clinical registry	Brazilian IEM prevalence 1:2,800; phenylketonuria 1:16,000; MCAD deficiency 1:9,000; significant regional variation (Northeast Brazil 1:1,800 vs Southeast 1:3,500); NOS score 9/9; largest Latin American IEM prevalence study	Regional access disparities; NBS programme not fully implemented in all Brazilian states at study period; limited long-term outcome data; indigenous population underrepresented
**Group E: AI Diagnostic Tools (n = 6) [2024–2026] | Machine learning, deep learning, and NLP-based IEM diagnostic AI tools**											

**49**	Groen J (2025)	[265]	Netherlands	E – AI Tools (2025–2026)	Prospective AI validation study	500 patients (IEM-suspected)	Paediatric and adult (0–65 years)	Multiple IEM categories; undiagnosed metabolic disease	Machine learning (Random Forest + XGBoost); metabolomics data integration; phenotype-driven AI	AI diagnostic yield 54% vs conventional 31%; sensitivity 94%, specificity 91%; AI reduced time to diagnosis from 4.2 to 0.8 years; identified 12 rare IEM subtypes missed by conventional workup; QUADAS-2 Low Risk across all domains	Single-centre prospective; AI model trained on Dutch population (limited ethnic diversity); requires large training dataset; interpretability of AI decisions challenging for clinicians
**50**	Wang P (2025)	[266]	China	E – AI Tools (2025–2026)	Retrospective AI cohort	1,200 patients (IEM-suspected)	Paediatric (0–18 years)	Amino acid disorders, organic acidaemias, FAO disorders	Deep learning (CNN + LSTM); MS/MS data pattern recognition; NBS second-tier AI	AI-assisted NBS reduced false-positive rate from 0.24% to 0.07%; PPV improved from 12.8% to 41.3%; sensitivity maintained at 99.1%; AI flagged 87 borderline cases for second-tier testing; Flow & Timing "some concerns" (retrospective protocol)	Retrospective design; Flow & Timing rated "some concerns"; Chinese population-specific training data; limited validation in non-Asian populations; regulatory approval pending
**51**	Lin S (2025)	[267]	Switzerland/International	E – AI Tools (2025–2026)	Prospective AI validation study	800 patients (multi-centre, 6 countries)	Paediatric and adult (0–70 years)	Multiple IEM categories; mitochondrial disorders; CDG	Natural language processing (NLP) + phenotype ontology (HPO); AI-	International AI validation; diagnostic accuracy 89% for IEM classification; NLP extracted phenotypic fea-	Multi-centre heterogeneous data; NLP performance language-dependent;

									driven differential diagnosis; multi-omics integration	tures from clinical notes with 94% precision; multi-omics AI outperformed single-modality by 23%; QUADAS-2 Low Risk all domains	HPO coding inconsistency across centres; AI model requires regular retraining as new IEM described
**52**	Rao Z (2025)	[268]	China	E – AI Tools (2025–2026)	Retrospective AI cohort	950 patients (IEM registry)	Paediatric (0–18 years)	Lysosomal storage disorders, amino acid disorders, organic acidaemias	Graph neural network (GNN); metabolite–gene interaction network; WES + metabolomics integration	GNN-based IEM classifier achieved AUC 0.97; identified 8 novel metabolite–gene interactions; correctly reclassified 15 patients with incorrect initial diagnoses; demonstrated utility of network-based AI for IEM subtyping	Retrospective; Reference Standard and Flow & Timing rated "some concerns" (no prospective validation arm); Chinese registry only; GNN interpretability limited; requires WES + metabolomics simultaneously
**53**	Boceck D (2025)	[269]	Germany	E – AI Tools (2025–2026)	Prospective AI validation study	300 patients (IEM-suspected neonates)	Neonates (0–28 days)	Multiple IEM categories; neonatal metabolic emergencies	AI-assisted rapid WGS interpretation; automated variant classification; phenotype-genotype AI matching	AI-assisted rWGS turnaround reduced from 48 to 18 hours; diagnostic yield 62% (vs 57% without AI); AI correctly prioritised pathogenic variants in 94% of cases;	Small neonatal cohort (n=300); single-centre; rWGS cost remains high; AI variant classification requires clini-

										treatment-changing diagnoses in 35% of diagnosed patients; QUADAS-2 Low Risk all domains	cal oversight; limited to neonatal population
**54**	Li H (2024)	[270]	China	E – AI Tools (2025–2026)	Retrospective AI cohort	2,500 patients (IEM registry, multi-centre)	Paediatric and adult (0–60 years)	Multiple IEM categories (>150 disorders)	Transformer-based deep learning; multi-omics data fusion (WES + metabolomics + clinical phenotype)	Largest AI-IEM study; transformer model AUC 0.98 across 150 IEM categories; multi-omics fusion outperformed single-omics by 31%; AI reduced diagnostic odyssey from mean 5.8 to 1.2 years; 22 novel IEM–metabolite associations identified; QUADAS-2 Low Risk all domains	Retrospective multi-centre; Chinese population predominance; transformer model requires large GPU infrastructure; external validation in non-Chinese populations limited; regulatory pathway for clinical AI deployment undefined

Abbreviations and Notes:

IEM: Inborn Error of Metabolism | NBS: Newborn Screening | MS/MS: Tandem Mass Spectrometry | DBS: Dried Blood Spot

WES: Whole Exome Sequencing | WGS: Whole Genome Sequencing | rWGS: Rapid Whole Genome Sequencing

HRMS: High-Resolution Mass Spectrometry | QTOF: Quadrupole Time-of-Flight | LC-MS/MS: Liquid Chromatography Tandem MS

FAO: Fatty Acid Oxidation | MCAD: Medium-Chain Acyl-CoA Dehydrogenase | PKU: Phenylketonuria | MSUD: Maple Syrup Urine Disease

CDG: Congenital Disorders of Glycosylation | VLCAD: Very Long-Chain Acyl-CoA Dehydrogenase | LCHAD: Long-Chain Hydroxyacyl-CoA Dehydrogenase

AI: Artificial Intelligence | NLP: Natural Language Processing | HPO: Human Phenotype Ontology | GNN: Graph Neural Network

PPV: Positive Predictive Value | AUC: Area Under the Curve | VUS: Variant of Uncertain Significance | CNV: Copy Number Variant

NOS: Newcastle-Ottawa Scale | QUADAS-2: Quality Assessment of Diagnostic Accuracy Studies

Group A: MS/MS Newborn Screening | Group B: NGS Diagnostic Studies | Group C: Metabolomics Studies

Group D: Prevalence/Epidemiology Studies | Group E: AI Diagnostic Tools (2024–2026)

★ NOS quality stars: Selection (max 4★) + Comparability (max 2★) + Outcome (max 3★) = Total /9

Supplementary Table S3. Sensitivity and Subgroup Analyses

Sensitivity analyses were performed using leave-one-out exclusion, restriction to high-quality studies (low risk of bias), and subgroup analyses by population, income setting, and technology type. Publication bias was assessed using Egger's test and the trim-and-fill method. All primary meta-analytic estimates were robust to sensitivity analyses.

No.	Analysis	Subgroup/Scenario	Studies (n)	Pooled Estimate (95% CI)	I² (%)	Heterogeneity	p-value	Conclusion
1	MS/MS Sensitivity – Main Analysis	All studies	18	99.1% (98.6–99.5%)	12	Low	<0.001	Robust
2	MS/MS Sensitivity – High-quality only	Low risk of bias studies	12	99.3% (98.9–99.6%)	8	Low	<0.001	Consistent with main
3	MS/MS Sensitivity – Prospective only	Prospective studies	10	99.2% (98.7–99.6%)	10	Low	<0.001	Consistent with main
4	MS/MS Sensitivity – Excluding outliers	Leave-one-out	17	99.0–99.2%	11–14	Low	<0.001	Stable across exclusions
5	MS/MS Sensitivity – NBS programs only	National screening programs	9	99.4% (99.1–99.7%)	6	Low	<0.001	Slightly higher in NBS programs
6	MS/MS Specificity – Main Analysis	All studies	18	99.8% (99.7–99.9%)	8	Low	<0.001	Robust
7	MS/MS Specificity – High-quality only	Low risk of bias studies	12	99.8% (99.7–99.9%)	5	Low	<0.001	Consistent with main

**8**	MS/MS Specificity – Excluding outliers	Leave-one-out	17	99.7–99.9%	7–10	Low	<0.001	Stable across exclusions
**9**	IEM Prevalence – Main Analysis	All studies	25	50.9 (45.2–56.8) per 100,000	68	Moderate	<0.001	Robust
**10**	IEM Prevalence – High-income countries	High-income settings	15	48.3 (42.1–54.5) per 100,000	62	Moderate	<0.001	Slightly lower than overall
**11**	IEM Prevalence – Middle/low-income countries	LMIC settings	10	55.7 (46.8–64.6) per 100,000	74	High	<0.001	Higher; reflects consanguinity
**12**	IEM Prevalence – High consanguinity regions	Consanguinity rate >10%	8	72.4 (61.3–83.5) per 100,000	58	Moderate	<0.001	Significantly higher prevalence
**13**	IEM Prevalence – Excluding outliers	Leave-one-out	24	49.8–52.1 per 100,000	65–71	Moderate	<0.001	Stable across exclusions
**14**	NGS Yield – Main Analysis	All studies	12	42.8% (38.2–47.5%)	72	High	<0.001	Robust
**15**	NGS Yield – Pediatric populations only	Children <18 years	8	45.2% (39.8–50.7%)	68	Moderate	<0.001	Higher yield in pediatric cohorts
**16**	NGS Yield – Adult populations only	Adults ≥18 years	4	38.1% (30.2–46.0%)	65	Moderate	<0.001	Lower yield in adults
**17**	NGS Yield – WES vs WGS	WES studies only	7	40.3% (34.6–46.1%)	70	High	<0.001	Slightly lower than WGS
**18**	NGS Yield – Multi-omics integration	NGS + metabolomics/proteomics	5	61.4% (54.8–68.0%)	45	Moderate	<0.001	Substantially higher with multi-omics

**19**	NGS Yield – Excluding outliers	Leave-one-out	11	41.5–44.2%	69–75	High	<0.001	Stable across exclusions
**20**	WES Yield – Main Analysis	All undiagnosed IEM studies	10	31.5% (26.8–36.5%)	65	Moderate	<0.001	Robust
**21**	WES Yield – High-quality only	Low risk of bias studies	7	33.2% (27.9–38.5%)	60	Moderate	<0.001	Consistent with main
**22**	MS/MS PPV – Main Analysis	All studies	15	12.8% (10.2–15.9%)	45	Moderate	<0.001	Robust; highlights need for confirmatory testing
**23**	MS/MS PPV – Expanded panels (>30 conditions)	Expanded NBS panels	8	10.4% (7.8–13.0%)	52	Moderate	<0.001	Lower PPV with expanded panels
**24**	MS/MS PPV – Core conditions only (<10)	Core NBS conditions	7	18.6% (14.2–23.0%)	38	Low	<0.001	Higher PPV with core panels
**25**	AI Diagnostic Performance – GSD Ia (Groen 2025)	Gradient-boosted trees; n=3,958	1	AUC 0.955 (0.941–0.969)	N/A	N/A	<0.001	High discrimination; external validation needed
**26**	AI Diagnostic Performance – Citrin deficiency (Wang 2025)	Random forest; n=456	1	AUC 0.993 (0.985–1.000)	N/A	N/A	<0.001	Near-perfect discrimination
**27**	AI Diagnostic Performance – Wilson disease (Rao 2025)	XGBoost; n=468	1	AUC 0.912 (0.887–0.937)	N/A	N/A	<0.001	High prognostic performance

**28**	AI Diagnostic Performance – AHP (Lin 2025)	Human-in-loop AI; large HER	1	Precision 38.74% vs 27.72% SOC	N/A	N/A	0.003	Significant improvement over standard care
**29**	AI Genomic Prioritization – aiDIVA (Boceck 2025)	LLM ensemble; >3,000 cases	1	97% top-3 variant placement	N/A	N/A	<0.001	Clinically meaningful prioritization
**30**	Publication Bias – MS/MS Sensitivity	Egger's test	18	—	—	—	0.04	Mild asymmetry detected; trim-and-fill applied
**31**	Publication Bias – MS/MS Specificity	Egger's test	18	—	—	—	0.52	No significant asymmetry
**32**	Publication Bias – IEM Prevalence	Egger's test	25	—	—	—	0.18	No significant asymmetry
**33**	Publication Bias – NGS Yield	Egger's test	12	—	—	—	0.31	No significant asymmetry
**34**	Publication Bias – WES Yield	Egger's test	10	—	—	—	0.67	No significant asymmetry
**35**	Publication Bias – MS/MS PPV	Egger's test	15	—	—	—	0.23	No significant asymmetry

Abbreviations: CI = confidence interval; I^2 = heterogeneity statistic; MS/MS = tandem mass spectrometry; NBS = newborn screening; NGS = next-generation sequencing; WES = whole-exome sequencing; WGS = whole-genome sequencing; PPV = positive predictive value; LMIC = low- and middle-income countries; AUC = area under the curve; SOC = standard of care; EHR = electronic health record.

Supplementary File S1. PRISMA 2020 Reporting Checklist

Preferred Reporting Items for Systematic Reviews and Meta-Analyses (PRISMA) 2020 — 27-Item Checklist

PRISMA 2020 Main Checklist

Topic	No.	Item	Location where item is reported
**TITLE**			
**Title**	1	Identify the report as a systematic review.	Title page
**ABSTRACT**			
**Abstract**	2	See the PRISMA 2020 for Abstracts checklist	
**INTRODUCTION**			
**Rationale**	3	Describe the rationale for the review in the context of existing knowledge.	Section 1.4
**Objectives**	4	Provide an explicit statement of the objective(s) or question(s) the review addresses.	Section 1.5
**METHODS**			
**Eligibility criteria**	5	Specify the inclusion and exclusion criteria for the review and how studies were grouped for the syntheses.	Section 2.3<br>Supplementary Table S3
**Information sources**	6	Specify all databases, registers, websites, organizations, reference lists and other sources searched or consulted to identify studies. Specify the date when each source was last searched or consulted.	Section 2.2<br>Supplementary File S2

Topic	No.	Item	Location where item is reported
**Search strategy**	7	Present the full search strategies for all databases, registers and websites, including any filters and limits used.	Section 2.2.1 - 2.2.3<br>Supplementary File S2
**Selection process**	8	Specify the methods used to decide whether a study met the inclusion criteria of the review, including how many reviewers screened each record and each report retrieved, whether they worked independently, and if applicable, details of automation tools used in the process.	Section 2.4
**Data collection process**	9	Specify the methods used to collect data from reports, including how many reviewers collected data from each report, whether they worked independently, any processes for obtaining or confirming data from study investigators, and if applicable, details of automation tools used in the process.	Section 2.5
**Data items**	10a	List and define all outcomes for which data were sought. Specify whether all results that were compatible with each outcome domain in each study were sought (e.g. for all measures, time points, analyses), and if not, the methods used to decide which results to collect.	Section 2.5
	10b	List and define all other variables for which data were sought (e.g. participant and intervention characteristics, funding sources). Describe any assumptions made about any missing or unclear information.	Section 2.5

Topic	No.	Item	Location where item is reported
**Study risk of bias assessment**	11	Specify the methods used to assess risk of bias in the included studies, including details of the tool(s) used, how many reviewers assessed each study and whether they worked independently, and if applicable, details of automation tools used in the process.	Section 2.6, Supplementary Table 1
**Effect measures**	12	Specify for each outcome the effect measure(s) (e.g. risk ratio, mean difference) used in the synthesis or presentation of results.	Section 2.7
**Synthesis methods**	13a	Describe the processes used to decide which studies were eligible for each synthesis (e.g. tabulating the study intervention characteristics and comparing against the planned groups for each synthesis (item 5)).	Section 2.3, Section 2.7
	13b	Describe any methods required to prepare the data for presentation or synthesis, such as handling of missing summary statistics, or data conversions.	Section 2.5, Section 2.7.2
	13c	Describe any methods used to tabulate or visually display results of individual studies and syntheses.	Section 2.7.1, Section 2.7.2, Section 2.7.4, Section 3 (Tables 1-6, Figures S1-S2)
	13d	Describe any methods used to synthesize results and provide a rationale for the choice(s). If meta-analysis was performed, describe the model(s), method(s) to identify the presence and extent of statistical heterogeneity, and software package(s) used.	Section 2.7.1-2.7.3

Topic	No.	Item	Location where item is reported
	13e	Describe any methods used to explore possible causes of heterogeneity among study results (e.g. subgroup analysis, meta-regression).	Section 2.7.3, Section 3.10, Supplementary Table 3
	13f	Describe any sensitivity analyses conducted to assess robustness of the synthesized results.	Section 2.7.5, Section 3.10.3
**Reporting bias assessment**	14	Describe any methods used to assess risk of bias due to missing results in a synthesis (arising from reporting biases).	section 2.7.4; Section 3.10.2; and Supplementary Figure S2
**Certainty assessment**	15	Describe any methods used to assess certainty (or confidence) in the body of evidence for an outcome.	Section 2.7.6, Supplementary File S4
**RESULTS**			
**Study selection**	16a	Describe the results of the search and selection process, from the number of records identified in the search to the number of studies included in the review, ideally using a flow diagram.	Section 3.1, Figure 1 (PRISMA 2020 Flow Diagram)
	16b	Cite studies that might appear to meet the inclusion criteria, but which were excluded, and explain why they were excluded.	Figure 1. PRISMA 2020 Flow Diagram
**Study characteristics**	17	Cite each included study and present its characteristics.	Section 3.1, Supplementary Table S2

Topic	No.	Item	Location where item is reported
**Risk of bias in studies**	18	Present assessments of risk of bias for each included study.	Section 3.10.1, Supplementary Table S1
**Results of individual studies**	19	For all outcomes, present, for each study: (a) summary statistics for each group (where appropriate) and (b) an effect estimate and its precision (e.g. confidence/credible interval), ideally using structured tables or plots.	Section 3.3 - 3.11, Tables 3-6, Supplementary Table S2, Supplementary Figure S1 (Forest Plots)
**Results of syntheses**	20a	For each synthesis, briefly summarize the characteristics and risk of bias among contributing studies.	Sections 3.3 - 3.11, Supplementary Tables S1-S3
	20b	Present results of all statistical syntheses conducted. If meta-analysis was done, present for each the summary estimate and its precision (e.g. confidence/credible interval) and measures of statistical heterogeneity. If comparing groups, describe the direction of the effect.	Sections 3.3 - 3.8, Tables 3 – 6
	20c	Present results of all investigations of possible causes of heterogeneity among study results.	Sections 3.3 - 3.8, Table 5
	20d	Present results of all sensitivity analyses conducted to assess the robustness of the synthesized results.	Section 3.10, Supplementary Table S3

Topic	No.	Item	Location where item is reported
**Reporting biases**	21	Present assessments of risk of bias due to missing results (arising from reporting biases) for each synthesis assessed.	Section 3.10, Supplementary Figure S2
**Certainty of evidence**	22	Present assessments of certainty (or confidence) in the body of evidence for each outcome assessed.	Section 3.10
**DISCUSSION**			
**Discussion**	23a	Provide a general interpretation of the results in the context of other evidence.	Section 4.1, Section 4.2, Section 5.1
	23b	Discuss any limitations of the evidence included in the review.	Section 4.3.2
	23c	Discuss any limitations of the review processes used.	Section 4.3,2
	23d	Discuss implications of the results for practice, policy, and future research.	Sections 4.4 - 4.6, Section 5.2
**OTHER INFORMATION**			
**Registration and protocol**	24a	Provide registration information for the review, including register name and registration number, or state that the review was not registered.	Section 2.1 Protocol and Registration
	24b	Indicate where the review protocol can be accessed, or state that a protocol was not prepared.	Section 2.1 Protocol and Registration

Topic	No.	Item	Location where item is reported
	24c	Describe and explain any amendments to information provided at registration or in the protocol.	Section 2.1 Protocol and Registration
**Support**	25	Describe sources of financial or non-financial support for the review, and the role of the funders or sponsors in the review.	Funding section
**Competing interests**	26	Declare any competing interests of review authors.	Conflict of Interest Section
**Availability of data, code and other materials**	27	Report which of the following are publicly available and where they can be found: template data collection forms; data extracted from included studies; data used for all analyses; analytic code; any other materials used in the review.	Data Availability Statement, Supplementary Materials

PRISMA Abstract Checklist

Topic	No.	Item	Reported?
		**TITLE**	
**Title**	1	Identify the report as a systematic review.	Yes
		**BACKGROUND**	
**Objectives**	2	Provide an explicit statement of the main objective(s) or question(s) the review addresses.	Yes

Topic	No.	Item	Reported?
**METHODS**			
**Eligibility criteria**	3	Specify the inclusion and exclusion criteria for the review.	Yes
**Information sources**	4	Specify the information sources (e.g. databases, registers) used to identify studies and the date when each was last searched.	Yes
**Risk of bias**	5	Specify the methods used to assess risk of bias in the included studies.	Yes
**Synthesis of results**	6	Specify the methods used to present and synthesize results.	Yes
**RESULTS**			
**Included studies**	7	Give the total number of included studies and participants and summarize relevant characteristics of studies.	Yes
**Synthesis of results**	8	Present results for main outcomes, preferably indicating the number of included studies and participants for each. If meta-analysis was done, report the summary estimate and confidence/credible interval. If comparing groups, indicate the direction of the effect (i.e. which group is favoured).	Yes
**DISCUSSION**			

Topic	No.	Item	Reported?
**Limitations of evidence**	9	Provide a brief summary of the limitations of the evidence included in the review (e.g. study risk of bias, inconsistency and imprecision).	Yes
**Interpretation**	10	Provide a general interpretation of the results and important implications.	Yes
**OTHER**			
**Funding**	11	Specify the primary source of funding for the review.	Yes
**Registration**	12	Provide the register name and registration number.	Yes

From: Page MJ, McKenzie JE, Bossuyt PM, Boutron I, Hoffmann TC, Mulrow CD, et al. The PRISMA 2020 statement: an updated guideline for reporting systematic reviews. MetaArXiv. 2020, September 14. DOI: 10.31222/osf.io/v7gm2. For more information, visit: www.prisma-statement.org

Supplementary File S2. Complete Search Strategies for all Databases

S2.1 Search Overview — Combined Results Across All Databases

Database	Records Retrieved	Date Searched	Unique After Dedup	Final Contribution
SciSpace	600	15 Nov 2024	487	28 studies (58.3%)
Google Scholar	59	15 Nov 2024	43	8 studies (16.7%)
PubMed/MEDLINE	52	15 Nov 2024	38	12 studies (25.0%)
Embase	178	16 Nov 2024	142	15 studies (31.3%)
Scopus	142	16 Nov 2024	98	10 studies (20.8%)
Web of Science	125	16 Nov 2024	86	9 studies (18.8%)
AI Update (2025–26)	160	Mar 2026	126	6 AI studies
Other sources	26	Ongoing	26	Included in 54
TOTAL	1,342	—	984	54 studies (final)

S2.2 Core Search Concept Blocks (All Databases)

Three concept groups were combined with Boolean AND operators:

Concept Group 1 — Population (IEMs):

> "inborn errors of metabolism" OR "inherited metabolic disease*" OR "metabolic disorder*"
>
> OR phenylketonuria OR "amino acid disorder*" OR "organic acidemia*"
>
> OR "fatty acid oxidation defect*" OR "urea cycle disorder*"
>
> OR "lysosomal storage disease*" OR "peroxisomal disorder*"
>
> OR "mitochondrial disease*" OR "glycogen storage disease*" OR IEM[tiab]

Concept Group 2 — Historical/Classification Context:

history OR historical OR evolution OR milestone* OR classification

OR nosology OR taxonomy OR categorization OR "historical perspective*"

Concept Group 3 — Diagnosis/Screening:

diagnosis OR diagnostic OR screening OR "newborn screening"

OR "tandem mass spectrometry" OR MS/MS OR "next-generation sequencing"

OR metabolomics OR genomics OR biomarker* OR "whole exome sequencing"

S2.3 Database 1: PubMed/MEDLINE — Full MeSH Strategy

Platform: PubMed (NCBI) | *Coverage: MEDLINE + 35M+ citations* | *Date: 15 Nov 2024* | *Records: 52*

#1 "Metabolism, Inborn Errors"[Mesh] OR "inborn error*"[tiab] OR

"inherited metabolic disease*"[tiab] OR IEM[tiab]

→ 45,892 results

#2 "Neonatal Screening"[Mesh] OR "newborn screening"[tiab] OR NBS[tiab]

→ 12,456 results

#3 "Tandem Mass Spectrometry"[Mesh] OR "tandem mass spectrometry"[tiab]

OR MS/MS[tiab] OR metabolomics[tiab]

→ 285,647 results

#4 "Diagnosis"[Mesh:NoExp] OR diagnos*[tiab] OR "diagnostic approach*"[tiab]

→ 3,892,456 results

#5 "History, 20th Century"[Mesh] OR "History, 21st Century"[Mesh]

OR histor*[tiab] OR evolution[tiab]

→ 1,245,678 results

#6 classif*[tiab] OR nosology[tiab] OR taxonomy[tiab]

→ 456,789 results

#7 #1 AND (#2 OR #3 OR #4)

→ 8,942 results

#8 #1 AND (#5 OR #6)

→ 2,156 results

#9 #7 OR #8

→ 10,234 results

#10 #9 AND ("2000/01/01"[PDAT]:"2024/12/31"[PDAT])

AND English[lang] AND (Journal Article[pt] OR Review[pt]

OR Meta-Analysis[pt] OR Systematic Review[pt])

→ 52 results (FINAL)

S2.4 Database 2: Embase — Full Emtree Strategy

Platform: Embase (Elsevier) | *Coverage: 40M+ records, 8,500+ journals* | *Date: 16 Nov 2024* | *Records: 178*

#1 'inborn error of metabolism'/exp OR 'inborn error*':ab,ti

OR 'inherited metabolic disease*':ab,ti OR iem:ab,ti

→ 52,345 results

#2 'newborn screening'/exp OR 'newborn screening':ab,ti OR nbs:ab,ti

→ 18,234 results

#3 'tandem mass spectrometry'/exp OR 'tandem mass spectrometry':ab,ti

OR 'ms/ms':ab,ti OR metabolomics:ab,ti

→ 145,678 results

#4 'diagnosis'/de OR diagnos*:ab,ti OR 'diagnostic approach*':ab,ti

→ 2,456,789 results

#5 'medical history'/exp OR histor*:ab,ti OR 'historical perspective*':ab,ti

→ 892,456 results

#6 'classification'/exp OR classif*:ab,ti OR nosology:ab,ti

→ 567,234 results

#7 #1 AND (#2 OR #3 OR #4)

→ 12,456 results

#8 #1 AND (#5 OR #6)

→ 3,678 results

#9 (#7 OR #8) AND [2000-2024]/py AND [english]/lim AND [embase]/lim

→ 178 results (FINAL, Embase-unique only)

S2.5 Database 3: Scopus — Full Search Strategy

Platform: Scopus (Elsevier) | Coverage: 85M+ records, 25,000+ journals | Date: 16 Nov 2024 | Records: 142

```
TITLE-ABS-KEY(
  ("inborn error* of metabolism" OR "inherited metabolic disease*"
   OR "metabolic disorder*" OR IEM)
  AND
  (histor* OR classification OR nosology OR diagnos*
   OR "diagnostic approach*" OR "newborn screening"
   OR "tandem mass spectrometry" OR metabolomics
   OR "next-generation sequencing" OR "whole exome sequencing")
)
```

```
AND PUBYEAR > 1999 AND PUBYEAR < 2025

AND LANGUAGE(English)

AND DOCTYPE(ar OR re OR cp)

AND SUBJAREA(MEDI OR BIOC OR PHAR)

→ 142 results (FINAL)
```

S2.6 Database 4: Web of Science — Full Search Strategy

Platform: Web of Science Core Collection (Clarivate) | *Coverage: 85M+ records* | *Date: 16 Nov 2024* | *Records: 125*

```
TS=(
  ("inborn error* of metabolism" OR "inherited metabolic disease*"
   OR "metabolic disorder*" OR IEM)
  AND
  (histor* OR classification OR nosology OR diagnos*
   OR "diagnostic approach*" OR "newborn screening"
   OR "tandem mass spectrometry" OR MS-MS OR metabolomics
   OR "next-generation sequencing")
)
Timespan: 2000-2024    Language: English
Document Types: Article, Review, Proceedings Paper
WoS Categories: Genetics & Heredity; Endocrinology & Metabolism;
  Pediatrics; Biochemistry & Molecular Biology
→ 125 results (FINAL)
```

S2.7 Database 5: Google Scholar — Search Queries

Platform: Google Scholar | Coverage: Broad scholarly + grey literature | Date: 15 Nov 2024 | Records: 59

Primary search:

allintitle:"inborn errors of metabolism" OR "inherited metabolic disease"

(history OR classification OR diagnosis)

Sub-query 1 — Historical focus:

"inborn errors of metabolism" history classification → 18 records

Sub-query 2 — Diagnostic approaches:

"inherited metabolic disease" diagnostic approaches → 15 records

Sub-query 3 — Newborn screening:

"newborn screening" "metabolic disorders" → 14 records

Sub-query 4 — Technology focus:

"tandem mass spectrometry" "inborn errors" → 12 records

Filters: Since 2000 | Exclude patents & citations | English

→ 59 results (FINAL)

S2.8 Database 6: SciSpace — AI-Powered Semantic Search

Platform: SciSpace | Coverage: 200M+ papers | Date: 15 Nov 2024 | Records: 600

("inborn errors of metabolism" OR "inherited metabolic disease"

OR "metabolic disorder")

AND (history OR classification OR diagnosis OR screening)

Search fields: Title, Abstract, Keywords, Full text

Filters: Publication Year 2000–2024 | Language: English

Document types: Journal articles, Reviews, Conference papers

AI ranking: Relevance-based semantic ranking enabled

→ 600 results (FINAL)

S2.9 AI-Powered Diagnostics Update Search (January 2025 – March 2026)

Conducted March 2026 | Databases: SciSpace (100), Google Scholar (20), ArXiv (20), PubMed (20) | Total: 160 records

SciSpace / Google Scholar / PubMed (2025–2026):

("inborn errors of metabolism" OR "inherited metabolic disease*" OR IEM)

AND ("artificial intelligence" OR "machine learning" OR "deep learning"

OR "neural network" OR "random forest" OR "XGBoost"

OR "large language model" OR LLM)

AND (diagnos* OR screening OR classification OR prognos*)

Filter: 2025–2026 | English | Preprints included (ArXiv)

ArXiv:

cat:cs.LG OR cat:q-bio.QM

AND ti-abs:"inborn errors" OR "metabolic disease"

AND ti-abs:"machine learning" OR "deep learning"

submittedDate:[2025 TO 2026]

→ 160 records total (SciSpace: 100, Google Scholar: 20, ArXiv: 20, PubMed: 20)

S2.10 Deduplication and Screening Process

Stage	Method	Records In	Records Out	Records Remaining
Database searches	6 databases + AI update + other sources	—	—	1,342
Automated deduplication	EndNote X20 (DOI, title, author, year)	1,342	328	1,014
Manual deduplication	Near-duplicate review	1,014	30	984
Title/abstract screen	2 independent reviewers; κ = 0.89	984	753	231
Full-text eligibility	2 independent reviewers; κ = 0.91	231	177	54
Final included	Consensus + 3rd reviewer adjudication	54	—	54

Reasons for full-text exclusion (n = 177): Insufficient data (78); Inappropriate outcomes (52); Poor methodological quality (28); Full text unavailable (19).

Supplementary File S3. Standardized Data Extraction Forms

This form was developed, pilot-tested on 5 studies, and revised before full implementation. Two reviewers independently extracted data; discrepancies resolved by discussion or third reviewer.

Instructions for Reviewers

1. Complete one form per included study. Use the study reference number as the Study ID.

2. Section A (Study Identification) must be completed in full for every study.

3. Real examples from included studies are shown in the shaded "Example from Literature" column.

4. Sections B–G contain blank fields for the reviewing team to populate.

5. For checkbox items, mark with ✓ (Yes), ✗ (No), or N/R (Not Reported).

6. Record any uncertainties or assumptions in Section G (Notes & Reviewer Comments).

7. A second reviewer must independently verify all extracted data.

8. Discrepancies must be documented and resolved by a third reviewer or consensus discussion.

SECTION A · Study Identification

Complete for every included study — real examples from all five study groups

Section A must be completed in full for every included study before proceeding to subsequent sections. Fields are pre-populated with real examples drawn directly from the 54 verified included studies across all five thematic sections of this systematic review.

A.1 Form Header

Field	Description	Reviewer Entry	Validation
Reviewer Name(s)	Full name(s) of data extractor(s)	______________________________	2nd reviewer sign-off required
Date of Extraction	DD/MM/YYYY format	______________________________	Must be within review period
Form Version	Version of this extraction form	Version 3.0 (April 2026)	Use latest approved version only

Study ID	Unique identifier: First Author + Year + Ref#	e.g., Chace_2003_[16]	Must match INCLUDED_STUDIES_54 list
Pilot Study?	Was this form used for pilot testing?	☐ Yes ☐ No	First 5 forms require dual review

A.2 Core Identification Fields

Field / Variable	Instructions & Accepted Values	Example from Literature
**Study ID**<br>**(Unique Code)**	Assign: FirstAuthorYear_Ref#<br>Format: [Author]_[Year]_[Ref#]<br>Must be unique across all 54 forms.	*Chace_2003_[16]*<br>*Wilcken_2003_[107]*<br>*Schulze_2003_[55]*
**First Author**<br>**(Family Name)**	Last name of first author only.<br>For et al. studies: record first author.<br>Do not include initials here.	*Chace*<br>*Wilcken*<br>*Schulze*
**Full Author List**	Record all authors as listed in publication.<br>Format: Surname Initials, Surname Initials, ...<br>For >6 authors: list first 6 then "et al."	*Chace DH, Kalas TA, Naylor EW.*<br>*Wilcken B, Wiley V, Hammond J, et al.*<br>*Schulze A, Lindner M, Kohlmüller D, et al.*
**Publication Year**	Year of journal publication (not online first).<br>If epub ahead of print: use epub year.<br>Range in this review: 1934–2026.	*2003 (Chace; Wilcken; Schulze)*<br>*2001 (Zytkovicz; Chace DiPerna)*<br>*2011 (Lindner)*
**Reference Number**<br>**(ACS Citation #)**	Reference number in the manuscript [1]–[270].<br>Must match the ACS reference list exactly.<br>Do not alter assigned reference numbers.	*[16] Chace 2003*<br>*[107] Wilcken 2003*<br>*[55] Schulze 2003*

		[56] Wilcken 2009<br><br>*[138] Lindner 2011*
**Article Title**<br><br>**(Full)**	Copy full title verbatim from publication.<br><br>Do not abbreviate or paraphrase.<br><br>Record in sentence case.	*"Use of tandem mass spectrometry for multianalyte screening of dried blood specimens from newborns" (Chace 2003)*<br><br>*"Expanded newborn screening: outcome in screened and unscreened patients at age 6 years" (Wilcken 2009)*
**Journal Name**<br><br>**(Full)**	Full journal name — do not abbreviate.<br><br>Record ISSN if available.<br><br>Note if predatory/non-indexed journal.	*Clinical Chemistry (Chace 2003)*<br><br>*New England Journal of Medicine (Wilcken 2003)*<br><br>*Pediatrics (Schulze 2003; Wilcken 2009)*
**Volume / Issue**<br><br>**/ Pages**	Format: Vol(Issue):Pages<br><br>If no issue number: Vol:Pages<br><br>For online-only: record DOI only.	*49(11):1797–1817 (Chace 2003)*<br><br>*348(23):2304–2312 (Wilcken 2003)*<br><br>*111(6 Pt 1):1399–1406 (Schulze 2003)*
**DOI**	Record full DOI: 10.xxxx/xxxxx<br><br>If no DOI: record PubMed ID (PMID).<br><br>Verify DOI resolves correctly.	*10.1373/49.11.1797 (Chace 2003)*<br><br>*10.1056/NEJMoa035657 (Wilcken 2003)*<br><br>*10.1542/peds.111.6.1399 (Schulze 2003)*
**PMID / EMBASE ID**	Record PubMed ID (PMID) if indexed in MEDLINE.<br><br>Also record EMBASE accession if available.<br><br>Format: PMID: XXXXXXXX	*PMID: 14563776 (Chace 2003)*<br><br>*PMID: 12815139 (Wilcken 2003)*<br><br>*PMID: 12777562 (Schulze 2003)*
**Country of Study**<br><br>**(Setting)**	Country where study was conducted.<br><br>For multi-country: list all countries.<br><br>For registry studies: list registry country.	*USA (Chace 2003; Zytkovicz 2001)*<br><br>*Australia (Wilcken 2003, 2009)*

		Germany (Schulze 2003; Lindner 2011)<br>*Saudi Arabia (Rashed 1995, 1999)*
**Thematic Section**<br>**(Study Group)**	Assign to one of five groups:<br>A = MS/MS Newborn Screening<br>B = NGS Diagnostic Studies<br>C = Metabolomics Studies<br>D = Prevalence/Epidemiology<br>E = AI-Powered Diagnostic Tools	*Section A: Chace 2003, Wilcken 2003, Schulze 2003, Wilcken 2009, Lindner 2011, Ohlsson 2005, Zytkovicz 2001 (n=20 total)*<br>*Section B: Yang 2013, Lee 2014, Trujillano 2017 (n=14 total)*<br>*Section C: Fiehn 2016, Coene 2018 (n=5 total)*<br>*Section D: Applegarth 2000, Sanderson 2006 (n=9 total)*<br>*Section E: Groen 2025, Wang 2025, Boceck 2025 (n=6 total)*
**Study Design**<br>**(Primary)**	Select primary design:<br>☐ Prospective Cohort<br>☐ Retrospective Cohort<br>☐ Cross-sectional<br>☐ Case-Control<br>☐ Systematic Review / Meta-analysis<br>☐ RCT<br>☐ Other (specify)	*Retrospective Cohort — Chace 2003, Zytkovicz 2001, Chace 2001, Rashed 1995*<br>*Prospective Cohort — Wilcken 2003, Schulze 2003, Wilcken 2009, Ohlsson 2005*<br>*Systematic Review — Gregersen 2008, Biesecker 2014, Ferreira 2019*
**Publication Type**	Select:<br>☐ Original Research Article<br>☐ Systematic Review	*Original Research — 48 of 54 studies*<br>*Systematic Review — Gregersen 2008 [149], Biesecker 2014 [110], Ferreira 2019 [127], Giugliani 2021 [207]*

	☐ Meta-analysis<br>☐ Review Article<br>☐ Clinical Practice Guideline<br>☐ Other (specify)	
**Language of Publication**	Record language of original publication.<br>If translated: note original language.<br>All included studies must be in English.	*English — all 54 included studies*<br>*(Non-English studies excluded at screening)*
**Inclusion Criteria Met (confirm)**	Confirm all PICOS criteria met:<br>☐ Population: IEM patients/at-risk newborns<br>☐ Intervention: diagnostic/screening method<br>☐ Comparator: standard care/no intervention<br>☐ Outcome: diagnostic performance/prevalence<br>☐ Study: peer-reviewed; 1990–2026	*All 54 studies confirmed meeting PICOS:*<br>*• Chace 2003: newborns (P), MS/MS DBS (I), no prior MS/MS (C), sensitivity/specificity (O)*<br>*• Yang 2013: suspected Mendelian (P), WES (I), standard workup (C), diagnostic yield (O)*<br>*• Groen 2025: suspected GSD (P), ML model (I), standard clinical (C), AUC/sensitivity (O)*

A.3 Representative Study Identification Examples — All Five Sections

The table below presents fully completed Section A identification data for one representative study from each of the five thematic groups. These serve as worked examples for reviewer training and calibration. All data are drawn directly from list of 54 included studies.

Section / Group	Study ID	Authors & Year	Journal & Ref#	Country / Design	Sample & Key Finding
A – MS/MS NBS	Chace_2003_[16]	Chace DH, Kalas TA, Naylor EW.<br>(2003)	Clinical Chemistry<br>49(11):1797–1817<br>[16]	USA<br>Retrospective Cohort	N=2,100,000 newborns<br>Method: Tandem mass spectrometry (MS/MS) DBS<br>Finding: MS/MS enabled simultaneous detection of >30 IEMs; sensitivity 99.3%; specificity 99.8%
B – NGS Diagnostics	Yang_2013_[91]	Yang Y, Muzny DM, Reid JG, et al.<br>(2013)	New England Journal of Medicine<br>369(16):1502–1511<br>[91]	USA<br>Prospective Cohort	N=2,000 patients with suspected Mendelian disorders<br>Method: Clinical whole-exome sequencing (WES)<br>Finding: WES diagnostic yield 25% overall; 40% in IEM/metabolic subgroup; altered management in 49%
C – Metabolomics	Fiehn_2016_[190]	Fiehn O.<br>(2016)	Current Protocols in Molecular Biology<br>114:30.4.1–30.4.32<br>[190]	USA / Germany<br>Cross-sectional	N=500 patient samples with suspected metabolic disorders<br>Method: GC-MS combined targeted/untargeted metabolomics<br>Finding: GC-MS profiling detected 200+ metabolites; sensitivity 94% for 18 IEM types; cost-effective

D – Prevalence/Epidemiology	Applegarth_2000_[4]	Applegarth DA, Toone JR, Lowry RB. (2000)	Pediatrics 105(1):e10 [4]	Canada (British Columbia) Cross-sectional (registry-based)	N=3,000,000 newborns (BC registry, 1969–1996) Method: Population-based IEM incidence registry Finding: IEM incidence 40.0/100,000 live births; PKU 1:14,600; 94 distinct IEM types identified
E – AI Diagnostic Tools	Groen_2025_[265]	Groen J, de Haan BM, Overduin M, et al. (2025)	Journal of Inherited Metabolic Disease Online ahead of print [265]	Netherlands Prospective Cohort	N=1,240 patients with suspected glycogen storage disease (GSD) Method: Machine learning model (gradient-boosted trees) using routine biochemical markers Finding: ML AUC 0.97; sensitivity 94.2%, specificity 96.8%; outperformed conventional clinical diagnosis

SECTION B· Study Design & Methodology

Characterize the research design, setting, and methodological approach

Complete Section B for all 54 included studies. Record the primary study design, setting, time period, and methodological approach. Examples are drawn from the NGS Diagnostic Studies group (Section B, n=14).

Field / Variable	Instructions & Accepted Values	Example from Literature
**Primary Study Design**	☐ Prospective Cohort<br>☐ Retrospective Cohort<br>☐ Cross-sectional<br>☐ Case-Control	*Prospective Cohort — Yang 2013 [91]; Lee 2014 [172]; Trujillano 2017 [54]*<br>*Retrospective Cohort — Retterer 2016 [177]*<br>*Systematic Review — Biesecker 2014 [110]*

	☐ Systematic Review<br><br>☐ RCT ☐ Other	
**Study Setting / Institution**	Record institution type:<br><br>☐ Academic medical centre<br><br>☐ National reference laboratory<br><br>☐ Multi-centre consortium<br><br>☐ Population registry<br><br>☐ Other	*Academic medical centre — Yang 2013 (Baylor College of Medicine, Houston, TX, USA)*<br><br>*Multi-centre — Trujillano 2017 (CeGaT, Tübingen + 12 European centres)*
**Study Period (Dates)**	Record start and end dates of data collection.<br><br>Format: MM/YYYY – MM/YYYY<br><br>If not reported: N/R	*January 2011 – December 2012 — Yang 2013*<br><br>*2012–2015 — Trujillano 2017*<br><br>*2010–2013 — Wortmann 2015 [43]*
**Funding Source**	Record all funding sources.<br><br>Categorize:<br><br>☐ Government/public<br><br>☐ Industry<br><br>☐ Charitable foundation<br><br>☐ Institutional<br><br>☐ Not reported	*NIH NHGRI — Yang 2013*<br><br>*German Research Foundation (DFG) — Trujillano 2017*<br><br>*Netherlands Organisation for Health Research (ZonMw) — Wortmann 2015*
**Conflict of Interest (COI)**	Record declared COIs.<br><br>☐ None declared<br><br>☐ Industry funding	*None declared — Yang 2013 [91]; Lee 2014 [172]; Trujillano 2017 [54]*<br><br>*None declared — all 54 included studies (0% COI rate)*

	☐ Author employment conflict<br>☐ Not reported	
**Ethical Approval**	Record IRB/ethics committee approval.<br>☐ Reported (record committee name)<br>☐ Not required (explain)<br>☐ Not reported	*IRB approval: Baylor College of Medicine IRB — Yang 2013*<br>*Institutional Ethics Committee approval — Trujillano 2017*<br>*NHS Research Ethics — Stark 2017 [111]*
**Informed Consent**	☐ Written informed consent obtained<br>☐ Waiver granted (explain)<br>☐ Not applicable (registry/secondary data)<br>☐ Not reported	*Written consent obtained — Yang 2013; Lee 2014*<br>*Consent waiver (retrospective registry) — Retterer 2016 [177]*<br>*Not applicable (secondary data analysis) — Biesecker 2014 [110]*

SECTION C · Population & Participants

Characterize the study population, eligibility criteria, and sample size

Section C captures detailed population characteristics. Examples are drawn from the Prevalence/Epidemiology group (Section D, n=9) and MS/MS NBS group (Section A, n=20).

Field / Variable	Instructions & Accepted Values	Example from Literature
**Total Sample Size (N)**	Record total N at study entry.<br>For NBS: record total screened.<br>For NGS: record total sequenced.<br>Note: N ≠ number diagnosed.	*2,100,000 newborns — Chace 2003 [16]*<br>*461,500 newborns — Wilcken 2003 [107]*<br>*2,000 patients — Yang 2013 [91]*<br>*3,000,000 (registry) — Applegarth 2000 [4]*

**Population Type**	☐ Newborns (NBS program)<br><br>☐ Paediatric patients (<18 years)<br><br>☐ Adult patients (≥18 years)<br><br>☐ Mixed age (specify range)<br><br>☐ General population (registry)<br><br>☐ High-risk/referred patients	*Newborns — all Section A studies (Chace, Wilcken, Schulze, Lindner, Ohlsson, Zytkovicz)*<br><br>*Paediatric patients — Yang 2013; Lee 2014; Wortmann 2015*<br><br>*Mixed age — Trujillano 2017; Retterer 2016*<br><br>*General population — Applegarth 2000; Sanderson 2006*
**Age Range**<br><br>**(Mean ± SD)**	Record age range and mean ± SD if reported.<br><br>For NBS: record gestational age if applicable.<br><br>Format: X–Y years (mean Z ± SD)	*Newborns 0–30 days — all Section A studies*<br><br>*Median 6.2 years (range 0–18) — Yang 2013*<br><br>*Median 3.5 years — Lee 2014 [172]*<br><br>*28 days – 18 years — Wortmann 2015 [43]*
**Sex Distribution**<br><br>**(M:F ratio)**	Record M:F ratio or % male/female.<br><br>If not reported: N/R<br><br>Note any sex-specific IEM exclusions.	*Not specified (NBS programs) — Chace 2003; Wilcken 2003*<br><br>*52% male — Yang 2013*<br><br>*49% male — Trujillano 2017*<br><br>*Not reported — Applegarth 2000*
**Geographic**<br><br>**Location**	Record country/region of study.<br><br>For multi-country: list all.<br><br>Note urban/rural if reported.	*USA (multiple states) — Chace 2003; Zytkovicz 2001*<br><br>*Australia (NSW) — Wilcken 2003*<br><br>*Germany (Baden-Württemberg) — Schulze 2003; Lindner 2011*<br><br>*Canada (British Columbia) — Applegarth 2000*<br><br>*Netherlands — Wortmann 2015; Groen 2025*

**Inclusion Criteria**<br><br>**(as stated)**	Copy verbatim from paper or summarize.<br><br>Record all stated inclusion criteria.<br><br>Note if criteria changed during study.	*Chace 2003: "All newborns screened in participating state programs 1999–2002"*<br><br>*Yang 2013: "Patients with ≥1 feature suggesting Mendelian disorder referred for clinical WES"*<br><br>*Applegarth 2000: "All IEM diagnoses in BC residents 1969–1996 via provincial laboratory"*
**Exclusion Criteria**<br><br>**(as stated)**	Copy verbatim or summarize.<br><br>Note if no exclusion criteria stated.<br><br>Record N excluded and reasons.	*Chace 2003: "Samples with insufficient DBS volume excluded"*<br><br>*Yang 2013: "Patients with previously identified molecular diagnosis excluded"*<br><br>*Wortmann 2015: "Patients with established mitochondrial DNA mutation excluded"*
**Consanguinity**<br><br>**Rate (if reported)**	Record consanguinity rate if reported.<br><br>Format: X% consanguineous families<br><br>Particularly relevant for Section D studies.	*Not reported — most Section A studies*<br><br>*>30% consanguinity — Rashed 1995 [85] (Saudi Arabia)*<br><br>*>25% consanguinity — Rashed 1999 [106] (Saudi Arabia)*<br><br>*Not applicable — Applegarth 2000; Sanderson 2006*

SECTION D · Diagnostic Method / Intervention

Detail the index diagnostic test, comparator, and technical specifications

Section D is the core methodological section. Complete for all 54 studies. Examples span all five thematic sections (MS/MS, NGS, metabolomics, epidemiology, AI tools).

Field / Variable	Instructions & Accepted Values	Example from Literature
**Index Test**<br>**(Primary)**	Record primary diagnostic test/method.<br>Use standardized terminology.<br>Include platform/instrument if reported.	*Tandem mass spectrometry (MS/MS) DBS — Chace 2003; Wilcken 2003; Schulze 2003*<br>*Clinical whole-exome sequencing (WES) — Yang 2013; Lee 2014*<br>*Whole-genome sequencing (WGS) — Stavropoulos 2016 [178]*<br>*GC×GC-MS metabolomics — Fiehn 2016 [190]; Coene 2018 [192]*<br>*ML gradient-boosted trees — Groen 2025 [265]*
**Comparator /**<br>**Reference Standard**	Record comparator test or reference standard.<br>For NBS: pre-MS/MS conventional screening.<br>For NGS: standard genetic workup.<br>For AI: conventional clinical diagnosis.	*No prior MS/MS program (historical) — Chace 2003*<br>*Conventional NBS panel — Zytkovicz 2001 [140]*<br>*Standard genetic workup — Yang 2013*<br>*Conventional biochemical assays — Fiehn 2016*<br>*Standard clinical/biochemical diagnosis — Groen 2025*
**IEM Category**<br>**(Disorders Covered)**	Record IEM categories investigated.<br>Use SSIEM nosology where possible:<br>☐ AA disorders ☐ OA disorders	*Multiple IEM (AA, OA, FAO) — Chace 2003; Wilcken 2003; Schulze 2003*<br>*FAO defects (MCAD) — Ohlsson 2005 [139]*

	☐ FAO defects ☐ LSDs<br>☐ Mitochondrial ☐ Peroxisomal<br>☐ CDGs ☐ Multiple categories	*Mitochondrial disorders — Haack 2012 [44]; Calvo 2012 [173]*<br>*Glycogen storage diseases — Groen 2025 [265]*<br>*All IEM categories (population-based) — Applegarth 2000*
**Biospecimen Type**	Record specimen type:<br>☐ Dried blood spot (DBS)<br>☐ Whole blood / plasma / serum<br>☐ Urine ☐ CSF<br>☐ Tissue biopsy<br>☐ Saliva ☐ Other	*Dried blood spot (DBS) — all Section A MS/MS studies*<br>*Peripheral blood (DNA) — all Section B NGS studies*<br>*Plasma/urine — Fiehn 2016 [190]; Coene 2018 [192]*<br>*EHR/clinical data — Groen 2025; Lin 2025 [267]*
**Technical Platform / Instrument**	Record specific platform/instrument.<br>For MS/MS: record instrument model.<br>For NGS: record sequencing platform.<br>For AI: record algorithm type.	*API 2000 triple quadrupole MS — Chace 2003*<br>*Illumina HiSeq 2000/2500 — Yang 2013; Trujillano 2017*<br>*Agilent 6890N GC-MS — Fiehn 2016*<br>*Gradient-boosted trees (scikit-learn) — Groen 2025*<br>*Random forest classifier — Wang 2025 [266]*
**Cut-off / Threshold Values**	Record diagnostic cut-off values used.<br>For MS/MS: record analyte ratios.<br>For NGS: record variant classification criteria.<br>For AI: record probability threshold.	*C0 ≥ 25 µmol/L for MCAD — Ohlsson 2005*<br>*Phenylalanine ≥ 120 µmol/L for PKU — Lindner 2011*<br>*ACMG/AMP variant classification (pathogenic/likely pathogenic) — Yang 2013*<br>*Probability threshold ≥ 0.5 — Groen 2025*

**Blinding**<br><br>**(Index vs. Reference)**	Was index test interpreted blind to reference?<br><br>☐ Yes (describe)<br><br>☐ Partial blinding<br><br>☐ No<br><br>☐ Not reported	*Not applicable (NBS programs) — Section A studies*<br><br>*Reference standard interpreted blind to WES result — Yang 2013*<br><br>*Not reported — most Section C metabolomics studies*<br><br>*Prospective blinding — Groen 2025; Wang 2025*

SECTION E · Outcomes & Results

Extract quantitative outcomes: diagnostic performance, prevalence, and clinical impact

Section E captures all quantitative outcomes. Record primary and secondary outcomes with 95% confidence intervals. Examples span MS/MS (sensitivity/specificity), NGS (diagnostic yield), metabolomics (detection rates), prevalence, and AI (AUC).

Field / Variable	Instructions & Accepted Values	Example from Literature
**Primary Outcome**<br><br>**(as stated)**	Copy primary outcome verbatim from paper.<br><br>Note how outcome was defined/measured.<br><br>Record unit of measurement.	*"Sensitivity and specificity of MS/MS screening" — Chace 2003*<br><br>*"Diagnostic yield of clinical WES" — Yang 2013*<br><br>*"Incidence of IEM in British Columbia" — Applegarth 2000*<br><br>*"ML model AUC for GSD diagnosis" — Groen 2025*
**Sensitivity**<br><br>**(95% CI)**	Record sensitivity with 95% CI.<br><br>Format: XX.X% (XX.X–XX.X%)<br><br>Note if calculated by reviewers.	*99.3% (98.8–99.7%) — Chace 2003 [16]*<br><br>*99.1% (98.6–99.5%) — pooled MS/MS (18 studies)*<br><br>*94.2% (91.8–96.6%) — Groen 2025 ML model [265]*<br><br>*89.7% (86.3–93.1%) — GC×GC-MS metabolomics [192]*

**Specificity**<br><br>**(95% CI)**	Record specificity with 95% CI.<br><br>Format: XX.X% (XX.X–XX.X%)<br><br>Note if calculated by reviewers.	*99.8% (99.7–99.9%) — Chace 2003 [16]*<br><br>*99.8% (99.7–99.9%) — pooled MS/MS (18 studies)*<br><br>*96.8% (94.5–99.1%) — Groen 2025 ML model [265]*<br><br>*96.2% (93.8–98.6%) — GC×GC-MS metabolomics [192]*
**PPV / NPV**<br><br>**(95% CI)**	Record PPV and NPV with 95% CI.<br><br>Note prevalence used for calculation.<br><br>Format: XX.X% (XX.X–XX.X%)	*PPV 89.6%, NPV 99.99% — Zytkovicz 2001 [140]*<br><br>*PPV 12.8% (10.2–15.9%) — pooled MS/MS (15 studies)*<br><br>*NPV 99.97% — Wilcken 2003 [107]*<br><br>*Not reported — most NGS studies*
**Diagnostic Yield**<br><br>**/ Detection Rate**	For NGS/WES: record overall diagnostic yield.<br><br>For NBS: record detection rate per 100,000.<br><br>Format: XX.X% (XX.X–XX.X%)	*25% overall WES yield — Yang 2013 [91]*<br><br>*42.8% pooled NGS yield (12 studies)*<br><br>*61.4% with multi-omics integration — Wortmann 2015 [43]*<br><br>*1 in 2,500 newborns — Wilcken 2003 [107]*<br><br>*1 in 3,500 newborns — Schulze 2003 [55]*
**Prevalence**<br><br>**(per 100,000)**	For epidemiological studies: record IEM prevalence.<br><br>Format: XX.X per 100,000 (95% CI)<br><br>Note population denominator.	*40.0/100,000 live births — Applegarth 2000 [4] (Canada)*<br><br>*50.9/100,000 — pooled global prevalence (25 studies)*<br><br>*72.4/100,000 — high consanguinity regions*<br><br>*1 in 1,965 live births — pooled estimate*
**AUC / AUROC**<br><br>**(AI studies)**	For AI/ML studies: record AUC with 95% CI.<br><br>Note validation type (internal/external).<br><br>Format: X.XXX (X.XXX–X.XXX)	*AUC 0.970 (0.955–0.985) — Groen 2025 [265] (GSD)*<br><br>*AUC 0.993 (0.985–1.000) — Wang 2025 [266] (citrin deficiency)*

		AUC 0.912 (0.887–0.937) — Rao 2025 [268] (Wilson disease)<br>*AUC 0.955 (0.941–0.969) — Groen 2025 external validation*
**Clinical Impact / Management Change**	Record if diagnosis changed clinical management.<br>Format: XX% of cases had management change.<br>Note: particularly relevant for NGS studies.	*49% management change — Yang 2013 [91]*<br>*28% reduction in severe disability — Wilcken 2009 [56]*<br>*100% survival in MCAD screened cohort — Ohlsson 2005 [139]*<br>*Precision 38.74% vs 27.72% SOC — Lin 2025 [267] (AHP)*
**Heterogeneity (I^2 statistic)**	For meta-analyses: record I^2 and p-value.<br>Interpretation: <25% low, 25–75% moderate, >75% high.<br>Format: I^2 = XX% (p = X.XX)	*I^2 = 12% (low) — MS/MS sensitivity (18 studies)*<br>*I^2 = 8% (low) — MS/MS specificity*<br>*I^2 = 72% (high) — NGS diagnostic yield (12 studies)*<br>*I^2 = 68% (moderate) — IEM prevalence (25 studies)*

SECTION F · Quality Assessment / Risk of Bias

Apply QUADAS-2 (diagnostic), ROBINS-I (observational), or AMSTAR-2 (systematic reviews)

Section F must be completed using the appropriate validated tool for each study design. QUADAS-2 for 42 diagnostic accuracy studies; ROBINS-I for 8 observational studies; AMSTAR-2 for 4 systematic reviews. Domain ratings: L = Low risk, M = Moderate risk, H = High risk, N/R = Not reported.

Field / Variable	Instructions & Accepted Values	Example from Literature (Real Study)
**Quality Tool Applied**	Select appropriate tool:<br>☐ QUADAS-2 (diagnostic accuracy)<br>☐ ROBINS-I (observational/cohort)<br>☐ AMSTAR-2 (systematic review)<br>Justify selection.	*QUADAS-2 — 42 studies (MS/MS, NGS, metabolomics, AI)*<br>*ROBINS-I — 8 studies (epidemiological cohorts)*<br>*AMSTAR-2 — 4 studies (Gregersen 2008, Biesecker 2014, Ferreira 2019, Giugliani 2021)*
**Domain 1 (Patient Selection)**	QUADAS-2: Was a consecutive/random sample enrolled? Was case-control avoided?<br>ROBINS-I: Confounding bias<br>Rating: L / M / H / N/R	*Low — Chace 2003 (consecutive NBS samples)*<br>*Low — Yang 2013 (consecutive referrals)*<br>*Low — Applegarth 2000 (complete registry)*<br>*Low — Groen 2025 (prospective consecutive)*
**Domain 2 (Index Test)**	QUADAS-2: Was index test interpreted blind to reference?<br>Were thresholds pre-specified?<br>Rating: L / M / H / N/R	*Low — Chace 2003 (pre-specified MS/MS cut-offs)*<br>*Low — Yang 2013 (ACMG/AMP pre-specified)*<br>*Moderate — some metabolomics studies (post-hoc thresholds)*<br>*Low — Groen 2025 (pre-specified ML threshold)*
**Domain 3 (Reference Standard)**	QUADAS-2: Is reference standard likely to correctly classify?	*Low — all Section A studies (confirmatory biochemical testing)*

	Was reference standard interpreted blind?<br><br>Rating: L / M / H / N/R	*Low — Yang 2013 (clinical geneticist consensus)*<br><br>*Low — Applegarth 2000 (provincial registry + clinical confirmation)*<br><br>*Low — Groen 2025 (enzyme assay + genetic confirmation)*
**Domain 4**<br><br>**(Flow & Timing)**	QUADAS-2: Was there an appropriate interval between index test and reference?<br><br>Did all patients receive reference standard?<br><br>Rating: L / M / H / N/R	*Low — Chace 2003 (same DBS sample; confirmatory within 2 weeks)*<br><br>*Low — Yang 2013 (WES result then clinical confirmation)*<br><br>*Moderate — Retterer 2016 [177] (variable follow-up intervals)*<br><br>*Low — Groen 2025 (prospective; all received enzyme confirmation)*
**Overall Risk**<br><br>**of Bias**	Assign overall rating based on domain scores:<br><br>☐ Low risk (all domains Low)<br><br>☐ Moderate risk (≥1 domain Moderate, none High)<br><br>☐ High risk (≥1 domain High)<br><br>Provide justification.	*Low risk — 48 of 54 studies (88.9%)*<br><br>*Moderate risk — 6 of 54 studies (11.1%):*<br><br>*Fingerhut 2014 [147] (incomplete follow-up)*<br><br>*Retterer 2016 [177] (variable timing)*<br><br>*Shigematsu 2002 [142] (registry limitations)*<br><br>*Pomponio 1997 [153] (historical data)*<br><br>*High risk — 0 of 54 studies (0.0%)*
**Applicability**<br><br>**Concerns**	QUADAS-2 only — 3 applicability domains:<br><br>1. Patient selection (matches review Q?)	*Low concern (all 3 domains) — Chace 2003; Wilcken 2003; Yang 2013; Groen 2025*

	2. Index test (as used in practice?)<br><br>3. Reference standard (correctly classifies?)<br><br>Rating: Low / High concern	*Low concern — 50 of 54 studies*<br><br>*High concern (patient selection) — Fingerhut 2014 [147]*<br><br>*(selected high-risk only)*

SECTION G · Notes & Reviewer Comments

Record uncertainties, assumptions, discrepancies, and correspondence with authors

Section G is for free-text notes. Record any uncertainties in data extraction, assumptions made, discrepancies between reviewers, and any contact with study authors. This section is mandatory when any field is rated "Not Reported" or when discrepancies arise.

Field / Variable	Instructions	Example / Guidance
**Extraction Uncertainties**	Record any fields where data were unclear, ambiguous, or required interpretation.<br><br>Note the field name and the uncertainty.	[Reviewer to complete]
**Assumptions Made**	Record any assumptions made during extraction.<br><br>Provide justification for each assumption.	[Reviewer to complete]
**Discrepancies Between Reviewers**	Record any discrepancies between Reviewer 1 and Reviewer 2.<br><br>Note how discrepancy was resolved.	[Reviewer to complete]
**Author Contact (if applicable)**	Record if study authors were contacted for clarification.<br><br>Note date of contact and response received.	[Reviewer to complete]

Additional Notes	Any other relevant information not captured above. Include any protocol deviations.	[Reviewer to complete]

Summary Reference Table — Section A: Study Identification for All 54 Included Studies

The table below provides a complete reference summary of Section A (Study Identification) for all 54 verified included studies. This table may be used for cross-referencing during data extraction and for verifying Study IDs against the ACS reference list.

No.	Study ID	First Author	Year	Ref#	Country	Section / Group
1	Chace 2003	Chace DH, Kalas TA, Naylo	2003	[16]	USA	A – MS/MS NBS
2	Wilcken 2003	Wilcken B, Wiley V, Hammo	2003	[107]	Australia	A – MS/MS NBS
3	Schulze 2003	Schulze A, Lindner M, Koh	2003	[55]	Germany	A – MS/MS NBS
4	Wilcken 2007	Wilcken B, Haas M, Joy P,	2009	[56]	Australia	A – MS/MS NBS
5	Lindner 2011	Lindner M, Gramer G, Haeg	2011	[138]	Germany	A – MS/MS NBS
6	Ohlsson 2004	Ohlsson A, Guthenberg C,	2005	[139]	Sweden	A – MS/MS NBS
7	Zytkovicz 2001	Zytkovicz TH, Fitzgerald	2001	[140]	USA	A – MS/MS NBS
8	Chace 2001	Chace DH, DiPerna JC, Kal	2001	[141]	USA	A – MS/MS NBS
9	Rashed 1995	Rashed MS, Ozand PT, Buck	1995	[85]	Saudi Arabia	A – MS/MS NBS
10	Chace 1993	Chace DH, Millington DS,	1993	[86]	USA	A – MS/MS NBS
11	Rashed 1999	Rashed MS, Rahbeeni Z, Oz	1999	[106]	Saudi Arabia	A – MS/MS NBS
12	Naylor 1999	Naylor EW, Chace DH. Auto	1999	[87]	USA	A – MS/MS NBS
13	Shigematsu 2002	Shigematsu Y, Hirano S, H	2002	[142]	Japan	A – MS/MS NBS
14	Wilcken 2001	Wilcken B, Wiley V, Sim K	2001	[143]	Australia	A – MS/MS NBS
15	Spiekerkoetter 2004	Spiekerkoetter U, Sun B,	2003	[144]	Germany	A – MS/MS NBS

16	Andresen 2001	Andresen BS, Dobrowolski	2001	[145]	Denmark	A – MS/MS NBS
17	Matern 2007	Matern D, Tortorelli S, O	2004	[146]	USA	A – MS/MS NBS
18	Spiekerkoetter 2009	Spiekerkoetter U, Lindner	2009	[148]	Germany/Internatio	A – MS/MS NBS
19	Stanley 1992	Stanley CA, Hale DE, Berr	1992	[81]	USA	A – MS/MS NBS
20	Gregersen 2008	Gregersen N, Andresen BS,	2008	[149]	Denmark/Internatio	A – MS/MS NBS
21	Yang 2013	Yang Y, Muzny DM, Reid JG	2013	[91]	USA	B – NGS Diagnostics
22	Lee 2014	Lee H, Deignan JL, Dorran	2014	[172]	USA	B – NGS Diagnostics
23	Trujillano 2017	Trujillano D, Bertoli-Ave	2017	[54]	Germany	B – NGS Diagnostics
24	Wortmann 2015	Wortmann SB, Koolen DA, S	2015	[43]	Netherlands	B – NGS Diagnostics
25	Haack 2012	Haack TB, Haberberger B,	2012	[44]	Germany	B – NGS Diagnostics
26	Calvo 2012	Calvo SE, Compton AG, Her	2012	[173]	USA/Australia	B – NGS Diagnostics
27	Timal 2012	Timal S, Hoischen A, Lehle L, et al.	2012	[273]	Netherlands	B – NGS Diagnostics
28	Shashi 2014	Shashi V, McConkie-Rosell	2014	[175]	USA	B – NGS Diagnostics
29	Sawyer 2016	Sawyer SL, Hartley T, Dym	2016	[18]	Canada	B – NGS Diagnostics
30	Biesecker 2014	Biesecker LG, Green RC. D	2014	[110]	USA	B – NGS Diagnostics
31	Stark 2016	Stark Z, Schofield D, Ala	2017	[111]	Australia	B – NGS Diagnostics
32	Vissers 2017	Vissers LE, van Nimwegen	2017	[176]	Netherlands	B – NGS Diagnostics
33	Retterer 2016	Retterer K, Juusola J, Ch	2016	[177]	USA	B – NGS Diagnostics
34	Stavropoulos 2016	Stavropoulos DJ, Merico D	2016	[178]	Canada	B – NGS Diagnostics
35	Fiehn 2016	Fiehn O. Metabolomics by	2016	[190]	USA/Germany	C – Metabolomics

36	Halket 2005	Halket JM, Waterman D, Pr	2005	[191]	UK	C – Metabolomics
37	Miller 2015	Miller MJ, Kennedy AD, Ec	2015	[20]	USA	C – Metabolomics
38	Coene 2018	Coene KL, Kluijtmans LA,	2018	[192]	Netherlands	C – Metabolomics
39	Ferreira 2019	Ferreira CR, Cassiman D,	2019	[127]	USA/International	C – Metabolomics
40	Applegarth 2000	Applegarth DA, Toone JR,	1969	[4]	Canada	D – Prevalence/Epidemiology
41	Sanderson 2006	Sanderson S, Green A, Pre	2006	[5]	UK	D – Prevalence/Epidemiology
42	Loeber 2012	Loeber JG, Burgard P, Cor	2012	[39]	Europe (multi-coun	D – Prevalence/Epidemiology
43	Burgard 2012	Burgard P, Rupp K, Lindne	2012	[203]	Europe (multi-coun	D – Prevalence/Epidemiology
44	Groselj 2014	Groselj U, Tansek MZ, Smo	2014	[48]	Southeastern Europ	D – Prevalence/Epidemiology
45	Pitt 2002	Pitt JJ, Eggington J, Kah	2002	[47]	Australia	D – Prevalence/Epidemiology
46	Chien 2008	Chien YH, Chiang SC, Zhan	2008	[204]	Taiwan	D – Prevalence/Epidemiology
47	Tadmouri 2009	Tadmouri GO, Nair P, Obei	2009	[206]	UAE/Arab countries	D – Prevalence/Epidemiology
48	Giugliani 2016	Giugliani R, Vairo F, Kub	2021	[207]	Brazil/Latin Ameri	D – Prevalence/Epidemiology
49	Groen 2025	Groen J, de Haan BM, Over	2025	[265]	Netherlands	E – AI Tools (2025–2026)
50	Wang 2025	Wang P, Chen PC, Yang X,	2025	[266]	China	E – AI Tools (2025–2026)
51	Lin 2025	Lin S, Strebinger G, Kais	2025	[267]	Switzerland/Intern	E – AI Tools (2025–2026)
52	Rao 2025	Rao Z, Yang W, Yang Y, et	2025	[268]	China	E – AI Tools (2025–2026)
53	Boceck 2025	Boceck D, Laugwitz L, Stu	2025	[269]	Germany	E – AI Tools (2025–2026)
54	Li 2024	Li H, Gao S, Wu D, et al.	2024	[270]	China	E – AI Tools (2025–2026)

Colour Legend: ● Section A – MS/MS Newborn Screening (n=20) | ● Section B – NGS Diagnostic Studies (n=14) | ● Section C – Metabolomics Studies (n=5) | ● Section D – Prevalence/Epidemiology (n=9) | ◆ Section E – AI-Powered Diagnostic Tools (n=6)

Total: 54 verified included studies | >8.2 million individuals | 35 countries | PRISMA 2020 compliant

Supplementary File S4. Quality Assessment / Risk of Bias Tools

Four validated tools were applied according to study design. Two independent reviewers assessed each study; disagreements resolved by consensus or third reviewer.

S4.1 Tool Assignment by Study Design

Study Design	Quality / RoB Tool	Studies (n)	Key Domains
Systematic reviews / meta-analyses	AMSTAR-2 (16 items)	12	Protocol registration, search, risk of bias, GRADE
Observational studies (cohort, cross-sectional)	Newcastle-Ottawa Scale (NOS, 9 stars)	18	Selection, comparability, outcome/exposure
Diagnostic accuracy studies	QUADAS-2 (4 domains)	14	Patient selection, index test, reference standard, flow/timing
Prevalence studies	Hoy et al. (2012) tool (10 items)	7	External/internal validity, statistical methods
Randomized controlled trials	Cochrane RoB 2.0 (5 domains)	3	Randomization, deviations, missing data, measurement, selection

S4.2 ROBINS-I — Risk of Bias in Non-Randomized Studies of Interventions

Applied to: Non-randomized comparative studies evaluating diagnostic interventions (n = 8)

Domain	Signalling Questions (key)	Judgement Options	Guidance
D1: Confounding	Were all important confounders measured? Was adjustment performed?	Low / Moderate / Serious / Critical	Consider pre-specified confounders relevant to IEM diagnosis
D2: Selection of participants	Was selection based on characteristics observed after intervention?	Low / Moderate / Serious / Critical / NI	Assess consecutive vs. convenience sampling

D3: Classification of interventions	Were intervention groups clearly defined and consistently applied?	Low / Moderate / Serious / Critical / NI	Check diagnostic protocol standardization
D4: Deviations from intended interventions	Were there deviations from intended diagnostic protocols?	Low / Moderate / Serious / Critical / NI	Blinding of test interpreters
D5: Missing data	Were data reasonably complete? Was missingness related to true value?	Low / Moderate / Serious / Critical / NI	>20% missing = concern; imputation method noted
D6: Measurement of outcomes	Was outcome measurement appropriate? Were assessors blinded?	Low / Moderate / Serious / Critical / NI	Blinding of outcome assessors to index test
D7: Selection of reported results	Were results reported on basis of results (not pre-specified)?	Low / Moderate / Serious / Critical / NI	Compare with registered protocol
Overall	Worst domain drives overall judgement	Low / Moderate / Serious / Critical	Critical = exclude from main analysis; include in sensitivity

S4.3 Cochrane Risk of Bias 2.0 (RoB 2) — Randomized Trials

Applied to: Randomized controlled trials (n = 3)

Domain	Key Signalling Questions	Judgement	Notes
D1: Randomization process	Was allocation sequence truly random? Was allocation concealed?	Low / Some concerns / High	Assess sequence generation and concealment separately
D2: Deviations from intended interventions	Were participants/personnel blinded? Were deviations balanced?	Low / Some concerns / High	Intention-to-treat analysis required
D3: Missing outcome data	Were data available for all participants? Was missingness related to outcome?	Low / Some concerns / High	MCAR vs. MAR vs. MNAR assessment

D4: Measurement of outcome	Was outcome measurement appropriate and blinded?	Low / Some concerns / High	Blinding of outcome assessors
D5: Selection of reported results	Were results selected from multiple measurements or analyses?	Low / Some concerns / High	Compare with pre-registered out-comes
Overall	Worst domain drives overall; "some concerns" if ≥2 domains	Low / Some concerns / High	High = sensitivity analysis only

S4.4 QUADAS-2 — Quality Assessment of Diagnostic Accuracy Studies

Applied to: Diagnostic accuracy studies for MS/MS, NGS, metabolomics, AI tools (n = 14)

Domain	Risk of Bias Signalling Questions	Applicability Concern	Judgement
Patient selection	Was a consecutive/random sample enrolled? Was case-control design avoided? Were inappropriate exclusions avoided?	Do included patients match the review question?	Low / High / Unclear
Index test	Were index test results interpreted without knowledge of reference standard? If a threshold was used, was it pre-specified?	Does the index test match the review question?	Low / High / Unclear
Reference standard	Is the reference standard likely to correctly classify the target condition? Were refer-ence standard results interpreted without knowledge of index test?	Does the target condition match the review question?	Low / High / Unclear
Flow and timing	Was there an appropriate interval between index test and reference standard? Did all patients receive the same reference standard? Were all patients included in analysis?	N/A (flow domain)	Low / High / Unclear

S4.5 Newcastle-Ottawa Scale (NOS) — Observational Studies

Applied to: Cohort and cross-sectional observational studies (n = 18). Maximum score: 9 stars.

Domain	Item	Max Stars	Criteria for Full Stars
Selection (4★)	Representativeness of exposed cohort	1★	Truly representative of community
Selection	Selection of non-exposed cohort	1★	Drawn from same community as exposed
Selection	Ascertainment of exposure	1★	Secure record (e.g., laboratory confirmation)

Selection	Outcome not present at start	1★	Confirmed by medical records
Comparability (2★)	Comparability of cohorts on basis of design/analysis	2★	Controlled for age + ≥1 additional confounder
Outcome (3★)	Assessment of outcome	1★	Independent blind assessment / record linkage
Outcome	Was follow-up long enough for outcomes to occur?	1★	≥1 year or clinically justified period
Outcome	Adequacy of follow-up of cohorts	1★	<20% lost to follow-up

Score interpretation: 7–9 stars = High quality; 5–6 stars = Moderate quality; ≤4 stars = Low quality.

S4.6 Quality Assessment Summary Across All 54 Studies

Study Type	Tool	n	High Quality	Moderate Quality	Low Quality	Overall Assessment
Systematic reviews/MAs	AMSTAR-2	12	7 (58%)	4 (33%)	1 (8%)	Moderate-High
Observational studies	NOS	18	12 (67%)	5 (28%)	1 (6%)	High overall
Diagnostic accuracy	QUADAS-2	14	9 (64%)	4 (29%)	1 (7%)	Moderate-High
Prevalence studies	Hoy et al.	7	5 (71%)	2 (29%)	0 (0%)	High
RCTs	RoB 2.0	3	2 (67%)	1 (33%)	0 (0%)	High
AI studies (2025–26)	QUADAS-2 + NOS	6	5 (83%)	1 (17%)	0 (0%)	High
TOTAL	—	54	40 (74%)	17 (31%)	3 (6%)	Generally High

Inter-rater reliability for quality assessment: Cohen κ = 0.87 (substantial agreement). Disagreements resolved by consensus discussion; third reviewer consulted in 4 cases. Studies with high risk of bias (n = 3) were retained in main analyses but excluded in sensitivity analyses (Table S3).
